# North Pontic crossroads: Mobility in Ukraine from the Bronze Age to the early modern period

**DOI:** 10.1126/sciadv.adr0695

**Published:** 2025-01-08

**Authors:** Lehti Saag, Olga Utevska, Stanislav Zadnikov, Iryna Shramko, Kyrylo Gorbenko, Mykola Bandrivskyi, Dmytro Pavliv, Igor Bruyako, Denys Grechko, Vitalii Okatenko, Gennadi Toshev, Svitlana Andrukh, Vira Radziyevska, Yurii Buynov, Viktoriia Kotenko, Oleksandr Smyrnov, Oleg Petrauskas, Borys Magomedov, Serhii Didenko, Anatolii Heiko, Roman Reida, Serhii Sapiehin, Viktor Aksonov, Oleksii Laptiev, Svyatoslav Terskyi, Viacheslav Skorokhod, Vitalii Zhyhola, Yurii Sytyi, Mari Järve, Christiana Lyn Scheib, Kyriaki Anastasiadou, Monica Kelly, Mia Williams, Marina Silva, Christopher Barrington, Alexandre Gilardet, Ruairidh Macleod, Pontus Skoglund, Mark G. Thomas

**Affiliations:** ^1^ UCL Genetics Institute, Research Department of Genetics, Evolution and Environment, University College London, London WC1E 6BT, UK.; ^2^ Estonian Biocentre, Institute of Genomics, University of Tartu, Tartu 51010, Estonia.; ^3^ Department of Genetics and Cytology, V.N. Karazin Kharkiv National University, Kharkiv 61000, Ukraine.; ^4^ Museum of Archaeology, V.N. Karazin Kharkiv National University, Kharkiv 61022, Ukraine.; ^5^ Department of Historiography, Source Studies and Archaeology, V.N. Karazin Kharkiv National University, Kharkiv 61022, Ukraine.; ^6^ Department of History, Petro Mohyla Black Sea National University, Mykolaiv 54003, Ukraine.; ^7^ Department of Archaeology, Ivan Krypiakevych Institute of Ukrainian Studies, National Academy of Sciences of Ukraine, Lviv 79026, Ukraine.; ^8^ South Ukrainian K. D. Ushinsky National Pedagogical University State Institution, Odesa 65000, Ukraine.; ^9^ Department of Archaeology of the Early Iron Age, Institute of Archaeology, National Academy of Sciences of Ukraine, Kyiv 04210, Ukraine.; ^10^ State Enterprise “Research Center ‘Protective Archaeological Service of Ukraine,’” Institute of Archeology, National Academy of Sciences of Ukraine, Kyiv 04210, Ukraine.; ^11^ Educational and Scientific Laboratory of Archaeological Research, Zaporizhzhia National University, Zaporizhzhia 69061, Ukraine.; ^12^ Department of Ancient archaeology, Institute of Archaeology, National Academy of Sciences of Ukraine, Kyiv 04210, Ukraine.; ^13^ Department of Archaeology of Early Slavs, Institute of Archaeology, National Academy of Sciences of Ukraine, Kyiv 04210, Ukraine.; ^14^ Research Department of Archaeology of the Early Iron Age, National Museum of History of Ukraine, Kyiv 02000, Ukraine.; ^15^ Department of Accounting and Research of Archaeological Monuments and Survey of Land Plots, Communal institution “Center for Protection and Research of Archaeological Monuments” of the Poltava Regional Council, Poltava 36000, Ukraine.; ^16^ Anton Makarenko Museum, Poltava Regional Makarenko Scientific Lyceum, Kovalivka 38701, Ukraine.; ^17^ Department of Archaeology, Municipal Institution “M.F. Sumtsov Kharkiv Historical Museum” of the Kharkiv Regional Council, Kharkiv 61003, Ukraine.; ^18^ Department of History of Ukraine and Ethnocommunications, Lviv Polytechnic National University, Lviv 79013, Ukraine.; ^19^ Department of Old Rus and Medieval Archeology, Institute of Archaeology, National Academy of Sciences of Ukraine, Kyiv 04210, Ukraine.; ^20^ D.Ya. Samokvasov Research Center of Archeology and Ancient and Early Modern History of the Northern Left Bank, T.H. Shevchenko National University “Chernihiv Colehium,” Chernihiv 14000, Ukraine.; ^21^ Department of Zoology, University of Cambridge, Cambridge CB2 3EJ, UK.; ^22^ Ancient Genomics Laboratory, The Francis Crick Institute, London NW1 1AT, UK.; ^23^ Bioinformatics and Biostatistics, The Francis Crick Institute, London NW1 1AT, UK.; ^24^ Centre for Palaeogenetics, Stockholm 106 91, Sweden.; ^25^ Department of Archaeology, University of Cambridge, Cambridge CB2 3DZ, UK.

## Abstract

The North Pontic region, which encompasses present-day Ukraine, was a crossroads of migration, connecting the vast Eurasian Steppe with Central Europe. We generated shotgun-sequenced genomic data for 91 individuals dating from around 7000 BCE to 1800 CE to study migration and mobility history in the region, with a particular focus on historically attested migrating groups during the Iron Age and the medieval period. We infer a high degree of temporal heterogeneity in ancestry, with fluctuating genetic affinities to different present-day Eurasian groups. We also infer high heterogeneity in ancestry within geographically, culturally, and socially defined groups. Despite this, we find that ancestry components which are widespread in Eastern and Central Europe have been present in the Ukraine region since the Bronze Age. In short, our study reveals a diverse range of ancestries in the Ukraine region through time as a result of frequent movements, assimilation, and contacts.

## Teaser

Genomes from Bronze Age to early modern Ukraine reveal a high degree of temporal as well as within-group genetic heterogeneity.

## INTRODUCTION

Migration has been a major factor shaping human societies, culture, biology, and genomes through time. Previous ancient DNA (aDNA) research indicates that, to a first order of approximation, the genomes of present-day Europeans comprise ancestries from three major Holocene groups of people (*1*, *2*): (i) local hunter-gatherers (HGs); (ii) near Eastern early farmers, arriving in Europe ~8000 years ago (*3*); (iii) steppe pastoralists, who migrated into Europe ~5000 years ago (*4*). However, the detailed genetic history of any given region is necessarily more complex, calling for more focused and local-scale studies. One such and so far relatively understudied region is present-day Ukraine in the Northern Black Sea (Pontic) region, which is historically and archaeologically known as a contact zone between European and Asian populations. Archaeological and genetic data indicate not only ancestries from the above three broad-scale sources but that it was the first area with early farmer habitation that the steppe pastoralists reached on their westward migration (*4*–*7*). Subsequent admixture between these two groups is particularly interesting as it was quickly followed by the emergence of the Corded Ware, Sintashta, Andronovo, and Zrubna (Srubnaya) cultures over wide areas of central Eurasia (*1*, *2*, *8*). Furthermore, because southern Ukraine forms part of the Eurasian Steppe, which reaches from Hungary in the west all the way to Northeast China in the east, the area has been in the path of extensive if punctuated genetic and cultural flow (*9*). The accessibility of the Ukrainian Steppe, the presence of an extensive hydrological system, the abundance of raw materials, and the combination of fertile forest-steppe soils with open steppe spaces, large forests, and mountain ranges attracted not only various nomadic groups but also representatives of ancient sedentary civilizations who founded colonies on the Black Sea coast (*9*).

**Fig. 1.**
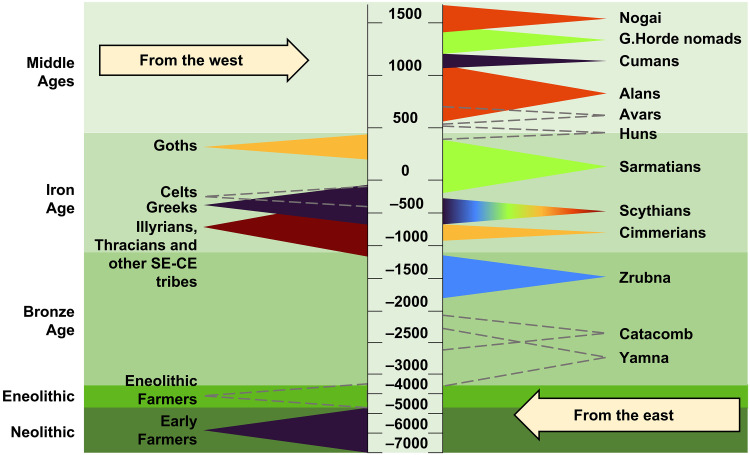
Schematic of migrating groups from the west and the east in Ukraine based on archaeological material. Negative values on the timeline denote years BCE, positive values denote years CE. The color scheme of the shapes matches the symbols on Figs. 2, 3, and 5 as well as figs. S1 and S2. Gray dashed lines denote groups that are not sampled in this study.

For centuries, migration took place in the steppe and forest-steppe belt of Ukraine, moving in several directions (Fig. 1). These migrations were driven by various processes, including periodic aridization, the development of new subsistence strategies and economies, intertribal cultural contacts and conflicts, trade, demographic pressures, and expansion of nomad influence zones. Major migration flows came from the Carpathian-Danubian region, the Southern Urals and Volga region, Central Asia, the North Caucasus, etc., and intensive population movements also occurred within the territory of Ukraine (*10*–*12*). At the end of the Bronze Age and beginning of the Early Iron Age, the most archaeologically conspicuous activities of the North Pontic steppes were associated with Cimmerians and their military campaigns in Asia Minor (*13*). The Cimmerians were followed by Scythians and Sarmatians, Early Iron Age political and military tribal unions (*9*, *14*) with variable combinations of local and East Asian ancestry, as indicated by previous aDNA studies (*7*, *15*–*18*). At this time, the Northern Black Sea coast was covered with a network of urbanized Greek colonies (*19*, *20*). In the forest-steppe zone, the contemporary settled populations were associated with the previous Tshinets Cultural Circle (*21*) (including Lusatian and Vysotska cultures), as well as with Central European influences of Hallstatt and La Tène periods (Illirians, Thracians, and Celts) (*22*–*25*). According to written and archaeological sources, peoples that are considered the predecessors of the Slavs (associated with the Zarubinetska culture) had already been present in the Ukraine region during the La Tène and Roman periods, from the third century (c.) BCE onward (*26*). The beginning of the Migration Period in the Ukraine region is associated with the arrival of Germanic tribes such as Goths, and the formation of the multiethnic Chernyakhiv culture, which included other peoples who already inhabited the region (*27*, *28*). In the second to fourth c. CE, the Huns—nomadic people from Central Asia—appeared in the North Pontic steppe, and their westward migration led to notable economic, cultural, and social changes in Europe. This period is associated with the emergence of a new ethnolinguistic group, the Slavs, who spread throughout much of eastern Europe during fifth to seventh c. CE (*29*, *30*). In the 8th to 10th c. CE, а substantial part of Ukraine was under the control of Khazar Khaganate. In Ukrainian archaeology, this is represented by the Saltiv culture, which is thought to have been shared among multiple ethnic groups (Alans, Bulgars, Turks, Slavs, Magyars, etc.). During the same period, there was a process of unification of the Slavic tribes, and in the ninth c. CE, the state of Kievan Rus was formed (*31*). The development of Slavic statehood took place against the background of constant nomadic incursions from the east. In the period from the 11th to the 13th c. CE, waves of Pechenegs, Torques, and Cumans entered the North Pontic region from Central Asia, and the most substantial invasion in terms of military strength and consequences was that of the Mongols of the Golden Horde in the 13th c. CE (*12*). By the 15th c. CE, remnants of the Golden Horde population, such as the Nogai, were still living in the North Pontic steppes (*32*). Since the 16th c. CE, Slavs have been the majority ethnolinguistic group in the region of Ukraine (*12*, *31*).

To date, published ancient genomic data from Ukraine are available mostly for Mesolithic to Bronze Age individuals, some Iron Age Scythians, and only a few individuals from other periods (*6*, *7*, *16*, *33*–*40*). Because of the need to distinguish between groups of individuals and for brevity, we will refer to individuals by the archaeological culture context with which they have been associated. It should not be assumed that there is a direct link between culture and genetic ancestry (*41*). Genomic studies of HGs in Ukraine indicate closer genetic affinities to Eastern, rather than Western European HGs (*6*, *36*, *40*). Early farmers in the region, represented by Trypillia and Globular Amphora culture–associated individuals, show indications of admixture with HGs (*6*, *35*). Eneolithic Cernavodă I and Usatove culture–associated individuals display admixture with steppe ancestry groups (*38*). Genomic data from four Yamna culture–associated individuals have been published, with one displaying admixture with European early farmers (*6*, *7*). There is considerable heterogeneity in the 13 Scythian genomes available, with various combinations of early farmer, HG/steppe, and also East Asian ancestry present (*7*, *16*). The three published Chernyakhiv individuals resemble modern Europeans but do not form a genetically homogeneous group (*7*). These results provide substantial evidence of unresolved genetic structure and population discontinuity in Ukraine and fragmented relationships between genetic ancestry and material culture associations, starting from the Neolithic, pointing to a need for more paleogenomic data. Furthermore, no data are available from the Middle Ages; such data would extend our understanding of the demographic history of the area. In this study, we shed light on the demographic history of Ukraine from the Bronze Age to the early modern period. We set out to examine the genetic ancestries of people living in the North Pontic region during different time periods and associated with various cultural groups. We do not encompass the entirety of local cultures but focus on those introduced by migrants, which include a wide range of nomadic warrior groups (Fig. 1). We investigate whether individuals associated with specific cultural groups are genetically homogeneous or whether genetic structure exists within them. More specifically, we assess the extent to which admixture occurs between local and migrant groups and to what extent autochthonous peoples are culturally assimilated into migrant groups. For Scythian-associated individuals, we explore genetic differences between individuals with nomad versus local and elite versus non-elite archaeological assignments as well as between different areas of Ukraine.

## RESULTS

### Samples and archaeological background

**Fig. 2.**
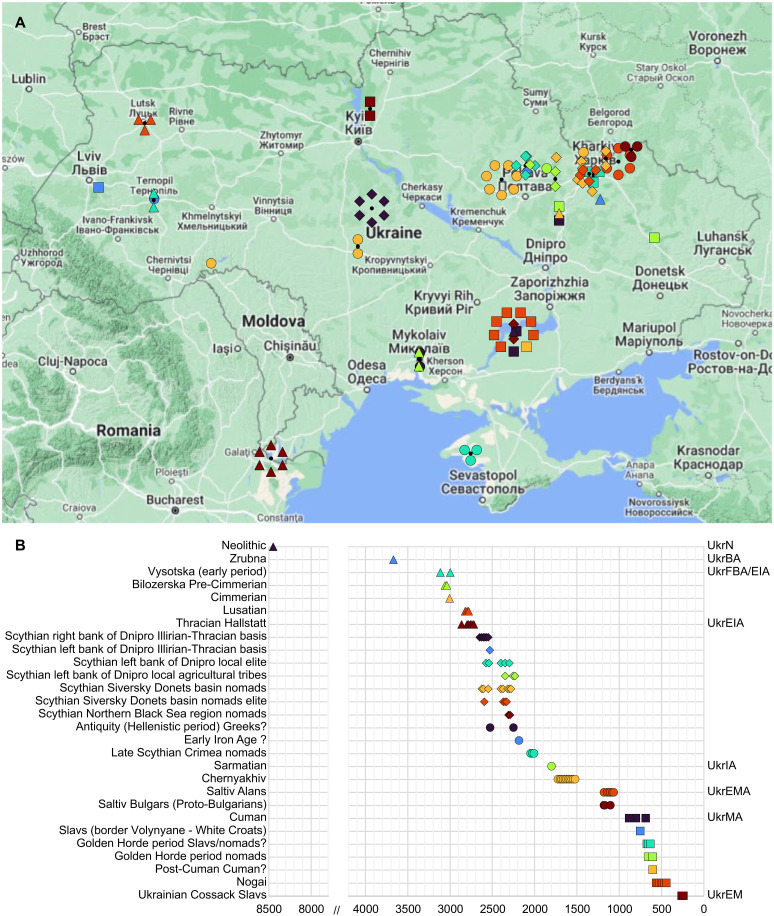
Map of the geographical locations of the individuals of this study and timeline showing the dates of individuals in archaeological groups. Symbols on (**A**) the map correspond to those on (**B**) the timeline. The dates (years before the present) used on the timeline are midpoints of the 95% calibrated date estimates or archaeological date range estimates with jitter.

We extracted DNA from the apical tooth roots and bone fragments of 128 individuals from 33 archaeological sites in present-day Ukraine (data S1 and Supplementary Text). The 91 individuals that were chosen for further sequencing and analyses (Fig. 2 and Table 1) yielded, on average, 49% endogenous DNA (data S1). We shotgun sequenced these individuals to an average genomic coverage of 0.019 to 1.95× (average, 0.5×) with 69 genomes reaching >0.3×, 35 of those >0.5× and 8 of those in turn >1× (Table 1 and data S1). The presented genome-wide data are derived from 1 Neolithic individual (UkrN; 7000 to 6000 BCE), 9 individuals from the Bronze Age and from the Final Bronze Age to the beginning of the Iron Age (UkrBA and UkrFBA/EIA; 3000 to 700 BCE), 6 individuals from the beginning of the Early Iron Age (UkrEIA; 900 to 700 BCE), 29 individuals from the Scythian period of the Early Iron Age (UkrEIA; 700 to 300 BCE), 6 individuals from the end of the Early Iron Age (UkrEIA; 400 to 1 BCE), 12 individuals from the later Iron Age (UkrIA; 1 to 400 CE), 9 individuals from the Early Middle Ages (UkrEMA; 800 to 900 CE), and 19 individuals from the Middle Ages to the early modern period (UkrMA and UkrEM; 900 to 1800 CE) (Fig. 2, data S1, and Supplementary Text). We analyzed the data in the context of published ancient and modern genomes, which have been assigned to groups based on ancestry, geography, time period, and archaeological culture, as defined in the Allen Ancient DNA Resource (AADR) (*42*) version 54.1 (data S3 and S4). Previously published genomes from individuals with the same archaeological associations (*7*) were analyzed and discussed alongside individuals sequenced in this study.

**Table 1.** Archaeological information, genetic sex, mtDNA, and Y chromosome haplogroups and average genomic coverage of the individuals of this study. Date (cal BCE/CE), calibrated using OxCal v4.4.4 and IntCal20 atmospheric curve (*104*, *105*); MT hg, mitochondrial DNA haplogroup; Y hg, Y chromosome haplogroup; Av. Cov., average genomic coverage.

Individual	Site	Region	Group	Date	Sex	MT hg	Y hg	Av. Cov.
UKR008	Mamay-Gora	Zaporizhzhia	UkrN_?	7000–6000 BCE	XY	U4c2	R1b-V88	0.087
UKR055	Sukha Gomilsha	Kharkiv	UkrBA_Zrubna	1873–1566 cal BCE	XX	H5a1	–	0.035
UKR170	Petrykiv	Ternopil	UkrFBA/EIA_Vysotska_Early	1300–800 BCE	XY	U2e2a1	R1a-M198	0.054
UKR171	Petrykiv	Ternopil	UkrFBA/EIA_Vysotska_Early	1278–1055 cal BCE	XY	U5a1a2b	R1a-M459	0.057
UKR149	Dykyi Sad	Mykolaiv	UkrFBA_Bilozerska_Pre-Cimmerian	1200–1000 BCE	XX	U4a1	–	1.310
UKR150	Dykyi Sad	Mykolaiv	UkrFBA_Bilozerska_Pre-Cimmerian	1200–1000 BCE	XY	N1a1a1a1	R1a-M198	0.096
UKR066	Kumy	Kharkiv	UkrFBA/EIA_Cimmerian	1195–919 cal BCE	XY	HV1a1a	Q1b-YP4000	0.451
UKR167	Rovantsi	Volyn	UkrFBA/EIA_Lusatian	1000–700 BCE	XX	J1c3g	–	1.950
UKR168	Rovantsi	Volyn	UkrFBA/EIA_Lusatian	1000–700 BCE	XX	K1a4a1e	–	0.852
UKR169	Rovantsi	Volyn	UkrFBA/EIA_Lusatian	1000–700 BCE	XY	H1an1	R1a-M459	0.085
UKR000	Kartal	Odesa	UkrEIA_ThracianHallstatt_2	900–798 cal BCE	XX	U4a2a	–	1.170
UKR001	Kartal	Odesa	UkrEIA_ThracianHallstatt	900–700 BCE	XX	K1a	–	1.020
UKR002	Kartal	Odesa	UkrEIA_ThracianHallstatt	900–700 BCE	XY	U5b1a	C1a-Z38888	0.422
UKR005	Kartal	Odesa	UkrEIA_ThracianHallstatt	900–700 BCE	XY	T2	?	0.026
UKR006	Kartal	Odesa	UkrEIA_ThracianHallstatt	900–700 BCE	XX	U5a1h	–	0.052
UKR007	Kartal	Odesa	UkrEIA_ThracianHallstatt	996–831 cal BCE	XY	T1a1	E1b-V13	0.313
UKR035AB	Medvyn	Kyiv	UkrEIA_Scythian_RightDnipro_IllThr	700–600 BCE	XY	U5a1g1	R1a-Z283	1.690
UKR036	Medvyn	Kyiv	UkrEIA_Scythian_RightDnipro_IllThr_2	773–426 cal BCE	XY	C4b	R1a-Z645	0.738
UKR039	Medvyn	Kyiv	UkrEIA_Scythian_RightDnipro_IllThr	700–600 BCE	XY	U5a1a1a	R1a-M420	0.060
UKR042	Medvyn	Kyiv	UkrEIA_Scythian_RightDnipro_IllThr	779–539 cal BCE	XX	T2a2	–	0.170
UKR043	Medvyn	Kyiv	UkrEIA_Scythian_RightDnipro_IllThr	700–600 BCE	XX	U5a2b	–	0.372
UKR044	Medvyn	Kyiv	UkrEIA_Scythian_RightDnipro_IllThr	700–600 BCE	XX	H6a1b	–	0.424
UKR078	Bilsk hillfort	Poltava	UkrEIA_Scythian_LeftDnipro_IllThr	755–408 cal BCE	XX	H6a1b	–	0.819
UKR083	Bilsk hillfort	Poltava	UkrEIA_Scythian_LeftDnipro_LocAgr	500–300 BCE	XY	U4d3	R1a-M198	0.080
UKR087	Bilsk hillfort	Poltava	UkrEIA_Scythian_LeftDnipro_LocEl	650–600 BCE	XX	H3v+	–	0.400
UKR088	Bilsk hillfort	Poltava	UkrEIA_Scythian_LeftDnipro_LocEl	761–420 cal BCE	XX	U2b	–	0.457
UKR089	Bilsk hillfort	Poltava	UkrEIA_Scythian_LeftDnipro_LocEl	500–300 BCE	XY	H+152	E1b-V13	0.449
UKR090	Bilsk hillfort	Poltava	UkrEIA_Scythian_LeftDnipro_LocEl	400–300 BCE	XY	H11a1	E1b-V13	0.450
UKR091	Bilsk hillfort	Poltava	UkrEIA_Scythian_LeftDnipro_LocEl	500–400 BCE	XY	H+152	E1b-V13	0.327
UKR095	Kolomak	Kharkiv	UkrEIA_Scythian_LeftDnipro_LocAgr	389–204 cal BCE	XY	J2b1a2a	R1a-Z93	0.699
UKR096	Kolomak	Kharkiv	UkrEIA_Scythian_LeftDnipro_LocAgr_2	382–199 cal BCE	XX	T2a1a	–	1.030
UKR105	Kupievakha	Kharkiv	UkrEIA_Scythian_SivDon_Nom	798–552 cal BCE	XY	J1d6	R1a-YP5018	0.644
UKR101	Nyzhnia Gyivka	Kharkiv	UkrEIA_Scythian_SivDon_Nom	400–300 BCE	XX	I1a1	–	0.375
UKR114	Nyzhnia Gyivka	Kharkiv	UkrEIA_Scythian_SivDon_Nom_2	500–400 BCE	XX	H4a1c	–	0.290
UKR104	Grishkivka	Kharkiv	UkrEIA_Scythian_SivDon_Nom	500–350 BCE	XY	H6a1b	R1a-Z645	0.919
UKR109	Vesele	Kharkiv	UkrEIA_Scythian_SivDon_Nom	400–300 BCE	XY	H5a1	Q1b-L330	0.530
UKR110	Vesele	Kharkiv	UkrEIA_Scythian_SivDon_Nom	400–300 BCE	XY	U3a1b	R1b-Z2105	0.430
UKR111	Cheremushna	Kharkiv	UkrEIA_Scythian_SivDon_Nom_2	775–540 cal BCE	XY	K2	R1a-YP5018	0.506
UKR113	Mala Rogozianka	Kharkiv	UkrEIA_Scythian_SivDon_Nom	650–550 BCE	XY	HV0a	Q1b-L330	0.716
UKR116	Karavan	Kharkiv	UkrEIA_Scythian_SivDon_NomEl_3	775–516 cal BCE	XY	U2e1f1	R1a-Z645	0.598
UKR131	Pisochyn	Kharkiv	UkrEIA_Scythian_SivDon_NomEl	500–300 BCE	XY	I1c1a	R1b-M269	0.484
UKR132	Pisochyn	Kharkiv	UkrEIA_Scythian_SivDon_NomEl	500–300 BCE	XY	X2f	R1b-L23	0.872
UKR133	Pisochyn	Kharkiv	UkrEIA_Scythian_SivDon_NomEl	500–300 BCE	XY	T1b	R1b-Z2105	0.925
UKR013	Mamay-Gora	Zaporizhzhia	UkrEIA_Scythian_NBlaSea_Nom	400–300 BCE	XX	HV2a3	–	0.398
UKR014	Mamay-Gora	Zaporizhzhia	UkrEIA_Scythian_NBlaSea_Nom	400–300 BCE	XY	I4a	R1a-Z645	0.290
UKR152	Oleksandrivskyi necropolis	Mykolaiv	UkrEIA_Antiquity_Greeks?_1	392–206 cal BCE	XY	HV1b	E1b-V13	0.818
UKR153	Oleksandrivskyi necropolis	Mykolaiv	UkrEIA_Antiquity_Greeks?_2	746–401 cal BCE	XY	W6b	R1a-M459	0.573
UKR174	Petrykiv	Ternopil	UkrEIA_?	359–104 cal BCE	XX	HV2	–	0.534
UKR051	Maslyny	Crimea	UkrEIA_LateScythian_Cri_Nom	150–1 BCE	XX	U7a3a*	–	0.648
UKR052	Maslyny	Crimea	UkrEIA_LateScythian_Cri_Nom	150–1 BCE	XY	R1a1a	R1b-M269	0.066
UKR053	Maslyny	Crimea	UkrEIA_LateScythian_Cri_Nom	150–1 BCE	XX	HV1a1	–	0.029
UKR160	Liubivka	Kharkiv	UkrIA_Sarmatian_SivDon	1–300 CE	XX	H16+	–	1.220
UKR045	Lehedzyne	Cherkasy	UkrIA_Chernyakhiv_2	300–400 CE	XX	U4c1	–	0.375
UKR047	Lehedzyne	Cherkasy	UkrIA_Chernyakhiv_2	300–400 CE	XX	H2a2a	–	0.138
UKR049	Komariv-1	Chernivtsi	UkrIA_Chernyakhiv_3	169–338 cal CE	XX	HV0a	–	0.433
UKR102	Zolochiv	Kharkiv	UkrIA_Chernyakhiv_2	300–400 CE	XY	H26	E1b-V13	0.755
UKR121	Shуshakу	Poltava	UkrIA_Chernyakhiv_2	300–400 CE	XX	H1	–	0.347
UKR122	Shуshakу	Poltava	UkrIA_Chernyakhiv_1	300–400 CE	XX	K1b2b	–	0.334
UKR123	Shуshakу	Poltava	UkrIA_Chernyakhiv_2	300–400 CE	XY	H7	R1a-Z93	0.681
UKR125	Shуshakу	Poltava	UkrIA_Chernyakhiv_1	300–400 CE	XX	K1a4a1b	–	0.309
UKR126	Shуshakу	Poltava	UkrIA_Chernyakhiv_1	131–325 cal CE	XY	H1a3	R1a-Z645	0.389
UKR128	Shуshakу	Poltava	UkrIA_Chernyakhiv_2	229–361 cal CE	XX	K1a+	–	0.390
UKR129	Shуshakу	Poltava	UkrIA_Chernyakhiv_1	245–401 cal CE	XX	K1c2	–	0.296
UKR134	Verkhnii Saltiv	Kharkiv	UkrEMA_Saltiv_Alans_2	800–900 CE	XX	A16	–	0.879
UKR135	Verkhnii Saltiv	Kharkiv	UkrEMA_Saltiv_Alans_1	800–900 CE	XY	A+	R1b-L23	0.422
UKR136	Verkhnii Saltiv	Kharkiv	UkrEMA_Saltiv_Alans_1	800–900 CE	XY	W3	R1b-M269	0.445
UKR137	Verkhnii Saltiv	Kharkiv	UkrEMA_Saltiv_Alans_2	800–900 CE	XY	F2c1	C2a-Y11606	0.359
UKR138	Verkhnii Saltiv	Kharkiv	UkrEMA_Saltiv_Alans_1	800–900 CE	XY	H27+	R1b-Z2105	0.441
UKR139	Verkhnii Saltiv	Kharkiv	UkrEMA_Saltiv_Alans_1	671–874 cal CE	XX	W6	–	0.343
UKR143	Bochkove	Kharkiv	UkrEMA_Saltiv_Bulgars_1	800–900 CE	XX	U5a1a1	–	0.569
UKR144	Bochkove	Kharkiv	UkrEMA_Saltiv_Bulgars_2	671–874 cal CE	XX	B4c1a2a	–	0.390
UKR147	Bochkove	Kharkiv	UkrEMA_Saltiv_Bulgars_1	683–883 cal CE	XY	D4	R1a-M417	0.152
UKR012	Mamay-Gora	Zaporizhzhia	UkrMA_Cuman	1100-1430 CE	XY	J1b1b1	R1a-Z645	0.374
UKR027	Velyka Znamianka	Zaporizhzhia	UkrMA_Cuman	1100-1200 CE	XY	H5a1	N1a-Y10755	0.317
UKR056	Kumy	Kharkiv	UkrMA_Cuman_2	991–1149 cal CE	XX	B5b2a2	–	0.832
UKR166	Zvenigorod	Lviv	UkrMA_WhiteCroat_Slavs	1100–1300 CE	XX	H1a	–	1.040
UKR068	Donets hillfort	Kharkiv	UkrMA_GoldenHorde_Slav/Nom?	1200–1400 CE	XX	U5b1e1a	–	0.784
UKR069	Donets hillfort	Kharkiv	UkrMA_GoldenHorde_Slav/Nom?	1200–1400 CE	XY	X2d1a	R1b-Z2105	0.531
UKR070	Donets hillfort	Kharkiv	UkrMA_GoldenHorde_Slav/Nom?	1200–1400 CE	XY	U5a1a1a	?	0.019
UKR063	Kumy	Kharkiv	UkrMA_GoldenHorde_Nom	1300–1400 CE	XX	U2e1b	–	0.285
UKR074	Ploske	Donetsk	UkrMA_GoldenHorde_Nom	1200–1400 CE	XX	A1a	–	0.333
UKR028	Mamay-Surka	Zaporizhzhia	UkrMA_Post-Cuman_Cuman?	1300–1400 CE	XX	J1c2e1	–	0.394
UKR016	Mamay-Gora	Zaporizhzhia	UkrMA_Nogai_1	1400–1500 CE	XY	M7c1a1a1	R1b-Y14051	0.322
UKR017	Mamay-Gora	Zaporizhzhia	UkrMA_Nogai_2	1400–1500 CE	XY	C4a1a4a	C2a-M504	0.764
UKR018	Mamay-Gora	Zaporizhzhia	UkrMA_Nogai_3	1400–1500 CE	XX	B4d1'2'3	–	0.260
UKR020	Mamay-Gora	Zaporizhzhia	UkrMA_Nogai_2	1400–1500 CE	XY	M7b1a1a1	J1a-Y5321	0.607
UKR021	Mamay-Gora	Zaporizhzhia	UkrMA_Nogai_3	1400–1500 CE	XX	C4a1a4a	–	0.633
UKR022	Mamay-Gora	Zaporizhzhia	UkrMA_Nogai_2	1400–1500 CE	XX	U2e2a1a2	–	0.387
UKR024	Mamay-Gora	Zaporizhzhia	UkrMA_Nogai_1	1400–1500 CE	XY	M65a+	G2a-Z6679	0.523
UKR164	Vypovziv	Chernigiv	UkrEM_Cossack_Slavs	1600–1800 CE	XX	H3a1	–	0.444
UKR165	Vypovziv	Chernigiv	UkrEM_Cossack_Slavs	1600–1800 CE	XX	U4a2b	–	0.272

### Southern European ancestry in Ukraine in the Late Bronze Age to pre-Scythian Iron Age period

The mitochondrial DNA (mtDNA) of the Late Bronze Age and pre-Scythian Early Iron Age (LBAEIA; 3000 to 700 BCE) individuals belonged to haplogroups (hgs) U, HV, H, T, K, J, and N1a (Table 1 and data S1), while the Y chromosomes (chrY) of most males belonged to hg R1a (Table 1 and data S1 and S2), as has been shown previously for much of northern Europe after the steppe migrations (*1*, *2*). However, the Cimmerian male belonged to chrY hg Q1b-YP4004 [common in Central Asia and Siberia (*43*) but not in Europe (*44*)], and one Thracian Hallstatt individual belonged to C1-Y83490 [sub-branches of C-V20 are found in ancient Europeans in the Stone Age (*45*–*47*) but rare in Europe today (*48*)] (Table 1 and data S1 and S2).

**Fig. 3.**
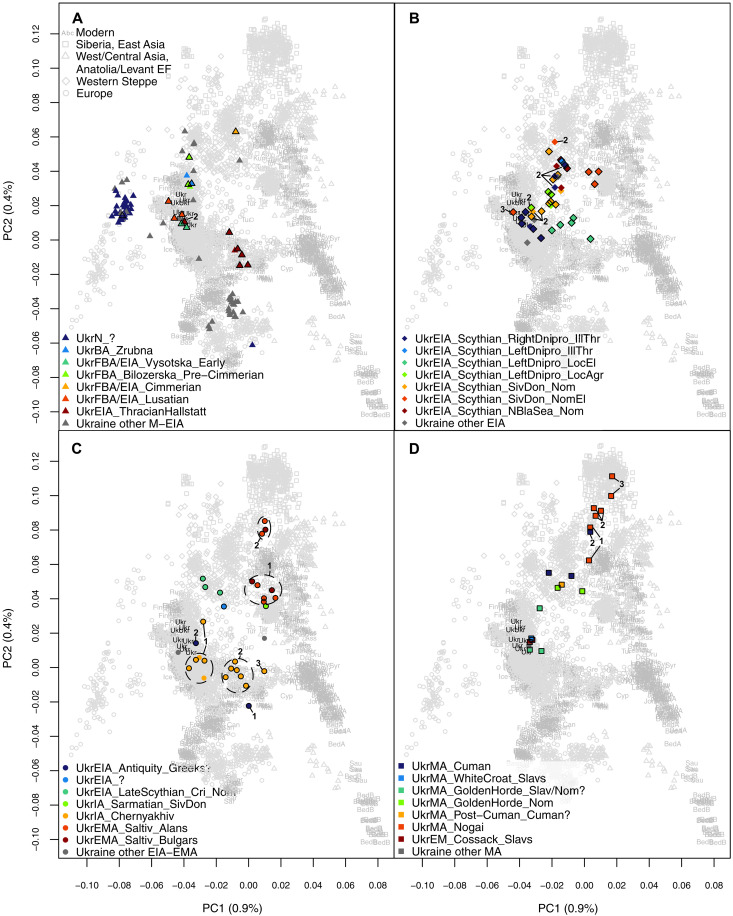
PCA results. PCA results of modern West Eurasians with ancient individuals projected onto the first two components (PC1 and PC2). Ukrainian groups from (**A**) Late Bronze Age and pre-Scythian Iron Age (3000 to 700 BCE), (**B**) the Scythian period of Early Iron Age (700 to 300 BCE), (**C**) post-Scythian Iron Age until Early Middle Ages (400 BCE to 900 CE), and (**D**) Middle Ages and early modern period (MAEM; 900 to 1800 CE). Newly reported individuals are indicated with a black outline. Genetic subgroups used in subsequent analyses are indicated with black numbers. Modern Ukrainians are shown in black.

**Fig. 4.**
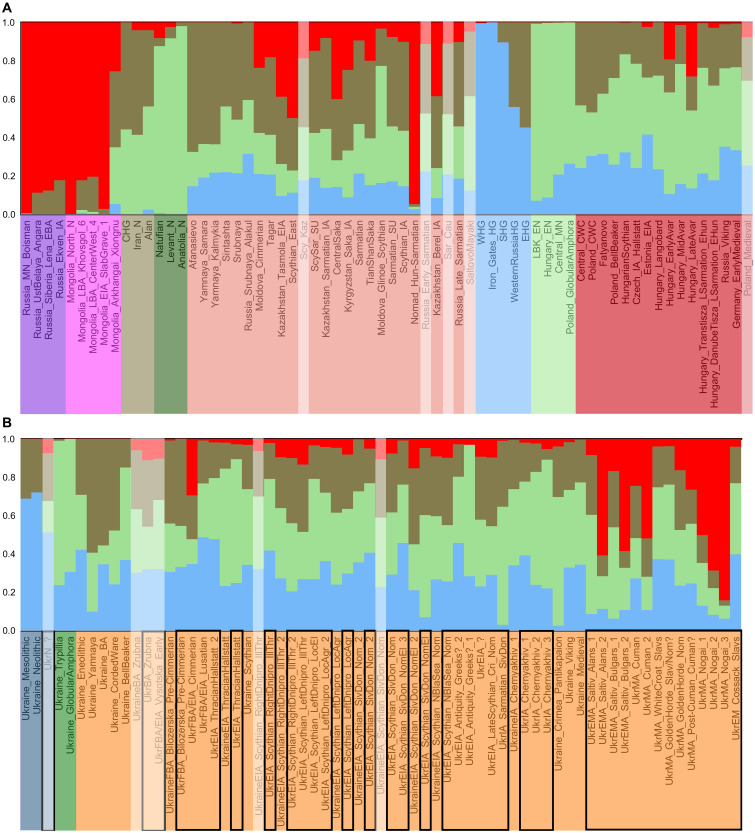
Subset of Admixture analysis results with genetic structure calculated on ancient individuals. Population averages of (**A**) Eurasia-wide, (**B**) Ukrainian ancient groups at *K* = 4. Purple, Siberia; magenta, East Asia; olive, West/Central Asia; dark green, Anatolian/Levantine early farmers; salmon, Western Steppe; light blue, European HGs; light green, European early farmers; dark red, European post-steppe migration groups; dark blue, Ukrainian HGs; bright green, Ukrainian early farmers; orange, Ukrainian post-steppe migration groups.

We performed principal components analysis (PCA) (Fig. 3 and figs. S1 and S2) and Admixture analysis (Fig. 4 and figs. S3 and S4) using autosomal data of modern individuals (“modern” Admixture) (data S3) and projecting ancient individuals (data S4) onto the components. Admixture analysis was also performed without modern individuals, using only ancient individuals with up to 90% missing positions after pruning the dataset to decrease linkage disequilibrium (maximum of five individuals per group) (Materials and Methods and data S4) and later also projecting all ancient individuals onto the components (“ancient” Admixture). The Zrubna individual of this study (1873 to 1566 cal BCE) and the previously published Zrubna individual from Ukraine (781 to 511 cal BCE) (*7*) (UkrBA_Zrubna) cluster on PCA with individuals from present-day Russia associated with the same culture (Fig. 3A and figs. S1 and S2A). In both PCA and Admixture, Zrubna individuals are similar to Yamna individuals, but some Zrubna genomes show signs of Admixture with early farmers (Fig. 3A and figs. S1, S2A, S3, and S4). Bilozerska Pre-Cimmerians [archaeologically dated to 1200 to 1000 BCE, one radiocarbon dated to 1281 to 1058 cal BCE (*7*); UkrFBA_Bilozerska_Pre-Cimmerian] appear genetically similar to Zrubna individuals (Fig. 3A and figs. S1, S3, and S4CF). Early Vysotska (1300 to 800 BCE, one 1278 to 1055 cal BCE; UkrFBA/EIA_Vysotska_Early) and Lusatian (1000 to 700 BCE; UkrFBA/EIA_Lusatian) individuals appear similar to Northern and Eastern European individuals from the Iron Age to modern times (including modern Ukrainians) (Figs. 3A and 4 and figs. S1, S3, and S4). The Cimmerian individual (1195 to 919 cal BCE; UkrFBA/EIA_Cimmerian) clusters on the PCA plot with Western Steppe individuals (Fig. 3A and fig. S1), including previously published Cimmerians from Moldova (fig. S2A), but with a bigger East Asian genetic influence compared to Bilozerska Pre-Cimmerians (Fig. 4 and figs. S3 and S4). Most of the Early Iron Age Thracian Hallstatt individuals (900 to 700 BCE, two 996 to 830 cal BCE; UkrEIA_ThracianHallstatt) cluster with Southern Europeans on the PCA plot (Fig. 3A and fig. S1), while previously published Hallstatt individuals from the Czech Republic cluster with Central European individuals (fig. S2A). A bigger early farmer influence compared to earlier individuals from Ukraine can also be seen on Admixture (Fig. 4 and figs. S3 and S4). One Thracian Hallstatt individual (900 to 798 cal BCE; UkrEIA_ThracianHallstatt_2) is similar to Early Vysotska and Lusatian individuals in both analyses (Figs. 3A and 4 and figs. S1, S3, and S4).

Then, we tested the cladality of the groups assigned based on archaeological context or identified in PCA and Admixture by calculating f4 statistics of the from f4 (Mbuti, ancient group; Ukrainian group 1, Ukrainian group 2) with a wide set of ancient groups (data S4) and considering the Ukrainian groups cladal if at least 95% of the f4 results were not considerably different from 0 (−3 ≤ |*Z*| ≤ 3) (data S5 and S6). The cladality of Zrubna (UkrBA_Zrubna) and Bilozerska (UkrFBA_Bilozerska_Pre-Cimmerian) individuals is confirmed, but Early Vysotska (UkrFBA/EIA_Vysotska_Early) and Lusatian (UkrFBA/EIA_Lusatian) individuals are noncladal with Lusatians having an additional affinity with some Siberian, East/West/Central Asian, Western Steppe, and post-steppe migration European groups (but not with any European early farmers) (data S5 and S6). The Thracian Hallstatt “outlier” (UkrEIA_ThracianHallstatt_2) is cladal with contemporary Lusatians (data S5 and S6). Furthermore, we tested the cladality of the groups of this study with previously published groups that are associated with the same cultures and saw that Ukrainian and Russian [Russia_Srubnaya_Alakul (*16*)] Zrubna individuals are cladal as is the Thracian Hallstatt outlier with a Czech Hallstatt outlier individual [Czech_IA_Hallstatt_2 (*49*)], but Ukrainian Cimmerian individuals (UkrFBA/EIA_Cimmerian) are noncladal with Moldovan ones [Moldova_Cimmerian (*16*)], the latter having relatively more affinity with some Siberian and East Asian groups (data S5 and S6).

**Fig. 5.**
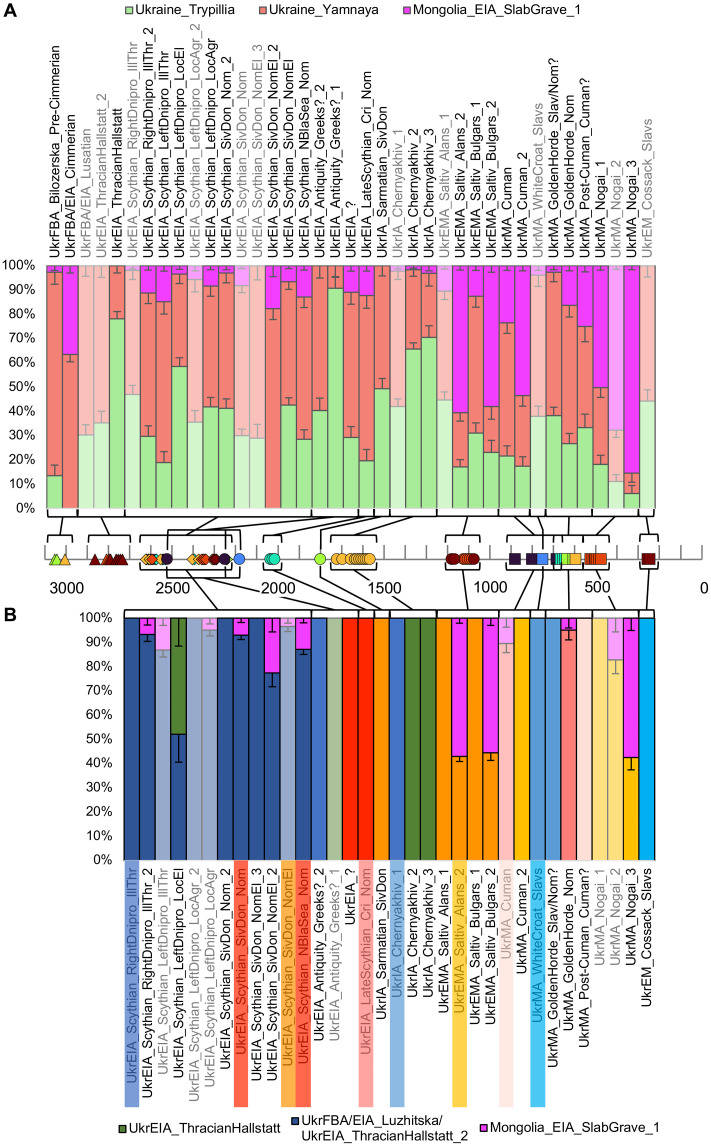
qpAdm admixture modeling results. (**A**) Distal qpAdm models of admixture between Ukraine_Trypillia, Ukraine_Yamnaya, and Mongolia_SlabGrave and (**B**) proximal qpAdm models, tested using the autosomal positions of the 1240K dataset. Rejected models (*P* < 0.05) are semitransparent. The dates used on the timeline are midpoints of the 95% calibrated date estimates or archaeological date range estimates with jitter.

We used qpAdm to model the ancestry proportions of the archaeological/genetic groups (Fig. 5, fig. S5, and data S7 to S11). First, we tested different combinations of an early farmer group (five total), a Yamna-associated group (three total), and an East Asian (Mongolian) group (three total) to find the distal sources that can be used to model the highest number of the groups of this study (the model is not rejected; *P* > 0.05) (data S7). The model including Middle Neolithic individuals from Germany [Central_MN (*8*)], Yamna-associated individuals from Ukraine [Ukraine_Yamnaya (*6*)], and Slab-grave culture–associated individuals from Mongolia [Mongolia_EIA_SlabGrave_1 (*50*), Mongolia_SlabGrave used for brevity] as sources is among the models with the highest number of nonrejected plausible results (27 of 39 modeled groups) (data S7). The best performing model with two Ukrainian sources includes Trypillia culture–associated individuals from Ukraine [Ukraine_Trypillia (*6*, *35*)], Ukraine_Yamnaya and Mongolia_SlabGrave, and gives nonrejected plausible results for 26 of 39 groups (data S7). When including models where one or two of the three available sources (Central_MN/Ukraine_Trypillia, Ukraine_Yamnaya, and Mongolia_SlabGrave) are dropped, nonrejected plausible results are produced for 28 of the 39 groups (Fig. 5A, fig. S5A, and data S8 and S9). Four groups of this study (UkrFBA/EIA_Lusatian, UkrEIA_Scythian_LeftDnipro_LocAgr_2, UkrMA_WhiteCroat_Slavs, and UkrMA_Nogai_2) do not produce a nonrejected result with any of the tested three-source models (data S7). All other groups, except for UkrEMA_Saltiv_Alans_1, give a nonrejected result when modeled from Mongolia_SlabGrave, Ukraine_Yamnaya, and Central_MN/Poland_GlobularAmphora/Ukraine_GlobularAmphora/Ukraine_Trypillia (*6*, *8*, *35*, *51*) with the point estimate of the early farmer ancestry proportion differing by a maximum of 7% between the highest *P* value model and the model with Ukraine_Trypillia (data S7), but even in this case, the one SE ranges of proportions overlap by 2%. Because of the small variability in the proportions, we use the model with Ukraine_Trypillia to describe all groups (Fig. 5A and data S7) and provide the model with Central_MN as comparison (fig. S5A and data S9). Furthermore, we show the highest *P* value three-way model out of all 315 tested models for each group (fig. S5B and data S10). None of the Zrubna (UkrBA_Zrubna) or Early Vysotska (UkrFBA/EIA_Vysotska_Early) individuals have enough data [>100,000 single-nucleotide polymorphisms (SNPs)] to be modeled, but the Bilozerska group (UkrFBA_Bilozerska_Pre-Cimmerian) can be modeled as mostly Ukraine_Yamnaya, some Ukraine_Trypillia, and a small amount of Mongolia_SlabGrave (84 ± 5% + 14 ± 4% + 3 ± 2%), and the Cimmerian individual (UkrFBA/EIA_Cimmerian) can be modeled as mostly Ukraine_Yamnaya and some Mongolia_SlabGrave [64 ± 3% + not applicable (NA) + 37 ± 3%] (Fig. 5A and data S8). The Thracian Hallstatt main group (UkrEIA_ThracianHallstatt) can be put together from some Ukraine_Yamnaya and mostly Ukraine_Trypillia, while the outlier individual (UkrEIA_ThracianHallstatt_2) the other way around (22 ± 3% + 78 ± 3% + NA and 65 ± 5% + 35 ± 5% + NA, respectively) (Fig. 5A and data S8).

To assess whether there is a sex bias in the ancestry distribution of any groups in this study, we calculated outgroup f3 statistics with a wide set of ancient groups (data S4) on both autosomal (data S12) and chrX (data S13) data. Autosomal f3 statistics reflect ancestry contributions approximately equally from both sexes, while chrX f3 statistics reflect approximately two-third female and one-third male ancestry contributions. We compared the results from both datasets to identify any differences in ancestry patterns, which may indicate sex-biased gene flow. The Zrubna-associated group shows a trend of higher similarity to Levantine/Anatolian and European early farmers on chrX and to Neolithic to Early Bronze Age Western Steppe groups and European HGs on autosomes (fig. S6A), consistent with admixture after the Yamna migration into Europe taking place mostly between males from the steppe (with a large proportion of Eastern HG ancestry) and local early farmer females, as has been inferred for Corded Ware Culture groups (*52*–*55*).

### Eastern European and Western Steppe ancestries in the Scythian period of Early Iron Age

The mtDNA variation during the Scythian period [Scythian period Early Iron Age (SEIA); 700 to 300 CE] is similar to the earlier time window but additionally includes mtDNA hgs I and X, and one individual carries hg C4 (Table 1 and data S1), which today has the highest frequency in Asia (*56*). The chrY lineages include R1a, R1b, E1b, and Central/East Asian (*43*) Q1b-L330 (Table 1 and data S1 and S2).

Kinship analysis done with Relationship Estimation from Ancient DNA (READ) (*57*) and KIN (*58*) indicates some close genetic relationships among the Scythian period individuals in this study and previously published individuals from the same sites from Järve *et al.* (*7*). Burial 1 in kurgan 22 in Medvyn in the Kyiv region includes two genetically identical samples (UKR035 and UKR038) that most likely come from the same individual (male, mtDNA hg U5a1g1, chrY hg R1a) (fig. S7A, data S14, and Supplementary Text) and were later merged together for further analyses (UKR035AB). However, the burial also includes another male (MJ-14; mtDNA hg H6a1b, chrY hg R1a) who has a parent-child relationship with the previously described male and also with a female individual (UKR044; mtDNA hg H6a1b), while the latter two are not closely related (fig. S7A and data S14). This means that MJ-14 is likely the son of UKR035AB and UKR044 (fig. S7A and data S14). Furthermore, there is another pair of parent-child relatives from the same site—two males, MJ-33 (mtDNA hg U5a2a2a, chrY hg R1a) and UKR036 (mtDNA hg C4b, chrY hg R1a), who could be father and son in either direction (fig. S7A and data S14). At the Bilsk hillfort in the Poltava region, the analysis identified another pair of genetically identical samples (UKR089 and UKR091; mtDNA hg H+152, chrY hg E1b), but these come from different kurgans that were excavated in different years, so it is likely that they were identical twins (fig. S7B, data S14, and Supplementary Text). These inferred twins are parent-child related to another male (UKR090; mtDNA hg H11a1, chrY hg E1b) who is likely their father (fig. S7B and data S14).

Scythian period individuals (nine of whom are radiocarbon dated to 798 to 199 cal BCE) were divided into groups based on their geographic location: right (i.e., west) or left (i.e., east) bank of Dnipro and Siversky Donets basin in the forest-steppe, Northern Black Sea region in the steppe. The groups were further divided on the basis of the sociocultural association inferred from archaeological context: Illirian-Thracian, local or nomad, agriculturalist or elite. The groups are named using the following structure, separated by underscores: time period, cultural association, geographic location, sociocultural association, and genetic subgroup (where relevant) (Fig. 2, Table 1, data S1, and Supplementary Text). Most of the Scythian individuals from the right bank of Dnipro with Illirian-Thracian associations (UkrEIA_Scythian_RightDnipro_IllThr), one individual from local agricultural tribes of the left bank of Dnipro (UkrEIA_Scythian_LeftDnipro_LocAgr_2), some of the non-elite nomad individuals from Siversky Donets basin (UkrEIA_Scythian_SivDon_Nom_2), and one elite nomad individual from the same area (UkrEIA_Scythian_SivDon_NomEl_3) are similar to previous Early Vysotska and Lusatian individuals and also modern Ukrainians in both PCA and Admixture (Figs. 3B and 4 and figs. S1, S2B, S3, and S4). The rest of the Scythian individuals from the right bank of Dnipro with Illirian-Thracian associations (UkrEIA_Scythian_RightDnipro_IllThr_2) and from local agricultural groups of the left bank of Dnipro (UkrEIA_Scythian_LeftDnipro_LocAgr) are more similar to Western Steppe individuals (including previously published Scythian-related individuals from the region) (Figs. 3B and 4 and figs. S1, S2B, S3, and S4). The same is true for most of the non-elite nomad individuals from Siversky Donets basin (UkrEIA_Scythian_SivDon_Nom) and one elite nomad individual from the same region (UkrEIA_Scythian_SivDon_NomEl_2), the individual from the left bank of Dnipro with Illirian-Thracian associations (UkrEIA_Scythian_LeftDnipro_IllThr), as well as the four steppe nomads from the Northern Black Sea region (UkrEIA_Scythian_NBlaSea_Nom) (Figs. 3B and 4 and figs. S1, S2B, S3, and S4). Local elite individuals from the left bank of Dnipro (UkrEIA_Scythian_LeftDnipro_LocEl) have greater genetic affinity to Southern European individuals (somewhat similarly to Scythians from Moldova) (Figs. 3B and 4 and figs. S1, S2B, S3, and S4). Most of the elite nomads from the Siversky Donets basin (UkrEIA_Scythian_SivDon_NomEl) (*n* = 3) share highest similarity with individuals from the Caucasus (Figs. 3B and 4 and figs. S1, S3, and S4).

The f4-based cladality test supports the main group of Illirian-Thracian–associated Scythians from the right bank of Dnipro (UkrEIA_Scythian_RightDnipro_IllThr) being cladal with two of the groups it clusters with in PCA (UkrEIA_Scythian_SivDon_Nom_2, UkrEIA_Scythian_LeftDnipro_LocAgr_2), but not with the elite nomad individual from Siversky Donets (UkrEIA_Scythian_SivDon_NomEl_3), who has relatively more affinity with some East Asian groups and European HGs (data S5 and S6). Also, the main group of non-elite Siversky Donets nomads (UkrEIA_Scythian_SivDon_Nom) is cladal with three of the groups it clusters with in PCA (UkrEIA_Scythian_RightDnipro_IllThr_2, UkrEIA_Scythian_LeftDnipro_LocAgr, and UkrEIA_Scythian_NBlaSea_Nom) but shares more with several European groups compared to the Illirian-Thracian–associated Scythian from the right bank of Dnipro (UkrEIA_Scythian_LeftDnipro_IllThr) and less with some Siberian and East Asian groups than the elite nomad individual from Siversky Donets (UkrEIA_Scythian_SivDon_NomEl_2). The main group of the Illirian-Thracian–associated Scythians from the right bank of Dnipro (UkrEIA_Scythian_RightDnipro_IllThr) are cladal with the preceding Thracian Hallstatt outlier and Early Vysotska individuals (UkrEIA_ThracianHallstatt_2 and UkrFBA/EIA_Vysotska_Early, respectively), as well as the main subgroup of Hungarian Scythians [HungarianScythian_1 (*17*)] (data S5 and S6). Furthermore, the main group of Siversky Donets elite nomads (UkrEIA_Scythian_SivDon_NomEl) is cladal with one Scythian individual from Kazakhstan [Scy_Kaz_2 (*7*)] (data S5 and S6).

With distal qpAdm modeling, the main group of the Illirian-Thracian–associated Scythians from the right bank of Dnipro and the subset of non-elite nomads from Siversky Donets (UkrEIA_Scythian_RightDnipro_IllThr and UkrEIA_Scythian_SivDon_Nom_2, respectively) can be modeled as approximately half Ukraine_Yamnaya and half Ukraine_Trypillia, with a small amount of Mongolia_SlabGrave ancestry (50 to 58% + 40 to 48% + 1 to 4% on average) (Fig. 5A and data S8). The main group of Siversky Donets elite nomads (UkrEIA_Scythian_SivDon_NomEl) has slightly more Mongolia_SlabGrave ancestry (51 ± 3% + 43 ± 3% + 7 ± 1%), while the “European outlier” of the group (UkrEIA_Scythian_SivDon_NomEl_3) can be modeled without it (71 ± 6% + 29 ± 6% + NA) (Fig. 5A and data S8). Most groups in the so-called Western Steppe cluster in PCA (UkrEIA_Scythian_RightDnipro_IllThr_2, UkrEIA_Scythian_LeftDnipro_LocAgr, and UkrEIA_Scythian_SivDon_Nom, UkrEIA_Scythian_NBlaSea_Nom) can be put together from mostly Ukraine_Yamnaya, some Ukraine_Trypillia, and some additional Mongolia_SlabGrave ancestry (53 to 61% + 29 to 36% + 9 to 12% on average), while the Illirian-Thracian–associated individual from the right bank of Dnipro (UkrEIA_Scythian_LeftDnipro_IllThr) has slightly less of Ukraine_Trypillia ancestry and more of the other two (66 ± 5% + 19 ± 5% + 15 ± 2%) (Fig. 5A and data S8). The “Steppe outlier” of Siversky Donets elite nomads (UkrEIA_Scythian_SivDon_NomEl_2), however, can be modeled without Ukraine_Trypillia ancestry (82 ± 5% + NA + 18 ± 5%), while the local elite individuals from the left bank of Dnipro (UkrEIA_Scythian_LeftDnipro_LocEl) need more than other Scythian groups (38 ± 4% + 58 ± 4% + 3 ± 1%) (Fig. 5A and data S8).

Next, we also modeled the groups of this study starting from the Scythian period using relevant groups from earlier periods as sources (proximal qpAdm modeling) (data S11). The analysis showed that groups from the “European” cluster in PCA (UkrEIA_Scythian_RightDnipro_IllThr, UkrEIA_Scythian_SivDon_Nom_2, and UkrEIA_Scythian_SivDon_NomEl_3) can be modeled as 100% UkrFBA/EIA_Lusatian or UkrEIA_ThracianHallstatt_2 ancestry, while groups from the “Western Steppe” cluster in PCA (UkrEIA_Scythian_RightDnipro_IllThr_2, UkrEIA_Scythian_SivDon_Nom, UkrEIA_Scythian_SivDon_NomEl_2, and UkrEIA_Scythian_NBlaSea_Nom) show an additional input from Mongolia_SlabGrave (7 ± 3%, 7 ± 2%, 23 ± 6%, and 13 ± 2%, respectively) (Fig. 5B and data S11). The local elite individuals from the left bank of Dnipro (UkrEIA_Scythian_LeftDnipro_LocEl) can be modeled as approximately half UkrEIA_ThracianHallstatt_2 and half UkrEIA_ThracianHallstatt (52 ± 11% + 48 ± 11%) ancestry (Fig. 5B and data S11).

### Appearance of ancestry profiles from Southern Europe, the Caucasus, and Central Asia during post-Scythian Iron Age (Hellenistic period) until Early Middle Ages

The mtDNA lineages carried by individuals from post-Scythian Iron Age (Hellenistic period) and early medieval period [post-Scythian Iron Age to Early Middle Ages (IAEMA); 400 BCE to 900 CE] in Ukraine include many of those already seen in earlier periods, as well as mtDNA hg W and one case of R1a1a (Table 1 and data S1), which is found in the Caucasus (*59*) but very rarely observed in modern-day Eastern Europe (*60*). Furthermore, Saltiv culture–associated individuals carry lineages belonging, among others, to mtDNA hgs A, B, D, and F (Table 1 and data S1), all frequent in East Asian populations today and rare in Europe (*61*–*64*). The chrY lineages present in the individuals from this period are again R1a, R1b, and E1b as well as Central East Asian (*65*) C2a-Y11606 in one Saltiv-associated individual (Table 1 and data S1 and S2).

The dataset includes two individuals that have been tentatively associated with Greeks of the Hellenistic period (Table 1 and data S1). One of the individuals (392 to 206 cal BCE; UkrEIA_Antiquity_Greeks?_1) has the highest affinity with Southern Europeans, but the other (746 to 401 cal BCE; UkrEIA_Antiquity_Greeks?_2) is most similar to Northern and Eastern European individuals (including modern Ukrainians) (Figs. 3C and 4 and figs. S1, S2C, S3, and S4). An individual with no specific archaeological association (359 to 104 cal BCE; UkrEIA_?) and all three available Late Scythians (150 to 1 BCE; UkrEIA_LateScythian_Cri_Nom) have the highest affinity with Western Steppe individuals, including some of the Scythian period individuals of this study (see the previous subsection) (Figs. 3C and 4 and figs. S1, S3, and S4). The Chernyakhiv culture–associated individuals (300 to 400 CE, seven 131 to 530 cal CE) can be divided into three genetic subgroups—six individuals are most similar to Eastern/Central Europeans (UkrIA_Chernyakhiv_1), six to continental Southern Europeans (clustering with Thracian Hallstatt–associated individuals of this study; fig. S1) (UkrIA_Chernyakhiv_2), and one individual has an even more southern genetic profile, clustering with modern Cypriots (UkrIA_Chernyakhiv_3) (Figs. 3C and 4 and figs. S1, S3, and S4). Most of the individuals are from one site (Shyshaky, Poltava region) but are divided between the first and second genetic subgroups (Table 1 and data S1). The one available Sarmatian (1 to 300 CE; UkrIA_Sarmatian_SivDon) and most of the Saltiv culture–associated Alans and Bulgars (800 to 900 CE, two 671 to 883 cal CE; UkrEMA_Saltiv_Alans/Bulgars_1) have the highest affinity with individuals from the Caucasus, similarly to previously published Saltiv Culture individuals and Alans, and most of the Siversky Donets basin elite nomads from this study, but not with previously published Sarmatians (Figs. 3C and 4 and figs. S1, S2C, S3, and S4). Three of the Saltiv culture–associated Alans and Bulgars from the same sites as the previously mentioned individuals (800 to 900 CE, one 671 to 874 cal CE; UkrEMA_Saltiv_Alans/Bulgars_2) are most similar to Central Asians, unlike any previously published Saltiv- or Alan-associated individual (Figs. 3C and 4 and figs. S1, S2C, S3, and S4).

The f4-based cladality tests confirm that Alans and Bulgars are cladal with each other within genetic subgroups 1 and 2 (UkrEMA_Saltiv_Alans_1 with UkrEMA_Saltiv_Bulgars_1and UkrEMA_Saltiv_Alans_2 with UkrEMA_Saltiv_Bulgars_2, respectively) (data S5 and S6). Furthermore, cladality is confirmed within the “Eastern/Central European” (UkrEIA_Antiquity_Greeks?_2, UkrIA_Chernyakhiv_1) and “Western Steppe” (UkrEIA_?, UkrEIA_LateScythian_Cri_Nom) PCA clusters, but the Siversky Donets Sarmatian (UkrIA_Sarmatian_SivDon) is noncladal with the Caucasus-like Alans (UkrEMA_Saltiv_Alans_1), the latter sharing more ancestry with some Siberian and East Asian groups (data S5 and S6). The Eastern/Central European Chernyakhiv group (UkrIA_Chernyakhiv_1) is cladal with the preceding main group of the Illirian-Thracian–associated Scythians from the right bank of Dnipro (UkrEIA_Scythian_RightDnipro_IllThr), as are the Late Scythian nomads from Crimea (UkrEIA_LateScythian_Cri_Nom) with preceding Scythian non-elite nomads from Siversky Donets (UkrEIA_Scythian_SivDon_Nom) and the Caucasus-like Alans (UkrEMA_Saltiv_Alans_1) with the preceding main group of Scythian elite nomads from Siversky Donets (UkrEIA_Scythian_SivDon_NomEl) (data S5 and S6). The aforementioned Alan group (UkrEMA_Saltiv_Alans_1) is cladal with Saltovo Mayaki individuals from Russian Caspian Steppe [SaltovoMayaki (*17*)] but not with Alans from Russian Caucasus [Alan (*17*)], the latter having more affinity with West/Central Asian, Western Steppe, and European groups (data S5 and S6).

Distal qpAdm modeling indicates that one of the potentially Greek individuals (UkrEIA_Antiquity_Greeks?_1) can be modeled as a small amount of Ukraine_Yamnaya and otherwise almost entirely Ukraine_Trypillia (9 ± 4% + 91 ± 4% + NA), while the other potentially Greek individual (UkrEIA_Antiquity_Greeks?_2) and the Sarmatian individual from Siversky Donets (UkrIA_Sarmatian_SivDon) model as near equal proportions of the same sources (60 ± 5% + 40 ± 5% + NA and 51 ± 4% + 49 ± 4% + NA, respectively) (Fig. 5A and data S8). The “Eastern/Central European” Chernyakhiv group (UkrIA_Chernyakhiv_1) can be put together from mostly Ukraine_Yamnaya, slightly less of Ukraine_Trypillia, and a small amount of Mongolia_SlabGrave ancestry (56 ± 3% + 42 ± 3% + 2 ± 1%), while the other two subgroups (UkrIA_Chernyakhiv_2 and UkrIA_Chernyakhiv_3) have more Ukraine_Trypillia ancestry (33 ± 3% + 66 ± 3% + 2 ± 1% and 26 ± 5% + 71 ± 5% + 3 ± 2%, respectively) (Fig. 5A and data S8). The “Western Steppe/Caucasus” cluster in PCA (UkrEIA_?, UkrEIA_LateScythian_Cri_Nom, UkrEMA_Saltiv_Bulgars_1) can be explained by combining ancestry from mostly Ukraine_Yamnaya, some Ukraine_Trypillia, and slightly less of Mongolia_SlabGrave (52 to 73% + 15 to 35% + 9 to 15% overall) (Fig. 5A and data S8). On the other hand, the Central Asian–like Alans and Bulgars (UkrEMA_Saltiv_Alans_2 and UkrEMA_Saltiv_Bulgars_2) can be modeled as around equal proportions of Ukraine_Yamnaya and Ukraine_Trypillia but mostly Mongolia_SlabGrave ancestry (16 to 25% + 16 to 24% + 57 to 61% on average) (Fig. 5A and data S8).

Using proximal qpAdm modeling, the “Eastern/Central European” PCA cluster (UkrEIA_Antiquity_Greeks?_2, UkrIA_Chernyakhiv_1) can be modeled as 100% UkrEIA_Scythian_RightDnipro_IllThr while the “Western Steppe” one (UkrEIA_?, UkrEIA_LateScythian_Cri_Nom) as 100% UkrEIA_Scythian_SivDon_Nom or UkrEIA_Scythian_NBlaSea_Nom (Fig. 5B and data S11). The “Caucasus” cluster (UkrIA_Sarmatian_SivDon, UkrEMA_Saltiv_Alans_1, and UkrEMA_Saltiv_Bulgars_1) can be put together from 100% UkrEIA_Scythian_SivDon_NomEl, but the “Central Asia” cluster (UkrEMA_Saltiv_Alans_2 and UkrEMA_Saltiv_Bulgars_2) also shows 54 to 59% Mongolia_SlabGrave on average (Fig. 5B and data S11). The Southern European–like Chernyakhiv individuals (Chernyakhiv_2 and Chernyakhiv_3) can be modeled as 100% UkrEIA_ThracianHallstatt (Fig. 5B and data S11).

The outgroup f3 analysis reveals that UkrIA_Chernyakhiv_3 has more affinity with Levantine/Anatolian early farmers on chrX compared to autosomes (fig. S6B and data S12 and S13), suggesting that its similarity to southern populations comes more from the female- rather than the male-mediated migration.

### East Asian as well as Eastern European genomes during the Middle Ages and early modern period

The mtDNA variation in individuals from medieval to early modern [Middle Ages and early modern period (MAEM); 900 to 1800 CE] Ukraine includes lineages that are frequent in Europe (mtDNA hgs U, H, J, and X2; Table 1 and data S1) as well as some that are more frequent in Asia (mtDNA hgs A, B, and C and also M65a and M7, that were not detected in the earlier periods; Table 1 and data S1). The chrY lineages include those that are common in modern Eastern Europe (R1a, R1b, N1a-Y10755, and G2a-Z6679), as well as lineages frequent in Siberia and East Asia (C2a-M504) (*65*) or the Near East (J1a-P58) (*66*) (Table 1 and data S1 and S2).

Two Cuman period individuals (900 to 1400 CE; UkrMA_Cuman), one from the Post-Cuman period (1300 to 1400 CE; UkrMA_Post-Cuman_Cuman?), and two Golden Horde–related nomads (1200 to 1400 CE; UkrMA_GoldenHorde_Nom) cluster with Western Steppe individuals on PCA but show more of the “East Asian” ancestry component using Admixture, when compared to preceding Scythians (Figs. 3D and 4 and figs. S1, S3, and S4). Another of the Cuman-associated individuals (991 to 1149 cal CE; UkrMA_Cuman_2) clusters with Central Asians, showing a larger eastern influence (Figs. 3D and 4 and figs. S1, S3, and S4). The White Croat Slav (1100 to 1300 CE; UkrMA_WhiteCroat_Slavs) and Golden Horde period individuals whose archaeological associations do not distinguish between Slavs and nomads (1200 to 1400 CE; UkrMA_GoldenHorde_Slav/Nom?) are similar to the first genetic subgroup of Chernyakhiv individuals and also to modern Ukrainians (Figs. 3D and 4 and figs. S1, S3, and S4). The Nogai-associated individuals of this study (1400 to 1500 CE) from Mamay-Gora in the Zaporizhzhia region can be divided into three genetic subgroups, the first of which (UkrMA_Nogai_1) is similar to the genetically more eastern Saltiv– and Cuman-associated individuals, while we infer an even larger eastern ancestry component in the remaining two subgroups (UkrMA_Nogai_2/3), the third clustering with Mongolians on PCA (Figs. 3D and 4 and figs. S1, S3, and S4). The two available Cossack Slav–associated individuals (1600 to 1800 CE; UkrEM_Cossack_Slavs) have the highest genetic affinity with the preceding Slav-related individuals as well as modern Ukrainians (Figs. 3D and 4 and figs. S1, S3, and S4).

The main group of Cuman individuals (UkrMA_Cuman) is cladal with the post-Cuman period individual (UkrMA_Post-Cuman_Cuman?) in an f4-based test but noncladal with Golden Horde nomads (UkrMA_GoldenHorde_Nom), the former having relatively higher genetic affinity with some Siberian and East Asian groups (data S5 and S6). In addition, the outlier Cuman individual (UkrMA_Cuman_2) is cladal with the preceding Central Asia–like Alans (UkrEMA_Saltiv_Alans_2) (data S5 and S6). White Croat Slavs (UkrMA_WhiteCroat_Slavs) are cladal with preceding, contemporary, and succeeding “Eastern/Central European” PCA cluster groups (UkrIA_Chernyakhiv_1, UkrMA_GoldenHorde_Slav/Nom?, and UkrEM_Cossack_Slavs) (data S5 and S6).

Using distal qpAdm modeling, “Western Steppe” PCA cluster groups (UkrMA_Cuman, UkrMA_Post-Cuman_Cuman?, and UkrMA_GoldenHorde_Nom) can be explained with around half of Ukraine_Yamnaya, some Ukraine_Trypillia, and some Mongolia_SlabGrave ancestry (35 to 62% + 18 to 39% + 14 to 28% overall) (Fig. 5A and data S8). Groups of the “Eastern/Central European” PCA cluster (UkrMA_GoldenHorde_Slav/Nom? and UkrEM_Cossack_Slavs) can be modeled as mostly Ukraine_Yamnaya, some Ukraine_Trypillia, and a small amount of Mongolia_SlabGrave ancestry (53 to 62% + 37 to 45% + 1 to 2% on average) (Fig. 5A and data S8). For the “Central Asia” cluster (UkrMA_Cuman_2 and UkrMA_Nogai_1), we infer some Ukraine_Yamnaya and Ukraine_Trypillia but around a half of Mongolia_SlabGrave ancestry (25 to 36% + 14 to 22% + 48 to 56% overall), while the East Asian–like Nogai subgroup (UkrMA_Nogai_3) can be modeled with a small amount of Ukraine_Trypillia, some Ukraine_Yamnaya, but mostly Mongolia_SlabGrave ancestry (6 ± 3% + 8 ± 4% + 85 ± 2%) (Fig. 5A and data S8).

Using proximal qpAdm modeling, we found that the outlier Cuman individual (UkrMA_Cuman_2) can be modeled as 100% UkrEMA_Saltiv_Alans_2, and the post-Cuman individual (UkrMA_Post-Cuman_Cuman?) can be modeled as 100% UkrMA_Cuman (Fig. 5B and data S11). Golden Horde nomads (UkrMA_GoldenHorde_Nom) can be modeled as 95 ± 4% UkrEIA_LateScythian_Cri_Nom and 5±4% Mongolia_SlabGrave ancestry, while the East Asian–like Nogai subgroup (UkrMA_Nogai_3) models as 43 ± 5% UkrEMA_Saltiv_Alans_2 and 57 ± 5% Mongolia_SlabGrave ancestry (Fig. 5B and data S11). Medieval groups of the “Eastern/Central European” PCA cluster (UkrMA_WhiteCroat_Slavs and UkrMA_GoldenHorde_Slav/Nom?) can be sourced from 100% UkrIA_Chernyakhiv_1 and the early modern group from the same cluster (UkrEM_Cossack_Slavs) in turn from 100% from UkrMA_WhiteCroat_Slavs (Fig. 5B and data S11).

### Heterogeneity in Ukraine and elsewhere in Western Eurasia

**Fig. 6.**
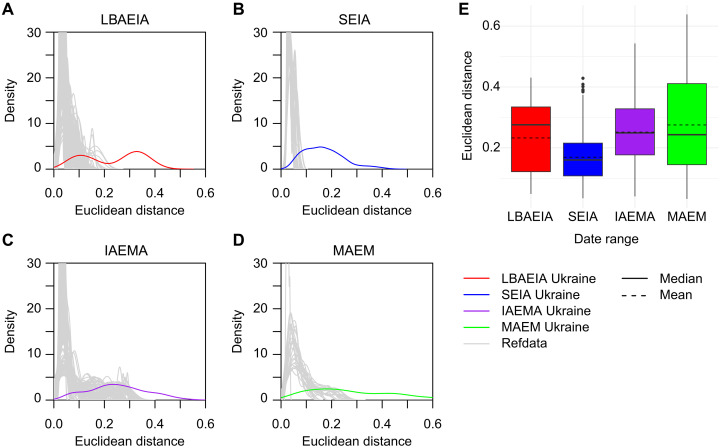
Investigating genetic heterogeneity in ancestry using PCA data. Kernel density estimations (KDE) of Euclidean distance sets for genomes of this study and corresponding comparative subsets (refdata), for (**A**) LBAEIA (up to 105 distances used for KDE, 100 comparative subsamples), (**B**) SEIA (up to 351 distances used for KDE, 63 comparative subsets), (**C**) IAEMA (up to 325 distances used for KDE, 100 comparative subsets), (**D**) Middle Ages and Early Modern period (MAEM, up to 171 distances used for KDE, 28 comparative subsets). A cutoff at density 30 is used on the *y* axis for better visibility of the Ukraine data. (**E**) Boxplot of Euclidean distances from the first 25 principal components of PCA analysis across date ranges (LBAEIA, *n* = 15; SEIA, *n* = 27; IAEMA, *n* = 26; MAEM, *n* = 19). Dashed lines indicate the mean.

As a means of estimating relative genetic heterogeneity, we analyzed multidimensional Euclidean distances between individuals from PCA (Fig. 6), grouped in the four date ranges discussed above: LBAEIA; SEIA; IAEMA; and MAEM (table S1). Considering the top principal components as continuous axes of covariation in ancestry, obtained through PCA following (*67*), we calculated Euclidean distances across the first 25 components to represent similarity or dissimilarity in genetic ancestry. This showed a general trend of high heterogeneity among the investigated individuals, with the exception of the SEIA date-group, for which it is somewhat decreased (Fig. 6E). To contextualize these PCA-based heterogeneity estimates, we generated a baseline for Western Eurasia over the same time periods. Up to 100 randomly geographically distributed subgroups of individuals (within the same geographic distance of one another as the Ukraine study individuals in each date range) were also analyzed in the same way. Plots of kernel density estimations for these show a shift toward higher Euclidean distances between the Ukraine genomes of this study when compared to the baseline in all date ranges. Notably, no matched random subsets from the Western Eurasia data returned higher average Euclidean distances than those obtained from the Ukraine genomes reported in this study (Fig. 6, A to D).

## DISCUSSION

The geographical location, landscape, and ecotypes of the Ukraine region have made it a place of intersection and interaction between eastern and western neighbors, which has left its mark on the genetic composition of local populations. The genetic profiles of Ukrainian Mesolithic and Neolithic HGs are intermediate between Eastern and Western European HGs (*6*, *36*, *40*). The following early farmers (associated with Trypillia and Globular Amphora Culture) are similar to those from the rest of Europe (*6*, *35*). Peoples of the Bronze Age Yamna culture from the east were not only assimilated in the North Pontic region but were also the source of demic flow further into Europe (*1*, *2*).

In the post-Yamna period, a clear genetic differentiation of the BA/EIA populations is observed. Eastern and steppe affiliated groups are genetically similar either to Yamna people (BA Zrubna and FBA Bilozerska individuals) or individuals from the eastern side of the Western steppe (FBA/EIA Cimmerians). The genomes of western forest-steppe individuals are more similar to those of Central/Eastern (FBA/EIA Vysotska and Lusatian individuals) and Southeastern (Thracian Hallstatt) Europeans. Notably, we detect a signal of sex-biased admixture evident in the Zrubna individuals. Since the Zrubna culture is a product of migration from the eastern Pontic Caspian steppe into Europe similarly to Corded Ware Culture (*8*, *16*), the scenario of the migrating people being mostly men who then admixed with local women in Europe, which has previously been suggested for Corded Ware Culture (*52*, *55*, *68*), is also plausible for Zrubna.

During the Scythian period, we infer a geographic structure that fits well with archaeology (*11*) and reflects their spread throughout the North Pontic steppe and forest-steppe. Most of the individuals associated with the Scythian culture from the western part of Ukraine (Illirian-Thracian basis) are genetically “local,” whereas genomes from the eastern part of Ukraine share more genetic ancestry with Western-Steppe and East Asian populations. Individuals from the eastern part of Ukraine, archaeologically associated with the “local elite,” have more Southeastern European influences (Near Eastern ancestry) in their genome (including Y hg E1b), when compared to “local agriculturalists.” Most of “nomad elite” individuals (Siversky Donets basin) have a genetic profile similar to that of people from the Caucasus. Patterns of genetic variation among social groups, as determined by the burial features and archaeological artifacts, appear more complex than those based on geography (Supplementary Text). Among the elite, there are both individuals with a local genetic profile and individuals with IA Western Steppe ancestry. There are intermediate profiles among local farmers and low-status nomads. Such patterns could be explained by the close incorporation of the Scythians into local society and vice versa, including the interweaving of elites as part of the population admixture process.

It is natural to expect a sex-biased admixture in the case of nomadic invasion, and our data indirectly support such assumptions: There are 8 males and 7 females among Scythian groups with a local archaeological background (right and left bank of Dnipro) but 11 males and only 3 females among groups with a nomad archaeological background (Siversky Donets, Northern Black Sea) (table S1). Scythian family burials in kurgans on both the right and the left banks of Dnipro and kinship groups for two generations established for eight samples indicate sedentary lifestyle, at least among a part of those with non-nomadic roots. The low mobility of Scythians in the North Pontic forest-steppe has previously been inferred using strontium (Sr) isotope analysis (*69*).

IA Chernyakhiv individuals from central and eastern forest-steppe of Ukraine form two genetic subgroups, one with a more “Northern/Central” and the other with a “Southern European” genetic profile, reflecting the polyethnicity of the Chernyakhiv group visible even within one location. The genetically more northern individuals may be potentially associated with the relocation of Goths into the North Pontic area. At the same time, the unusual Near Eastern maternal ancestry of the Chernyakhiv_3 individual from the Eastern Carpathians might help to explain previous archaeological observations. The Komariv-1 settlement, where this young woman was from, had the only known glass production point outside the Roman Empire (*70*, *71*); ancient glass production was concentrated primarily in the Middle East (*72*). The unusual Near Eastern ancestry might originate from eastern Mediterranean craft bearers living in the settlement.

The genetic composition of the EMA Alans and Bulgars examined in this study is similar to individuals from the Caucasus or Central Asia. The latter component indicates a constant influx of new migrants from Central Asia, which is consistent with previous archaeological claims (*73*). Our data suggest that the Central Asia–affiliated groups lived in the region permanently, consisting of both men and women whose genetic profiles, including autosomal and mitochondrial data, show no evidence of mixing with the local people. The high degree of genetic similarity among individuals thought to be Alans and Bulgars based on their burial ritual (catacombs versus pit burials, respectively) and other features is consistent with them coming from a single, genetically homogeneous population. It is unlikely that funeral rites provide a robust means of distinguishing Alans from Bulgars. This idea is consistent with other archaeological interpretations, such as not only catacombs but also pit burials of the Saltiv culture in the forest-steppe of Ukraine being associated with Alans, who came from the Caucasus (*74*).

The Cuman, Golden Horde, and Post-Cuman individuals of this study are genetically most similar to Western Steppe peoples. However, Nogai, the last nomadic group in the North Pontic, which is thought to have included remnants of Khazars, Pechenegs, Cumans, and Mongol peoples, has genetic profiles indicating high levels of East Asian ancestry. The Nogai nomadic migrants with East Asian ancestry, similarly to Alans described above, include both males and females without genomic signals of admixture with autochthonous people. The genetic composition of contemporary medieval Slavs is similar to succeeding Ukrainian Cossacks and modern Ukrainians. Moreover, such genetic profiles are traceable among some individuals from previous periods since LBA and, apparently, are of local Eastern European ancestry.

DNA analyses of samples taken from archaeological sites representing different regions of Ukraine in the chronological interval from around 9000 BCE to 1800 CE show that the ancient population had a diverse range of ancestries as a result of frequent movements, assimilation, and contacts. From the Mesolithic until the time of Vysotska and Bilozerska cultures at the end of the Bronze Age, broad-scale ancestry proportions are similar to contemporary populations in the rest of Europe—first HGs, then early farmers, and lastly a mixture between early farmers and Steppe pastoralists. Starting from the Cimmerian time (EIA) until the Middle Ages, the appearance of eastern nomads in the Pontic region became a regular occurrence. Their genetic composition varied from Yamna-like superimposed on the locals, as with Scythians and Cumans, to high degrees of East Asian ancestry and minimal local admixture, as with Alans-Bulgars and Nogai. During that time, nomadic populations were recorded in the steppe zone, whereas individuals from the rest of the Ukrainian region had mostly European ancestry, associated with local predecessors, as well as Thracians, Greeks, Goths, etc.

The palimpsest of migration and population mixing in the Ukraine region will have contributed to the high genetic heterogeneity in geographically, culturally, and socially homogeneous groups, with different genetic profiles present at the same site, at the same time and among individuals with the same archaeological association. It is important to note that this study has a particular focus on historically attested migrating groups rather than local populations, and sampling is geographically skewed mostly toward the eastern part of Ukraine and temporally toward the Iron Age and the medieval period. Nevertheless, the broad-scale local genetic profile, which is similar to modern Ukrainians, persists in the region through time also within this sample set. This ancestry composition can be traced back to the Zrubna individuals at least and is seen among Vysotska and Lusatian individuals, Scythians from the west and contemporary agriculturalists from the east, among the Chernyakhiv population, as well as medieval and early modern Slavs. Despite clear signatures of high migration activity, including from East Asia, as well as extensive admixture, we infer a major autochthonous component to Ukrainian ancestry, at least since the Bronze Age.

## MATERIALS AND METHODS

### Experimental design

The teeth used for DNA extraction were obtained with relevant institutional permissions from various collections in Ukraine: Museum of Archaeology of V. N. Karazin Kharkiv National University, Zaporizhzhia National University, I. Krypyakevich Institute of Ukrainian Studies of the National Academy of Sciences of Ukraine, Institute of Archaeology of National Academy of Sciences of Ukraine, Odesa Archaeological Museum, M. F. Sumtsov Kharkiv Historical Museum, Laboratory of Archeology and Ethnology of V. O. Sukhomlynskyi Mykolaiv National University, Mykolaiv Regional Museum of Local History, Center for the preservation and preservation of monuments of archaeology of the Poltava region for the sake of Poltava, Archaeological Research Laboratory of National Pedagogical Dragomanov University, and Lviv Historical Museum.

DNA was extracted from 129 tooth or bone samples from ancient individuals from the territory of present-day Ukraine—2 from the Neolithic (UkrN; 7000 to 6000 BCE), 14 from the Bronze Age to the beginning of the Iron Age (UkrBA and UkrFBA/EIA; 3000 to 700 BCE), 7 from the beginning of the Early Iron Age (UkrEIA; 900 to 700 BCE), 46 from the Scythian period of the Early Iron Age (UkrEIA; 700 to 300 BCE), 8 from the end of the Early Iron Age (UkrEIA; 400 to 1 BCE), 16 from the later Iron Age (UkrIA; 1 to 400 CE), 12 from the Early Middle Ages (UkrEMA; 800 to 900 CE), and 24 from the Middle Ages to the early modern period (UkrMA and UkrEM; 900 to 1800 CE) (Fig. 2 and data S1). More detailed information about the archaeological periods and the specific sites and burials of this study is given in Supplementary Text.

Sampling and DNA extraction were performed in dedicated aDNA laboratories of the Institute of Genomics, University of Tartu. Extract purification and library preparation were performed in dedicated aDNA laboratories of the Francis Crick Institute. DNA sequencing was performed at the Advanced Sequencing Facility, the Francis Crick Institute. The main steps of the laboratory work are detailed below. For six samples (UKR008, UKR030, UKR053, UKR070, UKR119, and UKR147), sampling, DNA extraction, extract purification, and library preparation were performed in dedicated aDNA laboratories of the Department of Archaeology and Anthropology, University of Cambridge as described in (*7*), and sequencing was performed at the Cambridge Biochemistry DNA Sequencing Facility and the Institute of Genomics Core Facility, University of Tartu.

### Laboratory methods

#### DNA extraction

For 109 teeth, the apical tooth roots were cut off using a drill with a cutting wheel attached and used for extraction since root cementum has been shown to contain more endogenous DNA than crown dentine (*75*). The root pieces were used whole to avoid heat damage during powdering with a drill and to reduce the risk of cross-contamination between samples. Contaminants were removed from the surface of tooth roots by soaking in 6% bleach for 5 min, then rinsing three times with Milli-Q water (Millipore), and lastly soaking in 70% ethanol for 2 min, shaking the tubes during each round to dislodge particles. Then, the samples were left to dry under an ultraviolet light for 2 hours. Next, the samples were weighed, [20 * sample mass (mg)] μl of EDTA and [sample mass (mg)/2] μl of proteinase K were added, and the samples were left to digest for 72 hours on a rotating mixer at 20°C to compensate for the smaller surface area of the whole root compared to powder.

For 14 bone fragments, the surface of the bone fragment was cleaned using a drill with a ball-end drill bit attached, and powder from the inner part of the cortical bone was collected for extraction. Next, the samples were weighed, [20 * sample mass (mg)] μl or 1 ml (if >50 mg of powder) of EDTA were added, and the samples were left to digest for 24 hours on a rotating mixer at 20°C.

The lysate was concentrated to 250 μl [Vivaspin Turbo 15, 30,000 molecular weight cut-off (MWCO) polyethersulfone (PES), Sartorius], and 140 μl of concentrated lysate was transferred to a clean tube, frozen, and shipped to the Francis Crick Institute on dry ice. An undigested material and the leftover lysate were stored for a second DNA extraction if need be. At the Francis Crick Institute, the concentrated lysate was transferred into FluidX tubes where it underwent automated purification on an Agilent Bravo Workstation (*76*).

#### Library preparation

Single-stranded DNA libraries were prepared from the extracts using an automated system (*77*). No treatment to remove uracils was used. The libraries were then double-indexed (*78*).

#### DNA sequencing

DNA was first sequenced using the Illumina HiSeq 4000 platform, and around 2.5 million sequencing reads were generated per sample. On the basis of these data, 88 of 123 samples were chosen to be included in further analyses. For most of the additional sequencing, fragments smaller than 35 bp were removed from the library (*77*). For this, 100 ng of the initial library was biotinylated, and the nonbiotinylated strand was isolated using streptavidin beads to obtain a single-stranded library. The samples were pooled and loaded onto a denaturing polyacrylamide gel along with 30-, 35-, and 150-bp insert markers, and fragments with the desired length were excised and eluted from the gel after incubating overnight. Around 15.5 billion sequencing reads were generated using the Illumina NovaSeq platform with the 100-bp paired-end method. The six samples processed in Cambridge were sequenced using the Illumina NextSeq 500 platform with the 75-bp single-end method, and four were chosen to be included in further analyses.

### Statistical analysis

#### Mapping and quality filtering

Sequencing data were processed using the nf-core/eager v2 pipeline (*79*). First, adapters were removed, paired-end reads were merged, and bases with a quality below 20 were trimmed using AdapterRemoval v2 (*80*) with options –trimns –trimqualities –collapse –minadapteroverlap 1 and –preserve5p. Next, reads with a minimum length of 35 bp were mapped to the hs37d5 human reference genome using Burrows-Wheeler Aligner (BWA-0.7.17 aln) (*81*) and parameters -l 16500 -n 0.01 -o 2 -t 1 (*78*, *82*). Then, the mapped BAM files were deduplicated using Dedup (*83*). Last, data from different sequencing runs were merged, duplicates were removed with Picard 2.20.3 (https://broadinstitute.github.io/picard/), and reads with a mapping quality under 10 were filtered out with SAMtools 1.9 (*84*).

The average endogenous DNA content (proportion of reads mapping to the human genome) for the 129 samples is 35% (data S1). The endogenous DNA content is extremely variable as is common in aDNA studies, ranging from under 0.01% to almost 95% (data S1). The average endogenous content for the 92 samples chosen for further analyses is 49%, varying between 1% and almost 95% (data S1).

#### aDNA authentication

aDNA damage in the form of C to T substitutions in the 5′ ends of sequences due to cytosine deamination was estimated using DamageProfiler (*85*) from the nf-core/eager v2 pipeline (*79*). mtDNA contamination estimation was performed using schmutzi (*86*).

For the male individuals, contamination was also estimated on the basis of chrX using the two contamination estimation methods first described by Rasmussen *et al.* (*87*) and incorporated in the Analysis of Next Generation Sequencing Data (ANGSD) software (*88*) in the script contamination.R.

The samples show 23% C=>T substitutions at the 5′ ends on average, ranging from 0 to 61% in all samples and from 8 to 50% in the samples chosen for further analyses (data S1). The mtDNA contamination point estimate for the samples chosen for further analyses ranges from 1 to 8% with an average of 2% (data S1). The average of the two chrX contamination methods of male individuals chosen for further analyses with average chrX coverage >0.1× is 2% on average, ranging between 0 and 3% for most individuals (data S1). For two individuals, the chrX contamination estimate is >10% (data S1), but given that the mtDNA contamination estimate is low, average read length is similar to other samples and the C=>T substitution rate is high for both individuals, it was decided to include them in analyses, keeping the potential contamination in mind.

#### Kinship analysis

A total of 4,375,438 biallelic single-nucleotide variant sites, with a minor allele frequency (MAF) of >0.1 in a set of more than 2000 high coverage genomes of Estonian Genome Center (*89*), were identified and called with ANGSD (*88*) command --doHaploCall from the BAM files of all Iron Age samples (*n* = 67), including 13 from the same groups published in (*7*), and all medieval (and early modern) samples (*n* = 28) that were chosen for further analyses. Furthermore, the process was repeated for all 39 Scythian period samples and for smaller groups of samples with specific archaeological associations—7 Thracian Hallstatt, 10 right bank of Dnipro Schytian, 10 left bank of Dnipro Scythian, 15 Siversky Donets basin Scythian, 4 Northern Black Sea region Scythian, 14 Chernyakhiv, 9 Saltiv, and 7 Nogai. The Neolithic and Bronze Age samples were too few per group and spread over a too long time period to be included in kinship analyses. The ANGSD output files were converted to .tped format as an input for the analyses with READ script to infer pairs with up to second degree relatedness (*57*). The same groups and sites were used to infer pairs with up to third degree relatedness with KIN (*58*), which confirmed the relative pairs identified with READ and specified the second degree related pairs between parent-child and sibling relationships.

The results based on smaller groups and wider time periods are consistent with each other. Next to first and second degree relative pairs, two identical pairs of samples are revealed (fig. S5). The first pair of identical samples—UKR035 and UKR038 (fig. S5A)—was thought to come from two skeletons in the same burial so we deduce that they most likely come from the same individual, and the data were merged (UKR035AB) with samtools 1.9 (*84*) option merge. The second pair—UKR089 and UKR091 (fig. S5B)—come from the same site but from two different kurgans excavated on different years so we consider that these could be identical twins. Hence, data from 91 newly sequenced individuals were used in further analyses. For group-based analyses, only the highest coverage individual of each group of relatives was used.

#### Calculating general statistics and determining genetic sex

Samtools 1.9 (*84*) option stats was used to determine the number of final reads, average read length, average coverage, etc. for the 91 genomes. Genetic sex was calculated using the script sexing.py from (*90*), estimating the fraction of reads mapping to chrY out of all reads mapping to either X or chrY.

The average coverage of the whole genome for the 91 individuals is between 0.019× and 1.95× (data S1). Of these, 13 genomes have an average coverage of less than 0.1×, 9 genomes >0.1×, 34 genomes >0.3×, 27 genomes >0.5×, and 8 genomes >1× (data S1). Genetic sexing reveals that the study involves 44 females and 47 males (Table 1 and data S1).

#### Determining mtDNA hgs

The program bcftools (*91*) was used to produce Variant Call Format (VCF) files for mitochondrial positions—genotype likelihoods were calculated using the option mpileup, and genotype calls were made using the option call. mtDNA hgs were determined by submitting the mtDNA VCF files to HaploGrep2 (*92*, *93*). hgs were successfully determined for all 91 individuals (Table 1 and data S1).

#### chrY variant calling and haplogroup determination

In total, 273,059 haplogroup informative chrY variants from regions that uniquely map to chrY (*94*–*98*) were called as haploid from the BAM files of the samples using the --doHaploCall function in ANGSD (*88*). Derived and ancestral allele and haplogroup annotations for each of the called variants were added using BEDTools 2.29.2 (*99*) intersect option. Haplogroup assignments of each individual sample were made manually by determining the haplogroup with the highest proportion of informative positions called in the derived state in the given sample. chrY haplogrouping was blindly performed on all samples regardless of their sex assignment.

No female samples have reads on the chrY consistent with a haplogroup, indicating that levels of male contamination are negligible. hgs for 45 (with a coverage of >0.01×) of the 47 males were successfully determined (Table 1 and data S1 and S2).

#### Genome-wide variant calling

Genome-wide variants were called with the ANGSD software (*88*) command --doHaploCall, sampling a random base for the positions that are present in the AADR (*42*) version 54.1.

#### Preparing the datasets for autosomal analyses

Individuals from the Human Origins (HO) array dataset from AADR (*42*) version 54.1 were used as the modern DNA background. Individuals from the 1240K dataset from AADR (*42*) version 54.1 were used as the aDNA background.

The data of the comparison datasets and of the individuals of this study were converted to BED format using PLINK 1.90 (https://cog-genomics.org/plink/) (*100*), and the datasets were merged. Two datasets were prepared for analyses: one with HO and 1240K individuals and the individuals of this study, where 577,902 autosomal SNPs of the HO dataset were kept; the other with 1240K individuals and the individuals of this study, where 1,127,956 autosomal and 45,218 chrX SNPs of the 1240K dataset were kept. Individuals with <10,000 SNPs overlapping with the HO autosomal dataset were removed from further autosomal analyses.

#### Principal components analysis

To prepare for PCA, two reduced comparison sample sets were assembled, both including 3298 ancient individuals from 400 populations, while one composed of 813 modern individuals from 54 populations of Europe, Caucasus, and Near East (West Eurasia) and the other included in addition 1230 modern individuals from 86 populations (Eurasia) (data S3 and S4). Only SNPs with a MAF of >0.05 in the HO dataset were used (*n* = 409,113). The data were converted to EIGENSTRAT format using the program convertf from the EIGENSOFT 8.0.0 package (*67*). PCA was performed with the program smartpca from the same package, projecting ancient individuals onto the components constructed based on the modern genotypes using the option lsqproject.

#### Admixture analysis

For Admixture analysis (*101*), the same ancient sample set was used as for PCA, and the modern sample set included 1861 individuals from 144 populations from all over the world (data S3 and S4). Only SNPs with MAF of >0.05 in the HO dataset were used, and the HO dataset of modern individuals was further pruned to decrease linkage disequilibrium using the option indep-pairwise with parameters 1000 250 0.4 in PLINK 1.90 (https://cog-genomics.org/plink/) (*100*). This resulted in a set of 161,774 SNPs. Admixture was run using ADMIXTURE 1.3 (*101*) on the modern dataset using *K* = 3 to *K* = 14 in 100 replicates. This enabled us to assess convergence of the different models. *K* = 10 was the model with the largest number of inferred genetic clusters for which >10% of the runs that reached the highest log likelihood values yielded very similar results. This was used as a proxy to assume that the global likelihood maximum for this particular model was reached. Then, the inferred genetic cluster proportions and allele frequencies of the best run at *K* = 10 were used to run Admixture using ADMIXTURE 1.3 (*101*) with the P option to project the aDNA individuals. The same projecting approach was taken for all models (*K* = 3 to *K* = 14). We present all ancient individuals on fig. S4 (A and D) but only population averages on Fig. 4.

Admixture was also run using only ancient individuals and without projection. For this, the same ancient sample set was used but with the SNPs of the 1240K dataset. The dataset was pruned to decrease linkage disequilibrium using the option indep-pairwise with parameters 50 10 0.1, resulting in a set of 366,718 SNPs. Samples with more than 90% of missing data were removed with the mind option in PLINK 1.90 (https://cog-genomics.org/plink/) (*100*). Next, the comparative dataset was revised to include a maximum of five individuals per group, resulting in 1436 individuals to be used in the analysis, including 79 individuals of this study. Admixture was run using ADMIXTURE 1.3 (*101*) using *K* = 2 to *K* = 6 in 100 replicates. This enabled us to assess convergence of the different models. *K* = 4 was the model with the largest number of inferred genetic clusters for which >10% of the runs that reached the highest log likelihood values yielded very similar results. The inferred genetic cluster proportions and allele frequencies were also used to run Admixture using ADMIXTURE 1.3 (*101*) with the P option to project all aDNA individuals for comparison. We present all ancient individuals on fig. S4 (B, C, E, and F) but only population averages on fig. S3—based on nonprojected results for groups that could be included in that analysis, based on projected results for other groups.

#### f4 statistics

For calculating f4 statistics, the SNPs of the 1240K dataset were used with Mbuti as outgroup and the same ancient sample set as for previous analyses but only including comparative groups with at least three individuals, resulting in 3138 individuals from 314 populations (data S3 and S4). The data were converted to EIGENSTRAT format using the program convertf from the EIGENSOFT 8.0.0 package (*67*). f4 statistics of the form f4 (Mbuti, other ancient; Ukraine ancient, Ukraine/other relevant ancient) were calculated using the ADMIXTOOLS 2 (*102*, *103*) package program qpDstat.

#### Outgroup f3 statistics

Autosomal outgroup f3 statistics were calculated on the same dataset as f4 statistics (data S3 and S4). To allow for chrX versus autosome comparison for ancient populations, outgroup f3 statistics were run both using 1,127,956 autosomal SNPs and also 45,218 chrX positions available in the 1240K dataset. Since all children inherit half of their autosomal material from their father but only female children inherit their chrX from their father, then in this comparison, chrX data give more information about the female ancestors of a population, and autosomal data give more information about the male ancestors of a population. Outgroup f3 statistics of the form f3(Mbuti; Ukraine ancient, other ancient) were computed using the ADMIXTOOLS 2 (*102*, *103*) package program qp3Pop. Results for combinations including UkrN_?, UkrFBA/EIA_Vysotska_Early, UkrEIA_Scythian_SivDon_NomEl_2, SaltovoMayaki, and China_Wuzhuangguoliang_LN were not included in chrX versus autosome comparisons since <1000 SNPs were used in many of these combinations based on chrX data.

#### qpAdm

The ADMIXTOOLS 2 (*102*, *103*) package program qpAdm was used to estimate which populations and in which proportions are suitable proxies of admixture to form the groups of this study. The autosomal positions of the 1240K dataset were used. Only samples with more than 100,000 SNPs were used in the analyses. Mota, Ust-Ishim, Kostenki14, GoyetQ116, Vestonice16, MA1, AfontovaGora3, ElMiron, Villabruna, Afanasievo, Anatolia_N, and Mongolia_North_N were used as “right” populations. For distal models, Yamnaya_Samara, Yamnaya_Kalmykia, or Ukraine_Yamnaya was used as the “left” population representing Steppe ancestry; LBK_EN, Central_MN, Poland_GlobularAmphora, Ukraine_GlobularAmphora, or Ukraine_Trypillia was used as the left population representing early farmer ancestry; and Mongolia_LBA_Khovsgol_6, Mongolia_LBA_CenterWest_4, or Mongolia_EIA_SlabGrave_1 was used as the left population representing East Asian ancestry. For proximal models, groups of this study from earlier time periods were used as the left populations representing local ancestry, and Mongolia_EIA_SlabGrave_1 was used as the left population representing East Asian ancestry.

#### Comparing genetic heterogeneity

Analysis of genetic heterogeneity made use of eigenvectors obtained from the preceding PCA analysis to compare Euclidean distances between genomes in multidimensional principal components space. Up to 100 principal components were generated using the smartpca method (*67*) from the EIGENSOFT package (detailed above). Ukraine genomes generated in this study were subset into the following date ranges based on the midpoints of the 95% calibrated date estimates or archaeological date range estimates: LBAEIA (3000 to 700 BCE, *n* = 15); SEIA (699 to 300 BCE, *n* = 27); IAEMA (299 BCE to 900 CE, *n* = 26); and MAEM (901 to 1800 CE, *n* = 19). One Neolithic (UKR008), two Scythian-associated (UKR095 and UKR096), and one potentially Greek-associated genome (UKR153) were excluded from the analysis due to falling outside of the specified date range. To contextualize these PCA-based heterogeneity estimates, comparative datasets were generated from 3298 previously published ancient genomes from Western Eurasia. We subset these genomes into the same four date ranges as for the Ukraine data. From the geographic coordinates of the individuals of this study, the radius of the minimal bounding circle was obtained for each date range, using the haversine formula implemented in R to calculate great-circle distances. From each of the comparative data date ranges described above, sets of subsamples were generated by randomly selecting up to 100 individuals and then, for each of these individuals, retaining all other reference genomes within the same radius distance as the corresponding Ukraine data. This resulted in up to 100 randomly positioned subsets of equivalently proximate individuals, setting a minimum of 10 individuals per subset. As this was limited by the number of unique individuals with >10 others within the radius distance, the resulting number of subsets was in some cases <100 (LBAEIA, *n* = 100; SEIA, *n* = 63; IAEMA, *n* = 100; MAEM, *n* = 28). For each date range, pairwise Euclidean distances in principal component space were then determined between the genomes of this study and also between the genomes in each comparative subset using the first 25 principal components, scaling each eigenvector by its eigenvalue. Boxplots and kernel density estimates were then generated in R to visually compare the resulting sets of distances. All these methods are documented in a reproducible Rscript (see Data and materials availability).

## Supplementary Materials

### The PDF file includes:

Supplementary Text Figs. S1 to S7 Legends for data S1 to S14 References
