# Supplementary material for: North Pontic crossroads: Mobility in Ukraine from the Bronze Age to the early modern period
Source: Sci Adv. 2025 Jan 8;11(2):eadr0695. doi: 10.1126/sciadv.adr0695 (PMC11708899; doi:10.1126/sciadv.adr0695)
Supplement: Supplementary file 1 — Supplementary Text Figs. S1 to S7 Legends for data S1 to S14 References [file sciadv.adr0695_sm.pdf]

Science Advances
AAAS

Supplementary Materials for

North Pontic crossroads: Mobility in Ukraine from the Bronze Age to the early modern period

Lehti Saag *et al.*

Corresponding author: Lehti Saag, lehti.saag@ut.ee; Pontus Skoglund, pontus.skoglund@crick.ac.uk; Mark G. Thomas, m.thomas@ucl.ac.uk

Sci. Adv. **11**, eadr0695 (2025)
DOI: 10.1126/sciadv.adr0695

The PDF file includes:

Supplementary Text
Figs. S1 to S7
Legends for data S1 to S14
References

Other Supplementary Material for this manuscript includes the following:

Data S1 to S14

Supplementary Text

<u>Archaeological and anthropological background</u>

NEOLITHIC

The Neolithization of the territory of present-day Ukraine occurred during 7,000–5,000 BCE and spread from the Balkans through the Danube area via several migration waves, involving tribes of the Linear Pottery, Cucuteni-Trypillia and some other European cultures. Neolithic archaeological cultures found on the territory of Ukraine are Bug-Dniester culture, Dnipro-Donets culture, Azov-Dnipro culture, Pit-Comb Ware culture, etc. (*106*)

<u>Azov-Dnipro (Mariupol) culture</u> (Азово-Дніпровська культура)

The landmarks of the Sur and Azov-Dnipro cultures are known from the Lower Dnipro region. The Azov-Dnipro culture landmarks are dated from the 6th to the beginning of the 5th millennium BCE. They are represented by both settlements (Semenivka, Stone Grave) and burial grounds (Lysa-Gora, Mamay-Gora, etc.). (*107*)

Mamay-Gora (Mamai-Hora) *(Мамай-Гора)*

(Archaeology, text – G. Toshev, S. Andrukh)

The Mamay-Gora hill is a multi-layered archaeological site located near the village Velyka Znamianka in the Vasilyevsky district (former Kamensko-Dneprovsky) of the Zaporizhzhia region, on the high left bank of the Kakhovka reservoir. This elevated location was used by steppe tribes as a burial place from the Neolithic to the 15th c. CE. Now the burial ground occupies approximately 30 hectares. Its core is formed by 5 kurgans (three elongated and two round), stretching along the west-east line. (*108–112*)

Since 1988, Mamay-Gora has been studied by archaeological expeditions of the Zaporizhzhia National University. During more than 34 years over 900 burial complexes (kurgans and ground burials) have been found there. The oldest are two Neolithic burial grounds, followed by the complexes of the Eneolithic, Bronze Age, and Scythian time (n=398). Later, the Sarmatians (n=2), the tribes of the post Golden Horde time (Cumans (n=60), Nogai (n=200)) were buried there. Currently the excavations are interrupted because of the Russian aggression in Ukraine.

The Neolithic burial ground under research is a family burial belonging to the first period of the Azov-Dnipro culture. It comprises 26 graves forming 2 rows stretched perpendicular to the Dnipro riverbed. Burials are in individual pits at different distances from each other. The skeletal remains lay stretched out on backs, with heads facing south or west. In most of the burials, artefacts were found: pendants made of red deer's teeth, necklaces made of shells or stones, flint plates. In 13 burials, ochre was found on the bones, on the bottom of the pit or in the filling. Among the buried individuals there were men (n=6), women (n=4), unidentified adults (n=9) and children (n=7). (*113*)

Location: 47.432845 N, 34.27259 E. Mamay-Gora tract, Velyka Znamianka village, Vasylivsky District, Zaporizhzhia Region.

Excavations: 1999.

Excavation authors: Svitlana I. Andrukh, Gennadi M. Toshev*,* Zaporizhzhia National University, Zaporizhzhia, Ukraine.

Storage of anthropological materials: Zaporizhzhia National University, Zaporizhzhia, Ukraine.

Description of samples:

One sample was taken for aDNA analysis.

UKR008. *Mamay-Gora, Neolithic burial 26.* Excavated in 1999. Human remains were found at a depth of 1.17 m. The contours of the pit were not traced. The individual was stretched out on the back with hands along the body. The head was oriented to the southeast/east. Chronology according to archaeology: 7,000–6,000 BCE. (*111*)

BRONZE AGE

Zrubna (Srubnaya)/Timber-Grave cultural and historical community (Зрубна культура)

(Archaeology - V. Mikheev; text – I. Shramko, S. Zadnikov)

The Zrubna cultural and historical community was formed in the Late Bronze Age (1,800–1,100 BCE) and spread in the steppe and forest-steppe zones of Eastern Europe from the Dnipro river to the Ural mountains. The main variations are Berezhniv-Mayivska and Pokrovska Zrubna cultures. The Pokrovska Zrubna culture (1,800–1,400 BCE) was widespread in the steppe and forest-steppe zones from Siversky Donets river to the Volga river. Separate sights are presented in the Urals. The Berezhniv-Mayivska Zrubna culture (1,700–1,100 BCE) was widespread in the steppe and forest-steppe area from Ingulets river to the Volga river. The main types of archaeological sites are settlements, kurgans and ground burials. The economy was based on cattle breeding and agriculture. Bronze casting production and bone processing were also developed.

Sukha Gomilsha *(Суха Гомільша)*

The kurgan is located near the centre of the northern part of the Sukha Gomilsha hillfort belonging to the Saltiv culture (8th to 10th c. CE). It stands out in the area like a small hill of 0.2–0.4 m in height and 21–22 m in diameter.

Location: 49.547122 N, 36.362542 E. Sukha Gomilsha village, Slobozhanska territorial community, Chuhuiv district, Kharkiv region.

Excavations: 1982.

Excavation authors: Volodymyr K. Mikheev, V. N. Karazin Kharkiv National University, Kharkiv, Ukraine.

Storage of anthropological materials: Collection of the Museum of the Archaeology, V. N. Karazin Kharkiv National University, Kharkiv, Ukraine.

Description of samples:

One sample was taken for aDNA analysis.

UKR055. *Sukha Gomilsha, burial 3.* Excavated in 1982. Inlet burial in kurgan. A human skeleton of poor preservation was found at a depth of 1.0 m from the modern surface level. The skeleton lay on the back, arms stretched along the body. The head was oriented to the west. There was a

vessel near the head. The burial belonged to the Late Bronze Age and dated to 1,300–800 BCE according to archaeology. (*114*, *115*) Chronology according to ^{14}C dating: 1873–1566 cal BCE (3404±35 BP).

FINAL BRONZE AGE

Bilozerska culture *(Білозерська культура)*

(Archaeology, text – K. Gorbenko)

The Bilozerska culture is an archaeological culture of the Final Bronze Age (1,200–900 BCE). It was defined as an archaeological culture by V.V. Otroshchenko, I.T. Chernyakov and V.P. Vanchugov in the 1980s. The name comes from the settlement on the shore of the Bilozersky estuary (now the Kakhovka Reservoir) in the city of Kamianka-Dniprovsk. The culture is widespread in the steppe zone of Ukraine and Moldova. A few sites were found on the Lower Don, on the territory of the Kuban and in Crimea.

The sites of the Bilozerska culture are represented by settlements alongside rivers and estuaries (Tudorovka, Voronivka, Zmiivka), kurgans (Shyroka Mohyla, Stepovy, Kochkovate) and ground burials (Brylivka, Shiroke, Budurzhel), complexes of foundry molds (Zavadivka, Novooleksandrivka), foundry workshops (Kardashinka), treasures of metal objects (Novogrigoryivka) and others. Presumably, the carriers of the culture tried to control the supply routes for raw materials, prestige products, and possibly grain.

In the Prut-Dniester interfluve, the Bilozerska culture has three periods (S. Agulnikov): early (end of the 13th to first half of the 12th c. BCE); middle (end of the 12th to first half of the 11th c. BCE); late (second half of the 11th to first half of the 10th c. BCE). Represented mainly by settlements, kurgans, ground burials, hillfort (Dyky Sad).

Approximately 165 settlements of Bilozerska culture are known. The maximum area of these reached 3.0–4.5 hectares. The layout of the settlements was street-type. The size of houses ranged from 15 to 90 m^2.

Burials are connected with settlements and are best studied in the places where the settlements are clustered. So far, more than 800 burials have been identified. The population of the Bilozerska culture used a burial tradition that was different from the prevailing one in the steppe. Old graves were almost not used for burial needs. People built new graves or used ground cemeteries (kurgan, ground grave).

The population was heterogeneous. Anthropologists note the similarity of Bilozerska people with narrow-faced skulls from ground cemeteries belonging to the Noua culture. A common anthropological dynamic is traced for Bilozerska culture and Ukrainian Zrubna culture. In general, the local anthropological basis of Bilozerska culture seems clear, although external influences took place all over the Bilozerska culture world. (*116–123*)

Dykyi Sad fortified settlement *(Городище Дикий сад)*

Dykyi Sad (Wild Garden) fortified settlement is located on a plateau at the confluence of the Southern Bug and Ingul rivers, in the historical centre of the modern city of Mykolaiv. It was built in the form of an oval elongated along the SE-NW axis. The total area of the preserved territory reaches more than 5 hectares. The settlement belongs to the Bilozerska archaeological culture

(Bilozerska-Tudorovska community) of the Final Bronze Age (13/12–12/11 c. BCE). According to ^{14}C dating, the time ranges within 1,186–925 BCE.

Residential, economic, defence, ritual and cult objects have been found on the territory of the settlement. It can be argued that during the heyday of the settlement, a clear system of uniform planning and construction was formed within the territory: a "citadel" surrounded by a moat, a "suburb" in the hemisphere of the outer moat, a "market". The plan of the settlement corresponds to the classic concept of "urbs" ("city").

In total, 53 archaeological sites in an area of more than 8,000 m^2 have been investigated. Among them are 41 premises with yards, 3 utility pits outside the "suburb", a moat around the "citadel" (with buried skulls), a moat around the entire settlement, remains of defensive structures along the moat of the citadel, ritual and cult ramp, the central site of the "citadel", the economic and ritual site opposite the moat of the "citadel", the central square of the remote "suburb", 21 pits for economic and ritual purposes, a pit behind the outer moat. The pits were located on a flat area between the houses, forming a kind of central economic area of the "suburb". Remains of ceramic dishes, burials of human skulls, animal and fish bones, remains of charred grains of common millet, barley, wheat, cultivated grapes and charcoal were found in the pits. (*120*, *121*, *124*, *125*)

Location: 46.980272N, 31.983500E. Mykolaiv city (Naberezhna str.), Mykolaiv region.

Excavations: 2004, 2007.

Excavation authors: Kyrylo V. Gorbenko, Petro Mohyla Black Sea National University, Mykolaiv, Ukraine.

Storage of anthropological materials: Mykolaiv Regional Museum of Local History, Mykolaiv, Ukraine.

Description of samples:

Two samples were taken for aDNA analysis.

UKR149. *Dykyi Sad, the moat of the hillfort "citadel". Excavation 13, lower layer.* The arc-shaped moat encloses the "citadel" of the settlement, stretching along the southeast-northwest axis. The length of the studied part is 130 m (the total length is approximately 140–150 m), width 5.0 m, depth 3.0 m. In the southern part of the moat, the stone foundation of a bridge (large limestone slabs) is located. The foundation is rectangular in plan, stretched across the moat along the north-south line with a slight deviation to the northeast. The dimensions are 3.64x2.0 m, and the width of the central part is 1.4 m due to the shifting of the stones. At the distance of 5 m to the west of the foundation, in the middle of the stone lining and among ceramic fragments, a human (female) skull was found. Chronology according to archaeology: 13–12 c. BCE.

UKR150. *Dykyi Sad, the central site of the distant "suburb" of the settlement. Pit № 8.* The ritual pit had a rounded shape with an extension to the lower part. The upper and lower diameters were 0.5 m and 1.05 m, depth was 0.65 m. The pit was filled with grey humus loam (burnt soil), small and medium-sized rubble. At a depth of 0.25 m there was a stone backfilling (143 stones). Pot remains were found in the eastern part of the pit at a depth of 0.5 m. In the southern part at a depth of 0.45 m there was a human skull without a lower jaw, facing west. The skull was covered with small stones. The bottom of the pit was flat and clayey. Chronology according to archaeology: 12 c. BCE.

FINAL BRONZE AGE / EARLY IRON AGE

Vysotska culture *(Висоцька культура)*

(Archaeology, text – M. Bandrivskyi)

The Vysotska culture was one of the bright archaeological cultures of the end of the Bronze Age and the beginning of the Iron Age in Central-Eastern Europe. It occupied a small area in the west of Ukraine: from the upper course of the Zbruch river in the east to the upper course of the Western Bug in the west, and from the Volyn upland in the north to the middle course of the Seret and Strypa rivers in the south. Graveyards and individual burials, including kurgans, are the source of knowledge about this culture since settlements are almost unknown today.

The culture's chronology includes three periods: early – from around 1250–1150 BCE (Bronze Age to Hallstatt period A1 (HaA1)); middle – 1150–950/920 BCE (HaA2–HaB1); late – 950/920–around 725 BCE (HaB3–HaC1).

As a rule, the burials were oriented to the south, graves were placed in rows, grave pits were almost completely absent (the remains were buried in the lower layer of the modern black soil, in recesses up to 15–20 cm, which resembled the body contours), many of the burials were likely covered by an earthen embankment that has not been preserved. The skeletons were on their backs in a straightened position, their hands mostly folded on the chests. Single burials are the most numerous, but also double burials are found with a man and a woman buried at the same time. In the late period of the culture, burials in stone tombs (cists) appeared.

The artefacts consisted of moulded dishes, which were placed around the head or less often near the feet or on the side, as well as jewellery and toiletries. For the late period, stone war hammers are known. Although the Vysotska culture is usually attributed to the Central European Urnfield culture, it differs by a strong military focus. The carriers of the culture had well-established trade relations with distant western centres of metalworking. (*23*, *126*)

Petrykiv cemetery *(Петриківський могильник)*

The Petrykiv cemetery was found in 1987 by O. Sytnyk in Petrykiv village, which is a suburban area of Ternopil city. In 1995 the excavations were started. The cemetery is located in the eastern part of the Ternopil Plateau of Western Podillia, on the flat terrain of the right bank of the Seret river – the left tributary of the Dniester river – on the southeastern slopes. Fragments of ceramics, sometimes metal products, flint production waste are found throughout the territory. A total of 148 burials were found in the cemetery. The inhumation, cremation and reburial of individual bones and cenotaphs is described. (*127*)

Location: 49.534488 N, 25.579807 E. Petrykiv village, Ternopil district, Ternopil region, Ukraine.

Excavations: 1995–1996.

Excavation authors: Mykola Bandrivskyi, I. Krypyakevich Institute of Ukrainian Studies of the National Academy of Sciences of Ukraine, Lviv, Ukraine

Storage of anthropological materials: I. Krypyakevich Institute of Ukrainian Studies of the National Academy of Sciences of Ukraine, Lviv, Ukraine

Description of samples:

Three samples were taken for aDNA analysis.

UKR170. *Petrykiv, burial 35.* Excavated in 1995. The burial was found at a depth of 0.35–0.45 m. The size of the grave pit was 2.10 x 0.5 m. The skeleton was straightened with the head oriented to the south. The length of the skeleton was 1.77 m. The skull lay on the left temporal and facial bones. The cranial sutures were separated. Half of the lower jaw was lost. From the preserved molars and premolars, it can be judged that the individual was 18–20 years old at the time of death. The arms were folded on the chest. The legs were straightened, ankles lying next to each other, but the phalanges were absent. On the left side of the pelvic bones at a depth of 0.25 m lay a large fragment of a pot. Chronology according to archaeology: 13–9 c. BCE.

UKR171. *Petrykiv, burial 58.* Double burial. Excavated in 1996. The grave at a depth of 0.40 m contained remains of two skeletons, with their heads oriented to the south. Almost all bones were displaced from their original positions. Based on the placement, size, and other features of the preserved bones, we can assume that the main burial (UKR171) belonged to a man with very large bones, who laid on his back with straight legs. The skull was badly destroyed, the lower jaw was pushed 20 cm to the side. Only fragments of ribs and other bones were also preserved. On the left side, close to the man's skeleton lay another, but much smaller and with a more delicate and feminine bone structure. Only the lower limbs including the ankle-foot joints, a part of the skull, the lower jaw and the bones of the forearms were preserved. Bones had intensely coloured bright green stains from bronze ornaments. At a depth of 0.40 m near the place where the frontal parts of the man's and woman's skulls touched, a small bronze ring was found. No other artefacts were found, except for small and probably redeposited fragments of pottery. Chronology according to archaeology: 13–9 c. BCE. Chronology according to ^{14}C dating: 1,278–1,055 cal BCE (2967±30BP).

UKR174. *Petrykiv, burial 79.* Excavated in 1996. The burial was found at a depth of 0.3–0.5 m. It was accompanied by six vessels and an animal skull. Only the lower part of the legs and the skull were preserved. Considering the position of the bones, the head was initially oriented to the south but later the skull was moved lower to the right knee joint. The lower jaw was absent. The placement of the foot bones proves that they both faced east. It is also interesting that a large fragment of a pot lay between the heel bones of the ankle joints. A tulip-shaped pot, a bowl and two ladles were found in the burial. Chronology according to archaeology – 13–9 c. BCE. Chronology according to ^{14}C dating: 359–104 cal BCE (2169±29BP). Based on the ^{14}C date and the genetic profile difference, this individual was separated from other Vysotska culture individuals in analyses.

Syncretic Ulvivok-Rovantsi cultural group: Lusatian culture, Vysotska culture (Ульвівецько-Рованцівська культурна група: Лужицька культура, Висоцька культура)

(Archaeology, text – D. Pavliv)

Syncretic Ulvivok-Rovantsi cultural group (Lusatian culture, Vysotska culture) formed on the border of the 2nd and 1st millennium BCE (from 10th c. to the first part of 7th c. BCE) in the southwest of Volyn, in the contact zone of the Vysotska and Lusatian cultures.

Presumably, the population is related to the carriers of the Vysotska culture in western Ukraine and the Lusatian culture on territories west of the Bug up to Silesia. Certain features of the culture

point to continuity with the kurgan cultures of the Middle Bronze Age and the Urnfield culture of Central Europe of the end of the Bronze Age, in particular the Urnfield culture of the Northern Alpine and Middle Danube regions.

Ulvivok-Rovantsi landmarks are represented by ritual necropolises on the high banks of the rivers Styr, Chornoguzka, Western Bug and Solokia. They have peculiar features of the funeral rite – combination of different types of inhumations and cremation, separate burials of skulls – and contain original complexes of ceramics, including special forms of funeral vessels. At the late stage the Ulvivok-Rovantsi culture was strongly influenced by the Lusatian culture. (*128–134*)

Rovantsi cemetery *(Рованцівський могильник)*

The Rovantsi cemetery is located on the border of the Volyn Upland and Polissia, on the high left bank of the Styr River – the right tributary of the Pripyat River – near Lutsk, between the villages of Rovantsi and Boratyn. According to radiocarbon dating, the necropolis belongs to the second part of 9th to 8th c. BCE (Kyiv Radiocarbon Laboratory of the Institute of Geochemistry of the Science of the National Academy of Sciences of Ukraine, laboratory number Ki–9815). It is the easternmost landmark and the largest burial ground of the Ulvivok-Rovantsi group. The total area is approximately 2,000 square meters. The site was found by V. Shkoropad in 1986 and was studied in 1987, 1989–1990 by expedition led by Dmytro Pavliv.

The necropolis belongs to the ground type with a bi-ritual burial rite with a significant predominance of inhumation. In total, 80 inhumation burials arranged in rows stretched from east to west with heads oriented to the south, 12 cremation burials of three types, 16 separate burials of skulls, as well as pottery between the burials, were investigated at a depth of 0.4–0.9 m. Burials were accompanied by moulded dishes of various shapes: pots, bowls, mugs, jugs, special funerary ceramics. The pottery was decorated with complex ornaments. Some of the burials contained bronze items: temple pendants, diadems, breast ornaments, rings, wrist and ankle bracelets. (*135*, *136*)

Location: 50.725306N, 25.361439E. Boratyn and Rovantsi villages, Lutsk district, Volyn region.

Excavations: 1987, 1989–1990.

Excavation author: Dmytro Pavliv, Ivan Krypiakevych Institute of Ukrainian Studies of National Academy of Sciences of Ukraine, Lviv, Ukraine

Storage of anthropological materials: Institute of Archaeology, National Academy of Sciences of Ukraine, Kyiv, Ukraine.

Description of samples:

Three samples were taken for aDNA analysis.

UKR167. *Rovantsi, burial 9.* The southern part of the cemetery. The depth was 0.7–0.8 m. There was a skeleton that was osteologically estimated to be a man (but genetically female) aged 30–40 years. The body was straightened, the head oriented to the southwest. The skull was lying on the occipital bone, tilted to the left side. The arms were bent at the elbows and placed on the chest and the stomach. There were no artefacts. Chronology according to archaeology: 9th to 8th c. BCE.

UKR168. *Rovantsi, burial 44.* The south-eastern part of the cemetery. The depth was 0.6–0.7 m. The body was straightened, the head oriented to the south. The skull rested on the right temporal

bone. The arms were bent at the elbows, the forearm of the right hand was on the stomach, the left hand was raised to the skull. There were no artefacts. Chronology according to archaeology: 9th to 8th c. BCE.

UKR169. *Rovantsi, burial 69.* The southern part of the cemetery. The depth was 0.6–0.7 m. The body was straightened, the head oriented to the south. The skull was lying on the occipital bone. The arms were bent at the elbows, the forearm of the right hand was on the chest, the left hand was on the stomach. There were no artefacts. Chronology according to archaeology: 9th to 8th c. BCE.

EARLY IRON AGE

Cimmerian culture (Кіммерійська культура)

(Archaeology – I. Shramko; text – I. Shramko, S. Zadnikov)

The Cimmerian culture (10th–8th c. BCE) landmarks in Ukraine are represented exclusively by inlet burials in Bronze Age kurgans. Not many artefacts have been found: mostly weapons, parts of a horse's bridle, work tools, pottery. The Cimmerians are the first people in the Northern Black Sea region known from written sources. They were nomads, armed horsemen who fought on the territory of Asia Minor, mentioned in cuneiform texts.

Kumy *(Куми)*

The kurgan near the village of Kumy was discovered in 2010 during an archaeological survey (*137*). The site with kurgans is located in the fields of the Krasnograd Research Station, near the village of Kumy, Krasnograd District, Kharkiv Region. The kurgan, located on the edge of the watershed plateau between the right tributaries of the Berestova river (right tributary of the Orel river), has nine mounds of various sizes, most of which have been ploughed and are barely visible on the surface of the field. (*138*, *139*)

Location: 49.3246500 N, 35.3687167 E. Kumy village, Krasnograd district, Kharkiv region, Ukraine.

Excavations: 2010.

Excavation authors: Iryna Shramko, Museum of Archaeology of V.N. Karazin Kharkiv National University.

Storage of anthropological materials: Museum of Archaeology of V.N. Karazin Kharkiv National University.

Description of samples:

One sample from the Early Iron Age was taken for aDNA analysis.

UKR066. *Kumy, kurgan 6, burial 5.* The inlet burial of a Cimmerian nomad. The burial was found at a depth of 0.7–0.9 m above the level of the reference point. The contours of the pit were not traced. The skeleton of an adult man (35–36 years old) was lying on his right side, with his head to the west. The bones of the right hand were slightly bent at the elbow and were stretched along the body. The left hand was missing. There were no artefacts. Chronology according to archaeology: 10th–9th c. BCE. Chronology according to ^{14}C dating: 1195–919 cal BCE (2865±39BP).

Thracian Hallstatt (Thraco-Cimmerian) culture *(Фракійський Гальштат)*

(Archaeology, text – I. Bruyako)

The Thraco-Cimmerian culture still does not have the status of a distinct archaeological culture in historiography. It was described in 1920–1930 based on horse ammunition items from hoards belonging to the late Urnfield and early Hallstatt period in Central and Eastern Europe. The Kartal III burial ground is the only complete monument of Thracian-Cimmerian culture to date. (*140*)

Kartal *(Картал)*

The Kartal settlement is a multi-layered archaeological site, located on the left bank of the Lower Danube, 1.5 km east of the village Orlovka. For this study, the materials from the burial ground of the third cultural horizon (Kartal III) were used. This horizon dates to the Middle Hallstatt period around 9th to 8th c. BCE. A total of 530 graves were excavated (about half of the entire cemetery). The features of the artefacts and the burial rite indicate that Kartal III belongs to the Thracian-Cimmerian culture. The population of the settlement consisted of two main ethnocultural components – Thracian and Iranian. Possibly, they represented the tribal world of the Western (South-Western) Balkans (Illyrians, Paeonians, Dardanians), as well as the local people as a relic of the Indo-Aryan Late Bronze Age community (Zrubna-Sabatynivska and Bilozerska cultures). (*141*)

Location: 45.319997 N 28.411989 E. Orlivka village, Izmail district, Odesa region.

Excavations: 2005, 2007, 2008.

Excavation authors: Igor Bruyako, South Ukrainian K. D. Ushinsky National Pedagogical University State Institution, Odesa.

Storage of anthropological materials: Odesa Archaeological Museum.

Description of samples:

Seven samples were taken for aDNA analysis, DNA was successfully extracted from six.

UKR000. *Kartal, burial 132.* The burial pit was not traced. The skull stood at a depth of 60 cm. The skeleton lay in a crouched position on its right side, with the head oriented to the south. The arms were bent at the elbows. Behind the head there was a lower jaw of small cattle. Opposite the front part of the skull there was a small black-clay jug. Chronology according to archaeology: 9th to 8th c. BCE. Chronology according to ^{14}C dating: 900–798 cal BCE (2676±30 BP).

UKR001. *Kartal, burial 124.* The shape of the pit was close to rectangular. The depth of the contours of the pit was 75 cm, the size 210x110x15 cm. The skeleton was fragmented, the position crouched, on the right side, head oriented to the south. In front of the skull there was a black-clay cup-shaped vessel. Chronology according to archaeology: 9th to 8th c. BCE.

UKR002. *Kartal, burial 19.* The rectangular pit with strongly rounded corners was oriented along the NNW-SSE line. The contour of the pit was at the level of 80 cm. The length of the pit was 215–217 cm, the width in the middle 110 cm, the depth 25–30 cm. The skeleton lay in a crouched position on the right side, head oriented to SSE. The arm bones were bent at the elbows, the right hand was brought under the skull. There was a black polished goblet near the left hand, with its

neck facing the front of the skull. Under the goblet an iron knife with a curved back and a straight blade lay. Chronology according to archaeology: 9th to 8th c. BCE.

UKR005. *Kartal, burial 103.* The pit has the shape of a wide rectangle with rounded corners, oriented along the N-S line. At a depth of 25–30 cm a human skull was found. Further, the contours of the burial pit were fully revealed. The size of the pit was 1.7 x 1.15–1.2 m, the depth 45 cm. The skeleton lay on the back. The legs were bent at the knees, arms at the elbows. The hands were crossed on the chest. Body orientation to SSE. Near the skull there was a moulded rhyton-shaped vessel. A bronze item was found under the lower thoracic vertebrae – the tip of a belt in the form of an elongated conical cylinder. On the chest lay a fragment of a polished bowl, decorated with flutes along the edge and inner surface. Chronology according to archaeology: 9th to 8th c. BCE.

UKR006. *Kartal, burial 109.* The contour of a very large pit had the shape of a rectangle and was found at a depth of 100–110 cm. Orientation on the SE-NW line. The size of the pit was 260x120–130 cm, depth up to 30 cm. At the centre of the pit's bottom, a trough-like depression was clearly visible, in which the skeleton was located. The skeleton was poorly preserved. The skull was crushed, the bones of the limbs were not completely preserved. The skeleton was in a crouched position and lay on the right side, with the head oriented to SSE. In front of the skull there was a low black-clay vessel (bowl) and a bronze fibula. Very small (1–1.5 mm) white beads were found around the 4th to 5th cervical vertebrae. Between the left shoulder and the back of the skull there was a small bronze plaque with a loop on the back. Chronology according to archaeology: 9th to 8th c. BCE.

UKR007. *Kartal, burial 126.* The burial pit has not been traced. The skeleton lay at a depth of 60–75(80) cm. The skeleton was in a crouched position and lay on the right side, with the head oriented to SSE. The arms were bent at the elbows, the hands directed to the chin. The artefacts consisted of a small black-clay vessel located near the skull. Chronology according to archaeology: 9th to 8th c. BCE. Chronology according to ^{14}C dating 996–831 cal BCE (2767±29 BP).

<u>Scythian culture</u> *(Скіфська культура)*

(Text – I. Shramko, S. Zadnikov)

On the territory of Ukraine, Scythian culture was widespread in the forest-steppe and steppe zones of the Northern Black Sea Region in the 7th–4th c. BCE. On the Dnipro left bank forest-steppe, the Scythians appear in the second half and last quarter of the 7th c. BCE. The Scythian tribes, their daily life and traditions were described by the ancient Greek historian Herodotus in the middle of the 5th c. BCE.

The landmarks of Archaic Scythian period include kurgans, the burial artefacts of which show signs of the early Scythian culture complex: certain types of weapons, horse bridles, items made in the Scythian "animal style", as well as imported items of Egyptian or West Asian origin, brought by nomads who moved to the forest-steppe through the Caucasus. One of the largest Scythian kurgan necropolises of the early period was found in the basin of the Sula river (the left tributary of the Dnipro). Large kurgans were studied in the basin of the Vorskla river (left tributary of the Dnipro) and Sukha Grun river (tributary of the Psel river), as well as in the Siversky Donets river basin.

From the last quarter of the 6th c. BCE the presence of new nomadic groups, which advanced to the forest-steppe area from the east and through the Caucasus, is recorded. A change in the material

culture occurred in the burials of that time, *e.g.*, antique dishes became more common. Settled population groups were moving from western forest-steppe regions, occupying new territories of Dnipro-Donetsk area and populating the eastern regions of the Ukrainian forest-steppe. Plenty of new unfortified settlements arose. Archaic Scythia was replaced by Classical Scythia. This period is well known from the cultural artefacts of numerous kurgans and settlements of the 5th–4th c. BCE.

The Scythians were establishing relations with the local settled agricultural population, as well as with other contemporary ancient centres since the Archaic period. (*142*)

Scythian period. Forest-steppe zone of the right bank of Dnipro (Правобережний лісостеп)

(Archaeology - H. Kovpanenko, B. Levchenko; text – D. Grechko)

In Scythian times, the forest-steppe zone of the right bank of Dnipro was densely populated by a settled population of local origin (*143*). The natural conditions of the region were favourable for various groups of nomads and semi-nomads that appeared there in the 7th–4th c. BCE (*14*, *144*). In the second half of the 6th c. BCE, changes in climate and the accompanying shift of natural zones caused the southward migration of forest tribes that left monuments of the Podhirtsiv type (*145*). For these reasons, Porossia (area of the Ros River basin, southward from Kyiv) became a place of active population movement and close interaction between different cultural groups.

Early Scythian kurgans in the Ros River basin have attracted the attention of Scythologists for a long time. Most of the burials were investigated before 1917. Excavations of the Medvin necropolis were carried out in the 1970s and 1980s (*146*, *147*).

Medvyn *(Медвин)*

There are several kurgan groups near the village Medvyn, Boguslav district, Kyiv region, in the Girchakiv forest tract. They are located on the elevated right bank of the Khorobra river (a tributary of the Ros river, a right tributary of the Dnipro). This necropolis belonged to the forest-steppe agricultural population, which preserved archaic burial traditions (decarnation through exposure to the elements and scavangers) (*148*). The time range when the necropolises were used can be attributed to the Zhabotyn period and the beginning of the early Scythian period (second half of the 8th until third quarter of the 7th c. BCE) (*147*).

The burial rite of the Medvyn necropolis has direct analogies in the burial ground of the early Zhabotyn period near the village of Tyutky in the Southern Bug basin, where these traditions have no local basis (*149*). Burials with a similar set of artefacts are found in the earlier dated kurgans of Saharna-1 burial ground (Cigleu) in forest-steppe Moldova (*150*). These facts allow to assume the movement of the population from Middle Transnistria (the oldest complexes) through Pobuzhzhia (Tyutky, Nemyriv, Vyshenka-2) to Porossia in the early Zhabotyn period. The migrants moved into regions sparsely populated by people of the late Chernolis culture, where mixing of different ethno-cultural groups occurred. The funeral rite and the set of moulded dishes indicate either the participation of the Chornolis-Zhabotyn population of Porossia in the genesis of this population, or the influence of migrants on the material culture (*151*).

Location: 49.40542 N, 30.846856 E. Medvin village, Bila Tserkva district, Kyiv region.

Excavations: 1973, 1984.

Excavation authors: Halina Kovpanenko, Institute of Archaeology, National Academy of Sciences of Ukraine, Kyiv; Borys Levchenko, Communal institution "Museum of History of Boguslav Region" of Boguslav City Council, Kyiv Region.

Storage of anthropological materials: Institute of Archaeology, National Academy of Sciences of Ukraine, Kyiv.

Description of samples:

Ten samples were taken for aDNA analysis, seven yielded a sufficient amount of DNA for further study.

UKR036. *Medvyn, tract Girchakiv Lis, Group I, kurgan 1, burial 1.* The burial was in a ground pit with a latitudinal orientation. The skeleton was stretched out on its back, the head was oriented to the northwest. The burial was accompanied by artefacts similar to the Hryshkov sets – a quiver set, a spear, meat food (*152*). Chronology according to archaeology: second half of the 5th c. BCE. ^{14}C calibrated date 773–426 cal BCE (2481±31 BP).

UKR042. *Medvyn, tract Girchakiv Lis, Group I, kurgan 3/1973, burial 1.* The burial was in a rectangular ground pit with a small dromos entrance on the south side. The wooden floor was damaged during the secondary penetration to the grave. The bones of a 35–40-year-old man and a 25–30-year-old woman (UKR042) lay in two elongated parallel clusters with a certain system. Several moulded vessels were found *in situ* near the bones. The southern part of this composition was disturbed because of the secondary penetration into the grave for ritual manipulation. The burial artefacts included a set of moulded dishes (a bowl, three cups and a ladle), bronze and bone arrowheads, mushroom-shaped bone parts of a quiver, necklaces of cowrie shells and opaque glass, and an iron knife. (*146*). Chronology according to archaeology: second half of 7th c. BCE. Chronology according to ^{14}C dating: 779–539 cal BCE (2503±30 BP).

UKR039. *Medvyn, tract Girchakiv Lis, Group II, kurgan 3/1973, burial 1.* The burial was in a rectangular ground pit with a size of 2.9x1.95 m, 0.6 m deep, with a small dromos entrance on the southern side and two wooden pillars near it. The roof was not preserved. The filling was very dense, indicating secondary penetration. The burial was probably disturbed for a ritual purpose, as the long bones were lying chaotically in the central part of the grave and the skull was closer to the southern wall. Near the skull, pointed to the south, lay an iron spearhead. (*146*). Chronology according to archaeology: second half of 7th c. BCE.

UKR035A, UKR035B, UKR043, UKR044. *Medvyn, kurgan 22, field numbers 403, 405, 406.* The burial was in a rectangular pit with a dromos on the south side. In ancient times, the pit was covered with longitudinal and transverse oak logs. Only fragments remain from the ceiling, what indicates a secondary entry into the grave. Judging by the number of skulls, the remains of at least ten individuals (including UKR035, UKR043, UKR044) were placed in the central part of the pit. The bones of nine skeletons were laid out in a certain system, and the skulls were placed near the northern and southern edges. The bones of the "western" and "eastern" skeletons were partially laid out in pseudo-anatomical order. These are completely excarnated secondary burials, buried after the complete loss of ligaments and consist of separate bones. The remains of the dismembered individual, who was buried last, are of interest. It includes the bones of the arm, leg, lumbar spine and pelvis, which were not in the correct anatomical placement. Judging by the destruction of the roof and the presence of bowls above the level of the grave's bottom, there was a secondary penetration to the grave for the reburial of the dismembered individual. Hence, the burial complex

of kurgan No. 22 was a two-act burial (secondary burials with reburial). Some of the bones had traces of cutting tools and teeth of predators. During the reburial of the dismembered skeleton, the entire central part of the chamber was opened. The bones of the ancestors were collected in a "pack" or a pile, and parts of the dismembered body were placed next to or together with it. It was difficult to determine who was buried, it can be assumed that it was a woman. The burial was accompanied by various artefacts. In the south-eastern corner of the pit, near the dromos, three large bowls were found. One of them was lying upside down. Another was above the dismembered skeleton, 35 cm above the bottom, with a ladle with a handle inside. The third one was 50 cm above the bottom, a little away from the others. Next to one skull there was a moulded cup, an iron knife, a spinning wheel, and scattered necklaces. A bronze pendant was found near the northwestern edge of the bone remains. Chronology according to archaeology: third quarter of the 7th c. BCE.

Scythian period. Forest-steppe zone of the left bank of Dnipro, Vorskla group of landmarks (Лівобережний лісостеп, Ворсклинська група пам'яток)

(Text – S. Zadnikov, I. Shramko)

The Scythian culture of the Vorskla group of landmarks is represented by unfortified settlements, hillforts, kurgans, and ground burials. It was formed in pre-Scythian times, in the second half to the end of the 8th c. BCE, when the Basarab tribes – carriers of the Middle Hallstatt culture – moved from the right to the left bank of the Dnipro River, to the basin of the Vorskla River and some tributaries of the Psel River. According to written sources, the tribes of the Neurs, Budins and Gelons lived in this territory in the second half of the 6th to 5th c. BCE and the city of Gelon existed at the last quarter of the 6th to 4th c. BCE. According to archaeological data, the territory of the Vorskla basin was populated by settled tribes, who were engaged in agriculture, cattle breeding and crafts. From the third quarter of the 7th c. BCE the population was in trade relations with the ancient centres of the Northern Black Sea region. From the end of the 8th to the end of the 6th c. BCE local people had tight connections with the Illirian-Thracian tribes of the Northern Balkans, Central Europe, the right bank of the Dnipro and the Scythians, who moved into the region in several waves from the second half to the end of the 7th c. BCE. Burial traditions have regional characteristics, reflecting the diversity of society and complex social composition of the population. (*153–157*).

Bilsk fortified settlement (Bilsk Horodische, Bilsk Hillfort) *(Більське городище)*

(Archaeology – B. Shramko, I. Shramko, S. Zadnikov; text – I. Shramko, S. Zadnikov)

Bilsk fortified settlement (8th–4th centuries BCE) is known as the largest fortified settlement of the Early Iron Age in Europe and as a major craft, trade, religious and political centre of forest-steppe Scythia. Most researchers identify it with the city of Gelon described in Herodotus' "History". It is located in the forest-steppe area on the left bank of the Dnipro river, on the watershed plateau between the rivers Vorskla (tributary of the Dnipro) and Sukha Grun (tributary of the Psel).

The settlement consists of three fortifications connected via an earthen rampart about 35 km in length and occupies an area of 5,000 hectares. There are kurgan necropolises westward of the settlement, in the locations of Skorobir, Osnyagi, Peremirky, Pereshchepyne, and Marchenki.

Small groups of kurgans dated to the later period are located within the settlement. On its territory and beyond, separate earthen burials and a large earthen necropolis have been found.

Some unfortified satellite settlements were discovered around the main settlement. The earliest of those were founded in the second half to the end of the 8th c. BCE by migrants from the right bank of the Dnipro, who were carriers of the Basarab archaeological culture. Until the end of the 6th c. BCE, the population maintained contacts with the Hallstatt cultures.

The first excavations were carried out in 1906 by V. O. Gorodtsov. Since 1958 the hillfort and necropolises have been consistently studied by Ukrainian researchers from Kharkiv, Poltava, Donetsk, Kyiv, as well as researchers from Germany. In addition to knowledge of the rich material culture and extensive trade relations of the Scythians with other cultural centres, the complex social and ethnic composition of the population has been traced. The diversity of the society is recorded, first of all by the funeral rite, the presence of graves of representatives of the local aristocracy, elite women's burials, etc. (*154*, *158–166*)

Location: 50.093324 N, 34.596018 E. Bilsk village, Poltava district, Poltava region.

Excavations: 1975, 1978–1980, 1983, 1987–1988, 1990, 1994, 2009, 2011–2021.

Excavation authors: Borys Shramko, Iryna Shramko, Stanislav Zadnikov, V.N. Karazin Kharkiv National University, Kharkiv.

Storage of anthropological materials: Museum of Archaeology of V.N. Karazin Kharkiv National University.

Description of samples:

17 samples were taken for aDNA analysis, seven samples yielded a sufficient amount of DNA for further study.

UKR078. *Bilsk, Western fortification, ash hill 10, field number 7675, square 21–22/Щ–Э, depth 0.60 m, pit 12.* Excavated in 2011. The burial has features of the Illirian-Thracian basis. The pit was found at a depth of 0.50 m, its bottom was at the depth of 1 m from the modern level. The diameter of the pit was 2.45 x 2.70 m. Only a human skull was found in the ashy filling, as well as fragments of moulded ware, a fragment of the wall of a Greek amphora with two red stripes (2nd–3rd quarters of 6th c. BCE), two bronze arrowheads (three blade, one basic), one iron three-bladed arrowhead, a fragment of a bone cheek-piece designed in an animal style, two fragments of iron bracelets. Chronology according to archaeology: the middle of 6th c. BCE. Chronology according to ^{14}C: 755–408 cal BCE (2445±37 BP).

UKR083. *Bilsk, Eastern fortification, excavation 32, field number 205/32-1988, square Я-14, depth 0.50 m., household pit 8.* Excavated in 1988. Supposedly, the individual belonged to the local agricultural population. The pit was found at a depth of 0.20 m, its bottom was at the depth of 0.8 m. It was of a round shape with a diameter of 2.33 m. There were fragments of moulded ware, a bronze bracelet made of thin wire, four clay cone-shaped sinkers in the black soil filling, as well as fragments of a human skull. Chronology according to archaeology: second half of the 5th–4th c. BCE.

UKR087. *Bilsk, Skorobir kurgan cemetery, kurgan 1/2013, burial 1.* Excavated in 2013. The individual belonged to the local elite, the burial has features of the Illirian-Thracian basis. The kurgan had a height of 0.2 m and a diameter of 19 m. Annual ploughing practically levelled the kurgan – the kurgan had almost no external signs. From the southwest (at a depth of 1.55 from the

rapper), the burial chamber was adjoined by a dromos on which the burial of a man lying on his back with his head oriented to the west was found. A bronze arrowhead was lodged in the distal part of his left tibia. The burial was built as a wooden crypt. Remains of wooden planks can be traced along the walls of the burial chamber and in its filling. On the chamber's ceiling there were three sets of horse bridles, which included iron bits, cheek-pieces, bronze clips and zoomorphically decorated plaques. The main burial was looted, but some items (mainly in the southern part of the chamber) remained *in situ*. At the bottom of the southern corner there was a Greek amphora, in the southwestern corner a jar. Along the western wall of the burial chamber there were a 2.27 m long iron spear and several sets of horse bridles (iron bits with cheek-pieces). At 0.2–0.3 m to the east, there were remains of a leather quiver with 147 bronze tips. In the central part, at the bottom of the burial, a part of the skeleton and the skull of a goat were found. A row of beads was found in the eastern part. Fragments of a bronze pin and an iron knife were also found in mixed filling. Not far from the western wall, separate bones and skull fragments of a man and a woman (UKR087) were piled up. A gold plaque 1x1 cm was found in the man's jaw. Thus, the main burial was a pair (man, woman) and belonged to the elite. Chronology according to archaeology: last quarter of the 6th c. BCE.

UKR088. *Bilsk, Skorobir kurgan cemetery, kurgan 19.* Excavated in 1975. Supposedly, the buried individual belonged to the local elite. The kurgan was looted. The height was 0.8 m, the diameter about 29 m. The rectangular grave pit with dimensions of 2.5x3.3 m was elongated along the SW-NE line. On the south-western side there was an entrance corridor with a width of 1.1 m and a length of 3 m. The floor of the corridor was sloping. The bottom of the grave was at a depth of 1.8 m from the top of the kurgan. The grave was built as a wooden crypt with four main pillars and two small additional pillars at the entrance. The walls and floor were lined with wood. The skeleton was badly damaged. Among the bone remains there were a cranial lid, fragments of the tubular bones of a man's limbs, a human shoulder blade. Fragments of the lower and upper jaw of a woman were found near the south-eastern wall (UKR088). The finds included an iron knife with a bone handle, pottery fragments, remains of sacrificial food (large bones of a goose), a gold plaque shaped as a stylized hare. Plaques of this type as well as knives with a bone handle were widespread in kurgans of the 4th c. BCE. Chronology according to archaeology: 4th c. BCE. Chronology according ^{14}C dating: 761–420 cal BCE (2467±28BP).

UKR089. *Bilsk, Skorobir kurgan cemetery, kurgan 6.* Excavated in 1979. Supposedly, the buried individual belonged to the local elite. The kurgan was looted. The height of the kurgan was 1.3 m, diameters were 46 m and 42 m. The burial was in a grave pit with dimensions of 3.1x2.2 m. The bottom of the pit was at a depth of 2.3 m from the top of the kurgan. The grave was built as a wooden crypt, made of birch logs 15–20 cm thick, covered with a wooden roof, which was badly damaged by robbers. The bottom of the grave was lined with birch bark. The kurgan was robbed twice. The human skeleton was badly damaged, the bone remains scattered. It was probably oriented along the SW-NE line. The remains of a skull were found in the southwest corner of the grave, and the tubular bones of the legs lay near the northern wall. Fragments of narrow bronze plates from a belt, remains of an iron bit and an iron awl were found. Chronology according to archaeology: 5th–4th c. BCE.

UKR090. *Bilsk, Skorobir kurgan cemetery, kurgan 20.* Excavated in 1975. Supposedly, the buried individual belonged to the local middle-level elite. The kurgan was looted. The height was 1.2 m, diameter 28 m. At a depth of 0.85 m a robbery passage was seen. The grave pit had dimensions of 3.5x4.0 m. The pit was stretched out along NW-SE line and covered with logs. The bottom was at

a depth of 2.65 m from the top of the kurgan. The grave had a wooden floor. Most of the human bones were destroyed or thrown into the robbery passage. Only a fragment of the lower jaw of an adult was found near the NE wall of the grave. Among the things scattered around the grave were fragments of a moulded pot, an amphora, a fragment of a bowl and a fragment of a Greek black-glazed vessel of the 4th c. BCE, which determines the chronology according to archaeology: 4th c. BCE.

UKR091. *Bilsk, Pereshchepyne kurgan cemetery, kurgan 3, burial 2.* An inlet burial. Excavated in 1980. Supposedly, the buried individual belonged to the local high-level elite. The height of the kurgan was 0.9 m. The kurgan contained two chronologically close burials, main (1) and additional (2). Burial 2 (UKR091) belonged to a 20–30-year-old man and had rich grave goods. The pit of this burial had the shape of an irregular rhombus with slightly rounded corners (2.9x3.1x2.55x2.75 m), with a depth of 1.3 m. Along the north-eastern wall, a stretched skeleton was laid on the back, with the head oriented to N-NW. The bones were in the correct anatomical order, but almost completely decayed. The remains of the skull and teeth were better preserved, which made it possible to establish the age at death. The artefacts included an iron sword with a golden scabbard, two spears with iron pointed tips and iron shafts. To the northwest of the head of the skeleton there were the remains of a quiver with arrows that had bronze sleeve tips. A total of 57 bronze arrowheads and 5 of their fragments were found. Also, an iron knife, lekythos, Greek ceramic jugs were found in the burial. Chronology according to archaeology: end of 5th until beginning of 4th c. BCE.

Kolomak *(Коломак)*

(Archaeology – V. Radzievska; text – S. Zadnikov, I. Shramko)

The settlement is located on the cape of an unnamed tributary of the Kolomak River (a tributary of the Vorskla River). The settlement consisted of the main yard (5.4 hectares) and suburb area (8 hectares). Excavations revealed that it was inhabited by the local forest-steppe population of the Scythian period (the second half of the 6th to 4th c. BCE). The territory of the settlement was densely populated. During excavations, 14 dwellings, numerous pits and a well were found. A big number of objects of material culture were found, namely knives, sickles, hoes, arrowheads, clay figurines, fragments of local moulded ware, fragments of Greek amphorae. Features of attacks were traced. The first attack dates back to the 6th–5th c. BCE, when the settlement suffered from the raids of the steppe Scythians. After that it was restored and strengthened with an additional rampart and ditch on the eastern side. In the second half of the 5th c. BCE the territory of the settlement decreased. At the border of 4th to 3rd c. BCE the settlement was again defeated by nomads and ceased to exist. (*167–170*)

Location: 49.864876 N, 35.277719 E. Kolomak city, Kharkiv region, Ukraine. The headland on the left bank of a tributary of the Kolomak River, which is a left tributary of the Vorskla River.

Excavations: 1987, 1991.

Excavation authors: Vira E. Radzievska, Museum of Archaeology of V. N. Karazin Kharkiv National University, Kharkiv.

Storage of anthropological materials: Museum of Archaeology of V.N. Karazin Kharkiv National University, Kharkiv.

Description of samples:

Five samples were selected, DNA was extracted from two of them.

UKR095. *Kolomak, Field number 1198/IV-87, pit 51 (fragment of the skull cap of an adult, skull 3).* The burial has been attributed to local agricultural tribes. Pit 51 was found at a depth of 0.40 m from the modern level, size 3.10x3.20 m. The depth of the bottom gradually increased from 0.80 m in the western part to 1.10 m in the eastern part, 1.15 m in the southwest. A large amount of household waste typical for this period was recorded: fragments of moulded ceramics (over 200 specimens), quartzite chips (5 pieces), clay plaster (5 pieces). In the garbage filling of the pit, starting from 0.60 m, scattered human bones were found. Separate parts of at least 4 skeletons (including a tooth – 1198/IV-87, UKR095) and one relatively complete skeleton of a subadult without traces of a ritual were found. All human bones were of poor preservation. Chronology according to archeology: 6th–4th/3rd c. BCE. Chronology according to ^{14}C dating: 389–204 cal BCE (2241±27 BP).

UKR096. *Kolomak, Field number 1915/IV-88; square БД-3.* A human lower jaw was found in the cultural layer of the settlement, in excavation 4, at a depth of 0.40 m, among cultural remains attributed to local agricultural tribes. Chronology according to archaeology: 6th–4th/3rd c. BCE. Chronology according to ^{14}C dating: 382–199 cal BCE (2220±25BP).

Kupievakha *(Куп'єваха)*

(Archaeology – S. Berestnev; text – I. Shramko, S. Zadnikov)

A group of 76 kurgans is located on the watershed plateau on the right bank of the Berezivka River (right tributary of the Vorskla River). (*153–155*, *157*, *171*)

Location: 50.207055 N, 35.285673 E. Kupevaha village, Bohodukhiv district, Kharkiv region, Ukraine.

Excavations: 1980, 1992–1993, 2003.

Excavation authors: Serhii Berestnev, V.N. Karazin Kharkiv National University, Kharkiv.

Storage of anthropological materials: Museum of Archaeology of V.N. Karazin Kharkiv National University, Kharkiv.

Description of samples:

Two samples were taken from the burials, DNA was extracted from one of them. Since Kupievakha is located very close to the Siversky Donets basin and this sample is the only nomad sample from the left bank of Dnipro region, it is grouped together with Siversky Donets nomads in analyses.

UKR105. *Kupyevaha, kurgan 23, burial 1.* The burial has been attributed to nomads. The primary grave. The pit is square-shaped (3.5x3.6 m), its corners oriented to NE-SW and NW-SE directions. The bottom was at a depth of 2.6 m. On the bottom, along the western wall, lay the skeleton of a child. It was stretched out on the back with the head oriented to the south. The remains of the buried adult individual (UKR105) had been disturbed by robbers: closer to the south-western wall, the parietal part of the skull and individual bones of the limbs were found out of anatomical order. Beads (carnelian, amber, glass?), a small gold plaque of a hemispherical shape with an internal loop, fragments of iron items (a knife) and a Greek amphora made on the island Lesbos were found

in the burial. Chronology according to archaeology: the end of 6th to beginning of 5th c. BCE. Chronology according to ^{14}C dating cal. 798–552 cal BCE (2547±26BP).

Scythian period. Forest-steppe zone of Dnipro-Donets, Siversky Donets group of landmarks (Дніпро-Донецький лісостеп, Сіверськодонецька група пам'яток)

(Text – I. Shramko, S. Zadnikov)

The Scythian culture of the Siversky Donets group of landmarks is represented by unfortified settlements, hillforts and kurgans. Until the second half of the 6th c. BCE, the territory was occupied only by nomads, whereas later numerous settlements of agricultural tribes appeared there. The region was settled by migrants.

The graveyards formed compact groups. The main type of burials of the Siversky Donets group of landmarks were ground pits with wooden floor and crypts. Individuals were buried stretched out on the back. Southern orientation of the head prevailed. The ritual use of fire was widespread. Child burials in kurgans were almost completely absent. Along with the burials of the main population of the region in the Scythian period, the graves of other ethnic groups have also been traced.

Tribes that inhabited the Dnipro-Donets forest-steppe zone in Scythian times are classified as Herodotus' Budins, whose tribal union included the Melankhlens tribe. The local features of the region were stable from the second half of the 6th to the 5th c. BCE and began to change due to external influence at the end of the 5th and in 4th c. BCE. In the last quarter of the 4th c. BCE, it is possible to assume a Sauromatian-Sarmatian invasion in the region. (*172–177*).

Cheremushna *(Черемушна)*

(Archaeology – Yu. Buynov; text – I. Shramko, S. Zadnikov)

The kurgan burial consisting of about 150 kurgans is located on the edge of the plateau of the left bank of the Cheremushna River (the left tributary of the Mzha River). The burial arose in the Bronze Age and can be classified as medium-sized (group of 50–150 kurgans). Burials were made in ground pits and in inlet catacombs. Also, ritual burning was traced. (*178*)

Location: 49.854878 N, 35.818011 E. Cheremushna village, Bohoduhiv district, Kharkiv region.

Excavations: 2002.

Excavation author: Yurii Buynov, V. N. Karazin Kharkiv National University.

Storage of anthropological materials: Museum of Archaeology of V. N. Karazin Kharkiv National University, Kharkiv, Ukraine.

Description of samples:

One sample was taken for aDNA analysis.

UKR111. *Cheremushna, kurgan 10, burial 1.* A primary grave. The burial has been attributed to nomads. The height of the kurgan was 1.25 m. The burial pit was rectangular-shaped (3 x 2.1 m), its depth was 1.7 m. The burial was robbed. Human bones were found in the filling of the lower part of the chamber. At the skull base, traces of its cutting were visible, and on the inner side of the parietal bone there was a round hole, formed as a result of its attachment to a wooden pole.

The following artefacts were found in the burial: fragments of an iron knife, four bronze arrowheads, fragments of iron horse bits, two bronze plates from a horse's bridle, a bronze buckle, a fragment of a Greek amphora and a fragment of a silver fitting from a wooden vessel. Chronology according to archaeology: end of 5th – beginning of 4th c. BCE. Chronology according to ^{14}C dating: 775–540 cal. BCE (2496±26BP).

Karavan *(Караван)*

(Archaeology – V. Okatenko; text – I. Shramko, S. Zadnikov)

The necropolis consisting of four kurgans is located on a high plateau on the left bank of the Merefa River between the Karavan village and the Lyubotin city, Kharkiv region. (*172–174*, *177*, *179–181*)

Location: 49.925983 N, 35.880553 E. Karavan village, Lyubotyn territorial community, Kharkiv region.

Excavations: 2013.

Excavation authors: Vitalii Okatenko, State enterprise research centre "Security Archaeological Service of Ukraine" of the Institute of Archeology, National Academy of Sciences of Ukraine, Kyiv.

Storage of anthropological materials: Institute of Archaeology, National Academy of Sciences of Ukraine, Kyiv Ukraine.

Description of samples:

One sample was taken for aDNA analysis.

UKR116. *Karavan, kurgan 2, burial 2, (central grave (primary burial)).* The burial has been attributed to nomadic elite. The ground embankment was about 5 m in height and was completely destroyed during the excavation. The pit was 1.7 m deep from the ancient surface and had a rectangular shape measuring 4.8 x 4 m. The burial was robbed. In the burial, the upper part of a Greek amphora, eight gold plaques decorated with ornament, fragments of a silver rhyton decorated with a bull's head, a bronze dish with gilding, bronze arrowheads, fragments of an iron three-looped cheek-peice, and fragments of a moulded scoop were found. Chronology according to archaeology: the first quarter of 6th c. BCE. Chronology according to ^{14}C dating: 775–516 cal BCE (2491±28BP).

Mala Rogozianka *(Мала Рогозянка)*

(Archaeology – Y. Buynov; text – S. Zadnikov, I. Shramko)

The group of five kurgans is located 1 km to the north of the Mala Rogozianka village, on a high plateau on the left bank of the Udy River. The distance to the nearest settlements is several km. In terms of size, it belongs to the group of small necropolises. A moat was traced around the kurgan. There were several types of burials: crypts in a ground pit, wooden crypts with pillars and a dromos. Buried individuals were laid on their backs, with heads oriented to the southwest. The burials of nobility were accompanied by the dependent persons. (*172*).

Location: 50.13547 N, 35.909989 E. Mala Rogozianka village, Bohoduhiv district, Kharkiv region.

Excavations: 1989.

Excavation authors: Yurii Buynov, V. N. Karazin Kharkiv National University.

Storage of anthropological materials: Museum of Archaeology of V.N. Karazin Kharkiv National University.

Description of samples:

One sample was taken for aDNA analysis.

UKR113. *Mala Rogozianka, kurgan group I, kurgan 1, burial 3.* A primary grave. Attributed as nomadic. The height of the kurgan was 1–1.1 m. The pit was of rectangular shape (3 x 2.5 m), a wooden crypt was built in it. The skeleton was poorly preserved. It lay on the back with the head oriented to the west. Arrowheads (33 bronze, 4 bone, 1 iron) and a moulded pot were found. Chronology according to archaeology: the first quarter to first half of the 6th c. BCE.

Nyzhnia Gyivka *(Нижня Гиївка)*

(Archaeology – Y. Buinov; text – S. Zadnikov, I. Shramko)

The burial ground near Nyzhnia Gyivka village is located on the edge of the plateau on the right bank of the Merefa River. It consists of 83 kurgans and belongs to medium-sized burial grounds (50–150 kurgans) of the Siversky Donets group of landmarks. Burials of 4th c. BCE were made in ground pits with "shoulders" around the perimeter. Ritual burning has been detected. (*172–177*, *179*, *180*)

Location: 49.914637N, 35.935222 E. Nyzhnia Gyivka village, Lubotyn city, Kharkiv region.

Excavations: 1994.

Excavation authors: Yurii Buynov, V.N. Karazin Kharkiv National University, Kharkiv, Ukraine.

Storage of anthropological materials: Museum of Archaeology of V.N. Karazin Kharkiv National University, Kharkiv.

Description of samples:

Three samples were taken for aDNA analysis, two samples yielded a sufficient amount of DNA for further study.

UKR101. *Nyzhnia Gyivka, kurgan 3, burial 1.* An inlet burial. Attributed as nomadic. The height of the kurgan was 0.5 m. The burial was found at a depth of 0.85 m from the benchmark. The contours of the grave-pit were not traced. The skeleton lay stretched out on its back, with the head oriented to the south. There were bronze bracelets on the hands, a temporal ring near the skull, and 18 pyramidal pendants made of blue glass on the chest. Chronology according to archaeology: 4th c. BCE.

UKR114. *Nyzhnia Gyivka, kurgan 3, burial 2 (central grave (primary burial)).* The burial is attributed as nomadic. A rectangular burial pit 2.5 x 1.6 m with a depth of 1.8 m. The skeleton, poorly preserved, without a skull, was oriented with its head to the south. A bronze arrowhead was found near the shoulder. Chronology according to archaeology: the end of 5th – 4th c. BCE.

Gryshkivka *(Гришківка)*

(Archaeology – D. Grechko; text – S. Zadnikov, I. Shramko)

The kurgan is located on the plateau of the left bank of the Velika Vylovka River (the right bank of the Mzha River, the right tributary of the Siversky Donets River), 4 km to the east of the watershed along which the Muravsky Way to the steppe passed. It consists of 53 kurgans. In total, 18 graves under 13 kurgans were excavated. All the graves were represented by rectangular pits oriented along the W-E line. Five kurgans were surrounded by moats, where the remains of feasts were found. On five occasions, head orientation to the NW and SE was fixed. Twice, the skeletons of women were placed with their heads facing east. Stretched on the back was the most common body position. The artefacts of men's burials were represented by bronze arrowheads and iron spearheads. Women's burials were accompanied by jewellery (necklaces, pendants, bronze and iron bracelets).

The necropolis functioned from the end of the 5th to the first half of the 4th c. BCE. The specific features of the burial rite leave no doubt that it belonged to the steppe Scythians, who moved to the forest steppe in search of free pastures. Burial artefacts are standard for the Scythian culture of the end of the 5th–4th c. BCE. The necropolis reflects the process of displacement of impoverished groups of nomads from the steppe in the 5th–4th c. BCE. (*182*, *183*)

Location: 49.671775N, 36.167723 E. Grishkivka village, Zmiiv district, Kharkiv region.

Excavations: 2006.

Excavation authors: Denys Grechko, Institute of Archaeology, National Academy of Sciences of Ukraine.

Storage of anthropological materials: Museum of Archaeology of V.N. Karazin Kharkiv National University.

Description of samples:

Two samples were taken for aDNA analysis, one sample yielded a sufficient amount of DNA for further study.

UKR104. *Grishkivka, kurgan 26, burial 2.* The height of the kurgan was about 1 m. Two simultaneous burials attributed to nomads were found there. The pit of burial 2 had a rectangular shape 2.6 x 1.3 m and a depth of 1.3 m. An iron dart, an iron knife with a bone handle, and 29 bronze arrowheads were found. Chronology according to archaeology: last quarter of the 5th – first half of the 4th c. BCE.

Pisochyn *(Пісочин)*

(Archaeology – V. Borodulin; text – S. Zadnikov)

The kurgan necropolis is located on the right bank of the Luchka River (the right tributary of the Udy river), near the southern outskirts of the Pisochyn settlement, Kharkiv district, Kharkiv region. This family necropolis began to form in the second half of 5th c. BCE and continued to function until the end of 4th c. BCE. In total, 35 kurgans with a height varying from 0.3 to 4 m have been explored. The excavated burials date to the period from the middle of 5th to the end of 4th c. BCE. Among artefacts, weapons and gold jewellery were found. The necropolis belongs to the so-called

“military retinue’s kurgan necropolises”, and accordingly, the buried individuals had high social status. (*184*)

Location: 49.945 N, 36.0975 E. Pisochyn settlement, Kharkiv district, Kharkiv region.

Excavations: 1978–1980.

Excavation authors: Vyacheslav Borodulin, M. F. Sumtsov Kharkiv Historical Museum, Kharkiv.

Storage of anthropological materials: M. F. Sumtsov Kharkiv Historical Museum, Kharkiv.

Description of samples:

Three samples were taken for aDNA analysis.

UKR131. *Pisochyn, kurgan 8.* The height of the kurgan was 6.2 m. Two burials were found in the kurgan. Burial 1 (the inlet burial) was a wooden crypt measuring 3.3 x 3.2 m, oriented along the N-S line. The burial was collective and has been attributed to the nomadic elite. Only teeth and decay of a light-yellow colour remained from the buried individuals. In the middle of the tomb, a woman lay with the head oriented to the south. In her skull area, deciduous teeth were found, as well as the remains of a headdress consisting of gold plates depicting a snake-footed goddess. The earrings were a golden ring with zoomorphic blue glass pendants. There were 15 round gold plaques in the chest area and 7 gold rings on the hands. Also, glass beads, iron tweezers, a moulded jug, black glazed kantharos, and a bronze mirror were found in the burial. The second buried individual (woman) lay parallel to the first, with the head oriented to the south. Teeth and gold earrings were found near the skull, gold plaques depicting a deer were found near the chest, and a gold ring was found near the left hand. The third buried individual (of unknown gender) lay along the northern wall, the head oriented to the east, without burial artefacts. Burial 2 (primary burial) was a double burial, which had been robbed. In a pit of 2.5 x 1.8 m there was a wooden tomb. The skeleton of a teenager (UKR131) was lying on the back along the northern wall, with the head oriented to the east. Foot bones, tibia and partial femurs remained from the second buried individual. Two bronze arrowheads were found. Chronology according to archaeology: third quarter of 4th c. BCE.

UKR132. *Pisochyn, kurgan 18.* The height of the kurgan was 6.7 m. The kurgan contained two burials attributed to the nomadic elite. Burial 1 was a robbed double burial. The burial pit was rectangular in shape. The skeleton of a woman was stretched on the back along the northern wall, the head was oriented to the south. Nearby, a bronze mirror with an iron handle, an iron scoop, black glazed kantharos, a bronze cauldron, bones of sacrificial food, and an iron knife with a bone handle were found. The bones of the second skeleton (UKR132) were scattered in the plundered pit, where 13 gold buttons, around 10 glass beads, a Greek amphora, and two iron darts were found. Burial 2 was in the centre of the kurgan. The burial pit was rectangular in shape and measured 4.2 x 2.2 m. The burial was robbed. Human bones, 5 glass beads, 2 gold and 3 glass amphora-like beads, 3 iron pendants were scattered along the bottom of the chamber. Chronology according to archaeology: mid-third quarter of 4th c. BCE.

UKR133. *Pisochyn, kurgan 6.* The burial has been attributed to the nomadic elite. The height of the preserved part of the kurgan was 4 m. The burial pit was rectangular in shape and measured 3.2 x 2.25 m. The skeleton lay along the western wall, with its head oriented to the south in a straightened position. Near the skull, a golden neckband (hryvnia) and a silver earring were found. There were silver rings on the fingers. In the belt area, there were the remains of a leather belt with bronze plates, a iron shield plates, a quiver with 75 bronze arrowheads. Also, a spearhead and two

darts, a Greek pottery jug, and an iron knife with a bone handle were found. Chronology according to archaeology: mid-second half of 4th c. BCE.

Vesele *(Веселе)*

(Archaeology - B. Shramko; text – S. Zadnikov, I. Shramko)

The group of six kurgans is located on the edge of the watershed plateau, on the right bank of the Murom River (right tributary of the Kharkiv River – Udy River, Siversky Donets basin), between Liptsy village and Vesele village. The burial was founded in the Bronze Age, in pre-Scythian times, and had been used during the Scythian period. Two types of graves were discovered under the kurgans: ground pits with supporting pillars and a ceiling, as well as a catacomb. The burial was explored in 1978 by an expedition of the Kharkiv University under the leadership of B. A. Shramko. (*172–174*, *177*, *180*, *185*)

Location: 50.183969N, 36.496846 E. Vesele village, Kharkiv district, Kharkiv region.

Excavations: 1978.

Excavation authors: Boris Shramko, V.N. Karazin Kharkiv National University, Kharkiv.

Storage of anthropological materials: Museum of Archaeology of V.N. Karazin Kharkiv National University, Kharkiv.

Description of samples:

Two samples were taken for aDNA analysis.

UKR109, UKR110. *Vesele, kurgan 4, burial 1.* The burial was attributed as nomadic. The kurgan was 1.65 m high. The burial chamber was made as a catacomb. The entrance chamber had two steps. The bottom of the grave was at a depth of 5.20 m above the level of the reference point. The burial was robbed. The remains of two skeletons were found in the grave, the bones were randomly scattered in the burial chamber. Two human skulls were found near the centre of the grave. One of them (UKR109) had a lifelong deformity of the skull that gave it an unusually elongated shape. Other body bones (UKR110) were found in different parts of the burial. The preserved things were also scattered. Fragments of an iron awl, an iron hook for hanging a quiver, a bronze quiver, an iron two-hole cheek-piece, an iron ring from a fishhook, two gold hemispherical plaques with black glazed Attic camphor of the 4th century BCE were found. Chronology according to archaeology: 4th c. BCE.

Scythian period. Steppe zone of the Northern Black Sea region (Причорноморський степ)

Scythian tribes appeared in the Northern Black Sea region in the 7th c. BCE. Most known Scythian landmarks in the region (fortifications, settlements, kurgans) are dated to the 2nd half of 5th to the 4th c. BCE. Scythians were engaged in agriculture and cattle breeding, and had close ties with the Greek colonies. A large number of burial sites are concentrated in the Lower Dnieper area, such as Chortomlyk, Solokha, Gaimanova Grave and others, related to the Scythian aristocracy. The Mamay-Gora, one of the largest in the Black Sea steppes, was a burial place mainly for the ordinary population. (*108–112*)

Mamay-Gora *(Мамай-Гора)*

(Archaeology – G. Toshev, S. Andrukh; text – G. Toshev, I. Shramko, S. Zadnikov)

The archaeological site has been described under the Neolithic Azov-Dnieper culture section.

In Scythian time, the Mamay-Gora was the burial ground of the ordinary population. It is one of the largest necropolises in the Black Sea steppes: more than 400 Scythian kurgans and ground burials have been found there. Individuals belonging to Scythian time (adults and children) were buried in catacombs and ground pits. They were stretched out on their backs, with their heads mainly to the west. The accompanying grave goods included household items, jewellery, weapons, and rare items of Greek origin. These artefacts date the Scythian burial of Mamay-Gora to the second half of the 7th to to the first third of the 3rd c. BCE. (*186*, *187*)

Location: 47.432845 N, 34.27259 E. Mamay-Gora tract, Velyka Znamianka village, Vasyliv district, Zaporizhzhia region.

Excavations: 1989, 2002.

Excavation authors: Svitlana Andrukh, Gennady Toshev, Zaporizhzhia National University, Zaporizhzhia.

Storage of anthropological materials: Zaporizhzhia National University, Zaporizhzhia.

Description of samples:

Two samples were taken from Mamay-Gora burials of the Scythian times (*186*, *187*).

UKR013. *Mamay-Gora, kurgan 33, burial 1.* Excavated in 1989. Attributed as nomadic. The burial was found at the depth of 1,2 m. The entrance pit of 2.8 m in length was extended along the W-E line. The roof collapsed. At the depth of 0.15 m there was a step along the long southern wall, 0.4–0.6 m in width. The burial chamber was located parallel to the entrance pit from the north. It was of an oval shape, 2.8x1.1 m. The chamber's bottom was 0.25 m lower than the bottom of the entrance pit. The burial had been robbed in ancient times. Some human bones were found in the filling. A group of bones of the upper part of the body were found at the western end of the chamber. The bones of the legs and the right hand were *in situ*. Judging by their location, the skeleton lay stretched out on its back, with the head oriented to the west. Among the bones, a clay whorl, six grey beads, and a fragment of an iron bracelet were found. Chronology according to archaeology: 4th c. BCE. (*108*)

UKR014. *Mamay-Gora, kurgan 173, burial 1.* Excavated in 2002. Attributed as nomadic. The burial had been robbed. It was in a catacomb of oval shape (2.6x0.7 m), elongated along the west-east line. The entrance was found at a depth of 1.2 m. At a depth of 0.85 m, on the south side, a 0.25 m wide step was traced, descending obliquely to a depth of 1.2 m. The depth of the bottom was 1.7 m. The chamber was oval, its length was 2.8 m, the width in the western part was 1 m and in the eastern part 0.55 m. The filling of the entrance pit and the chamber consisted of dense black soil with a slight admixture of loam. In the western part of the entrance, at a depth of 0.6 m, there were human bones (skull, shoulder blades, ribs) and an animal bone. Among them, a knife handle and fragments of a spear were found. In the bottom part of the chamber, the rest of the human bones lay. In the western corner, bones of a large animal (a young cow) with fragments of iron staples and remains of a wooden tray were found. In the centre of the chamber, closer to the northern wall, one three-bladed and one triangular arrowhead were found. Chronology according to archaeology: 4th c. BCE. (*111*)

Late Scythian period of Crimea (Пізньоскіфський період у Криму)

The Late Scythian culture of Crimea (known as Taurica in the Roman era) is represented by settlements, kurgans and ground burials in the steppe part and foothills of the peninsula. It emerged during the late Hellenistic period (end of the 4th–3rd c. BCE) and persisted until the 3rd c. CE (*188*, *189*). The Late Scythian culture encompassed remnants of various ethnic groups that inhabited Taurica at that time: Scythians, Tauri (descendants of Cimmerians), Thracians, and Sarmatians. The formation of the culture is associated with the gradual sedentarisation of the barbarian population, accompanied by changes in economic activities.

Funerary structures of the Late Scythian culture included earthen and stone tombs (crypts), as well as pit-type graves with a niche. The material assemblage is represented by a wide range of clay vessels (pots, footed bowls, incense burners), antique ceramics (amphorae and tableware), arrowheads, and tools. Anthropological materials have been studied at sites such as Naples, Belyaus, and Zavyetne (*188*).

Maslyny *(Маслини)*

(Archaeology – O. Latysheva, text - V. Kotenko)

The kurgan burial site belonging to Late Scythian culture is located in the steppe zone of the Northwestern coast of the Crimean Peninsula, near the Severne village, in the Chornomorsk district of the Autonomous Republic of Crimea. It is situated about 1 km southeast of the Greek settlement of Maslyny, which dates from the 4th to the mid-2nd century BCE. In the Hellenistic period, the settlement of Maslyny was part of the chora of Tauric Chersonesos (*190*, *191*).

The burial sites in the region are dated to the late 5th to the early 3rd c. BCE. The height of the barbarian kurgans at the Maslyny settlement ranged from 0.5 to 0.7 m. One of them was archaeologically examined in 1976. Artefacts typical of the Late Scythian archaeological culture were found in the burial. The nature of the material obtained (arrowhead, handmade vessel) allows us to speculate that it was not a Greek but a barbarian kurgan, which was located near the Greek settlement. Similar burial sites have been repeatedly investigated in the region and are associated with the presence of Scythians on these lands. In particular, such tombs are typical for kurgans in Western Crimea, dating from the late 5th to the early 3rd c. BCE (*192*). Based on known analogies, the concentration of bones on the flooring may suggest that it was a familial burial ground of sedentary Scythian population. However, with only one kurgan excavated, confirming these conclusions is currently challenging.

Location: 45.685922 N, 33.041920 E; Severne village, Chornomorsk district, AR Crimea, Ukraine.

Excavations: 1976.

Excavation authors: Latysheva V.O., V.N. Karazin Kharkiv National University.

Storage of anthropological materials: Museum of Archaeology of V.N. Karazin Kharkiv National University, Kharkiv.

Description of samples:

Four samples were taken for aDNA analysis, three yielded a sufficient amount of DNA for further study.

UKR051, UKR052, UKR053. *Maslyny, kurgan 1, burial.* The kurgan's height was about 0.7 m. It was surrounded by a stone ring (width 0.2–1.2 m) and had a limestone pavement (2.2x2 m) at the level of the ancient ground. On the pavement, poorly preserved human bones were found, not in anatomical order (burial position and orientation are unknown). Among the bones, 7 skulls were found (including UKR051, UKR052, UKR053), two of which, judging by the teeth, belonged to children. The artefacts were characteristic of barbarian burials (clay bowl, triangular bronze arrowhead, 10 beads). At 2 m to the east from the centre of the kurgan, another burial was discovered under the stone ring (depth 0.5 m from the reference point). This burial exhibited better preservation, skeleton lay in a contracted position on the left side, head oriented to the northwest, facing to the northeast. The left arm was bent at the elbow, the hand near the face, while the right arm was slightly bent and lowered downward. Among the accompanying artefacts, only an iron knife was found near the right femur. Chronology according to archaeology – end of 2nd to 1st c. BCE.

Greeks of Antiquity on the Northern Black Sea coast (Античні греки у Причорномор'ї)

(Text - O. Smyrnov)

During the ancient Great Greek colonization (8th–6th c. BCE), one of the primary directions was the Northern Black Sea region (mid-7th to 5th c. BCE). For a thousand years, the Lower Southern Bug Region represented the most intense point of penetration for antique culture in Southern Ukraine.

In the mid-6th c. BCE, migrants from Miletus founded the city-state of Olbia. It was located on the right bank of the Southern Buh Estuary near the village of Parutyne in the Ochakov district of the Mykolaiv region. From the 6th c. BCE to the 3rd c. CE, Olbia represented a typical Greek polis. It had a functioning temenos and an agora as centres of socio-economic, political, and cultural life. Local currency was minted, a temple was erected, along with trading rows and a gymnasium. Defensive stone walls and towers were built around the city. Olbia had strong trade and cultural ties with the population of the South Black Sea region, as well as with Mediterranean ancient cities like Ionia, Chios, Samos, Rhodes, Corinth. Like other Greek cities, the Olbian state encompassed a substantial agricultural territory known as the chora. The archaeological sites of the chora are represented by over 150 objects (settlements, necropolises) from various periods of antiquity (archaic, classical, Hellenistic, and Roman times).

Olbia's history is divided into two stages. The first stage (mid-6th c. to 49–44 BCE) is characterised by gradual economic and cultural development, which was interrupted by a Getae (Thracian-related tribe) attack (the mid-1st c. BCE). In the subsequent stage (second half of the 1st c. BCE to the middle of the 3rd c. CE), Olbia faced intense pressure from local tribes and eventually fell into political dependence on Rome. In the mid-3rd c. CE, presumably due to the Gothic invasion, the city ceased to exist as an ancient centre.

Oleksandrivskyi necropolis *(Олександрівський некрополь)*

(Archaeology, text – O. Smyrnov)

The Oleksandrivskyi necropolis dates to the transition between the 4th and 3rd c. BCE. It is located within the territory of the Admiralteysky Park in the Mykolaiv city centre (the intersection of Naberezhna Street and 2nd Slobidska Street). This necropolis was situated near the ancient settlement known as "Zavod im. 61-Komunara", which dates to the times from the 4th c. BCE to the 1st c. CE.

The necropolis was discovered in 2010, but its exact dimensions and boundaries have not been established due to urban development. A total of 25 burials were discovered at this site, arranged in a cluster-like pattern.

During the Hellenistic period, from the 4th to the 3rd c. BCE, the territory of present-day Mykolaiv was part of the Olbia state (Olbia chora). This period was characterised by the expansion of the agricultural region of Olbia, which served as a vital economic base for the city-state following the invasion of the Macedonian army. (*193–199*)

Location: 46.9724 N, 32.00901 E. Naberezhna Street, Mykolaiv city.

Excavations: 2011.

Excavation authors: Olexandr Smyrnov, Petro Mohyla Black Sea National University, Mykolaiv, Ukraine.

Storage of anthropological materials: Laboratory of Archeology and Ethnology, Petro Mohyla Black Sea National University.

Description of samples:

Three samples were taken for aDNA analysis and two yielded a sufficient amount of DNA for further study.

UKR152. *Oleksandrivskyi necropolis, burial 5.* Burial with a grave niche. Partially disturbed by modern construction activity. The entrance pit and burial chamber were not traceable. The stone structure at the entrance to the burial chamber was composed of limestone slabs, with one of the slabs serving as a limestone altar of the 'Olbia type'. To the north, there was a skeleton extended on its back, with the head oriented to the northeast. The right arm was straight, and the left arm was on the pelvis. Only the femurs remain from the legs. Chronology according to archaeology: 4th–3rd c. BCE. Chronology according to ^{14}C dating: 392–206 cal BCE (2253±29 BP).

UKR153. *Oleksandrivskyi necropolis, burial 6.* Burial with a grave niche. Partially disturbed by modern construction activity. The entrance pit measured 1.28 m in length and 0.26 m in width and was oriented along the W-E line. The grave-pit was separated from the burial chamber by a double-row stone masonry. The chamber was rectangular, measuring 2.32 m in length and 0.69 m in width, with an orientation along the W-E line. The skeleton lay in an extended position on the back, with the head oriented to the west, arms positioned near the pelvis, and straight legs. Near the left shin, bones of sacrificial food (shallow horned cattle) were found. Twelve arrowheads were discovered near the left femur (possibly the remains of a quiver), additionally, 9 arrowheads were found in other places of the burial. All 21 arrowheads were made of bronze and belonged to three types: 8 three-bladed with a prominent bushing, 6 three-bladed with a less prominent bushing, and 7

triangular ones with a concealed bushing. According to archaeological artefacts, the burial dates to the 4th–3rd c. BCE. The chronology according to ^{14}C dating: 746–401 cal BCE (2415±30 BP).

IRON AGE

Sarmatian Period

Sarmatians were a confederation of Iranian-speaking nomadic tribes that originated in the central Eurasian Steppe and were closely related to Scythians. They dominated the North Pontic steppe and forest-steppe from the 3rd c. BCE to the 4th c. CE. Sarmatian nomadic society, most likely, was a temporary military-political association with such attributes of statehood as a common territory, supreme power, a certain social stratification of society, possibly a professional army. Sarmatians had high military potential and imposed tribute and indemnities, controlled over trade routes, used mediation and protectionism in trade, and finally, robbery during raids.

The main occupation of the Sarmatian tribes on the territory of Ukraine was nomadic cattle breeding. They bred mainly horses and sheep. The fighting horse was an important part of the military culture of the Sarmatians. Early Sarmatian burials are scattered across the steppe without any system, most of them are the graves of warriors. This indicates that the initial advance of the Sarmatians into the North Pontic steppes was carried out by military detachments. The lack of settlements, the finds of weapons and horse equipment in ground burials indicate a nomadic lifestyle.

The Sarmatians had contact with other populations of the region: with the inhabitants of ancient cities, the Hellenes; Late Scythian tribes of the Lower Dnipro and Crimea; carriers of the Zarubinets culture and the population of the Chernyakhiv culture. The spread of Sarmatian-type items — weapons, horse equipment, some forms of clothing, and jewellery — in the material culture of the ancient centres of the first centuries BCE got the name Sarmatization of the ancient world.

In the 3th–5th c. BCE Sarmatians were conquered by Goths and Huns, later they were assimilated by Slavs.

Forest-steppe zone of Dnipro-Donets. Siversky Donets group of landmarks *(Дніпро-Донецький лісостеп, Сіверсько-Донецька група пам'яток))*

The Siversky Donets group of landmarks has been described under the Scythian period section.

Liubivka *(Любівка)*

(Text – I. Shramko, S. Zadnikov)

The multi-layered settlement of Liubivka is located on the first floodplain terrace of the right bank of the Merla River (a right tributary of the Voronezh River within the Dnipro River basin). The surface of the settlement is subject to annual erosion, and there are visible ash spots on the surface. Artefacts from the Neolithic, Bronze Age and Scythian times, Saltiv, Bondarikha and Chernyakhiv cultures, early Slavs period were found in Liubivka and its surroundings. In 1974, three excavations were conducted at the settlement, revealing a cultural layer from the Scythian period (the latter half of the 6th c. BCE). Additionally, isolated fragments of pottery from the Bronze Age

were discovered, representing various cultures such as Catacomb, Zrubna (Srubnaya), and Bondarikhinska. Furthermore, a unique Sarmatian girl's burial was excavated. (*200*, *201*)

Location: 50.007291 N, 35.038043 E. Liubivka village, Bohodukhiv district, Kharkiv region.

Excavations: 1974.

Excavation authors: Borys Shramko, V.N. Karazin Kharkiv National University.

Storage of anthropological materials: Museum of Archaeology of V.N. Karazin Kharkiv National University.

Description of samples:

One sample was taken for aDNA analysis.

UKR160. *Liubivka, Excavation 3, burial 1.* The burial was embedded in the cultural layer of a Scythian-period settlement and was defined as Sarmatian based on the artefacts found. The bottom of the trapezoid-shaped burial pit was in yellow sand at a depth of 1.35 m. The skeleton of a woman was found lying on her back in an extended position, with her head oriented to the north-northeast. On her right clavicle, there was a bronze fibula (brooch) with an iron pin. On the left side of her chest, there was another bronze fibula (brooch) with a suspension loop. Bronze bracelets were on both her arms. Around the neck, 43 glass, coral and amber beads were found. Chronology according to archaeology: 1st – 3rd c. CE.

Chernyakhiv Culture (Chernyakhiv-Sântana-de-Mureș Culture) *(Черняхівська культура)*

(Text – B. Magomedov, S. Didenko, O. Petrauskas, R. Reida, A. Heiko, S. Sapiehin)

The Chernyakhiv culture (Chernyakhiv-Sântana de Mureș culture) was created by barbarian tribes in late Roman times in Southeastern Europe. It was widespread in most of the forest-steppe and steppe regions of Ukraine, Moldova, parts of Romania, and some neighbouring areas of the Russian Federation from the beginning of the 3rd to the middle of the 5th c. CE (*28*).

Most researchers consider the composition of this culture to be polyethnic. It included Eastern Germans, early Slavs, Getae-Dacians, Sarmatians, as well as late Scythians and Alans (*28*). It is also likely that some portion of the provincial Roman population and immigrants from ancient centres of the Northern Black Sea region were included in it (*27*, *202–205*). Now this is confirmed not only by archaeology but also by anthropological data (*206*, *207*). According to most researchers, the leading role in this tribal alliance was played by East Germanic tribes Goths and Gepids (*28*, *208*, *209*).

The analysis of archaeological materials from Chernyakhiv sites shows that there are strong traditions of the Wielbark culture and other Germanic cultures of Central Europe in the types of structures, burial rites, moulded ceramics, clothing details, ornaments, and everyday items. The style of pottery developed as a result of the synthesis of the provincial Roman craft tradition and the Wielbark tradition of clay pottery. Thanks to the strong influence of late antique centres, the material culture of Chernyakhiv-Sântana de Mureș reached a high level.

The dominant ethnic group within the Chernyakhiv population were the carriers of the Wielbark tradition – the Goths. They left behind "classic" Chernyakhiv artefacts, which can be conditionally referred to as "Kosanov type" landmarks.

Two local groups of antiquities were related to other ethnic groups. In the Upper Dniester region, there was a western group of Slavs, represented by the "Cherepin type" landmarks. In the Black Sea steppe, there were late Scythians and Sarmatians-Alans who formed mixed communities with the Germanic people. This population left behind "Black Sea type" landmarks. Some Chernyakhiv sites in Eastern Ukraine have elements of the neighbouring Kiev culture (eastern group of Slavs) whereas elements of the Thracian-Carpi culture are found in Moldova and Muntenia, but they do not form independent types.

After the arrival of the Huns, the Germanic and Scytho-Sarmatian population left the Chernyakhiv culture area. Slavic tribes began to dominate in the forest-steppe zone, eventually giving rise to the Prague and Penkivska cultures.

Chernyakhiv culture in the Dnipro left bank forest-steppe *(Черняхівська культура лівобережного лісостепу)*

The Dnipro left bank forest-steppe in late Roman times was part of the Chernyakhiv archaeological culture area. Hundreds of sites are known in this region now (*210*, *211*).

It is evident that the ethnic components within the Chernyakhiv culture had varying proportions and significance in different regions and during different periods of the culture. For the Dnipro left bank forest-steppe, the Eastern Germanic component is known (as seen in the Kompanitsi burial) (*212*) but relatively limited. Instead, an important ethnic component, at least from the middle of the 4th c. CE, consists of migrants from a nomadic (adjacent to the Chernyakhiv culture area) environment and possibly a late Scytho-Sarmatian ethnic component (*205*, *213*). The contact zone between nomadic and settled tribes at that time passed through the Vorskla River basin. The nomadic tribes were represented by the Alans. The result of these contacts was the active integration of this ethnic component into the Chernyakhiv community, which can be traced through specific archaeological sites from southeast to northwest of the Dnipro left bank forest-steppe. To observe an example of this process, one can examine the cemeteries of Storozhove (*214*), Kantemirivka (*215*), and Shyshaky (*213*).

The Chernyakhiv culture of the Dnipro left bank forest-steppe retained its character even after the arrival of the Huns in the region and, in some cases, continued to develop. Notably, the Shyshaky and Voitenky cemeteries serve as vivid examples of this. It is likely that the relationships with newcomers among the bearers of the Chernyakhiv culture in the region did not have dramatic consequences, and contacts with ancient centres of the Northern Black Sea and Roman provinces persisted (although the nature of these contacts — whether trade or military interactions — remains unclear).

Shyshaky *(Шишаки)*

(Archaeology, text - A. Heiko, R. Reida, S. Sapiehin*)*

The Shyshaky cemetery is one of the largest necropolises of the Chernyakhiv culture in the Dnipro left bank forest-steppe as well as in the entire Chernyakhiv culture area. It is located near the urban-type settlement of Shyshaky (Myrhorod district, Poltava region). Archaeological excavations at the site began in 2009 and continued until 2017. From the beginning of archaeological work, 156 burials were excavated.

The cemetery is characterised by both inhumations and cremations. The predominant group consists of inhumation burials with the heads oriented to the west. The next in terms of quantity are inhumation burials oriented to the north. There are individual inhumation burials with eastern and southern orientations. Burial-cremations constitute the smallest group. The inhumation burials were divided into four types: 1) rectangular pit; 2) ledge-pit; 3) catacomb; 4) tomb (*213*). Some burials exhibit signs of nomadic burial traditions, which, in our opinion, belong to the Alanic component. Among these signs are ledge-pits and catacombs, rituals involving fire, bird eggs, and bones. Elements that could be clearly attributed to other ethnic components, including Eastern Germanic, were not found at the Shyshaky cemetery.

Among other research of the site, it is worth mentioning the DNA analysis conducted on the content of the clay vessel from burial 124 (*216*).

All the examined burials at the Shyshaky cemetery, where chronological indicators were found, were dated within the period from the mid-4th to the first half of the 5th c. CE (*202*, *217*). These burials primarily include complexes with glassware of provincial Roman production (*202*, *218*).

Location: 49.856205 N, 33.981227 E. Shyshaky village, Shyshaky district, Poltava region.

Excavations: 2009–2017.

Excavation authors: Anatoliy Heiko (2009-2011), "Center for Protection and Research of Archaeological Monuments" of the Poltava Regional Council; Roman Reida (2012-2017), Institute of Archaeology, National Academy of Sciences of Ukraine, Kyiv, Ukraine.

Storage of anthropological materials: "Center for Protection and Research of Archaeological Monuments" of the Poltava Regional Council, Ukraine.

Description of samples:

Ten samples were taken from Shyshaky burial, seven of them yielded a sufficient amount of DNA for further study.

UKR121. *Shyshaky, burial 88.* Excavated in 2014. Burial of an adult woman. The skeleton was lying flat on its back, the head was oriented to the north. The upper part of the postcranial skeleton was broken. The upper limbs were stretched along the body, the bones of the lower limbs were slightly displaced. The burial had numerous accompanying artefacts: 11 vessels were found in the grave (a large vase-bowl, two-handled jug, decorated pottery cup, bowl-dish, pot, vase-bowl, a small single-handled jug of antique production, a medium-sized bowl, a small bowl, and two pots). Additionally, there were two silver fibulae, a necklace made of carnelian, glass, coral, and amber beads, a small silver belt buckle, a horn comb, a large spindle, a bone awl, and a miniature bronze knife. Next to the pelvic bones, there were bones of large horned cattle, presumably representing accompanying food. Based on the chronological indicators, the burial is dated to the second half of the 4th – the beginning of the 5th c. CE.

UKR122. *Shyshaky, burial 89.* Excavated in 2014. Burial of an adult individual. The skeleton was extended on the back, the head oriented to the west. The bones of the upper limbs were on top of the pelvic bones, and the lower limbs were extended. The cranial and postcranial skeleton was relatively well-preserved. No artefacts were found. Chronology according to archaeology: 4th c. CE.

UKR123. *Shyshaky, burial 94.* Excavated in 2014. Burial of an adult individual. The skeleton was extended on the back, the head oriented to the west with a slight deviation to the south. No torso

bones were found in the burial, the bones of the upper limbs were positioned parallel to each other, while the legs were closely brought together. There were no accompanying artefacts. Chronology according to archaeology: 4th c. CE.

UKR125. *Shyshaky, burial 97.* Excavated in 2014. Burial of an adult individual. At a depth of 1.96 m, a cluster of anthropological remains was found, consisting of two femurs, a pelvic bone, several ribs, and vertebrae, chaotically arranged in the western sector of the pit. Subsequent research revealed another part of the burial that remained undisturbed. The skeleton was placed on the back in an extended position, the head was oriented to the west with a slight deviation to the south. The skull, pelvic bone, and possibly the lower jaw, had been displaced at some point after the burial. Traces of wood were found – remnants of the burial structures. No accompanying artefacts were found. Chronology according to archaeology: 4th c. CE.

UKR126. *Shyshaky, burial 103.* Excavated in 2014. Burial of an adult individual. The skeleton was extended on the back, the head oriented to the west. Near the right shoulder, a small pot was found, surrounded by animal bones, which were presumably remnants of accompanying food. Chronology according to archaeology: 4th c. CE. Chronology according to ^{14}C dating: 131–325 cal CE (1820±26 BP).

UKR128. *Shyshaky, burial 114.* Excavated in 2014. The skeleton was of satisfactory preservation, laying on the back in an extended position, the head was oriented to the southwest. Some small bones from the upper and lower limbs were missing, likely due to the activities of burrowing animals. No accompanying artefacts were found. Chronology according to archaeology: 4th c. CE. Chronology according ^{14}C dating: 229–361 cal CE (1771±26BP).

UKR129. *Shyshaky, burial 115.* Excavated in 2014. Burial of an adult individual. The skeleton was extended on the back, the head oriented to the west with a slight deviation to the south. The skeleton was severely damaged: only fragments of large bones from the upper and lower limbs were found, and the cranial skeleton was heavily damaged. The burial stands out for its relatively rich artefacts, including two belt buckles with fragments of leather belts (one silver, the other made of a copper alloy), remnants of heavily damaged fragments of a horn comb with copper alloy rivets, a pottery bowl with a conical glass cup inside, adorned with drops of blue and dark red colour; another three pottery bowls and a black-glazed jug of the left-bank type. Based on the chronological indicators found in the burial, the complex is dated to the late 4th to early 5th c. CE. (*219*). Chronology according ^{14}C dating: 245–401 cal CE (1741±27BP).

Komariv *(Комарів)*

(Archaeology, text – O. Petrauskas)

The Komariv settlement is located in the middle reaches of the Dniester River – this river was a boundary between the eastern and western parts of the Chernyakhiv-Sântana de Mureș culture. The settlement existed from the beginning of the 3rd to the middle of the 5th c. CE and was a centre oriented towards mass production of glass items such as cups, beads, gaming pieces, etc (*220*). It was the only glass workshop outside the Roman Empire within the Barbaricum territory in Europe. It is highly likely that Komariv was not only influenced by provincial Roman technologies but also had a significant population of Romanized individuals (*70*, *221*).

The ethnic and cultural composition of the population in Komariv was heterogeneous and included various ethnic groups and social strata, originating from the late antique, Scytho-Sarmatian, early

Slavic, Dacian, and East Germanic backgrounds. Dominant ethnic groups included the late Scythians, Sarmatians, and early Slavs, while to a lesser extent, there were Getae-Dacians and people from Roman provinces. The East Germanic component was very restricted in this context (*70*).

Location: 48.535183 N, 26.9676 E. Komariv village, Dnistrovskyi district, Chernivtsi region.

Excavations: 2013.

Excavation authors: Oleg Petrauskas, Institute of Archaeology, National Academy of Sciences of Ukraine, Kyiv, Ukraine.

Storage of anthropological materials: Archaeological research Laboratory, National Pedagogical Dragomanov University, Kyiv, Ukraine.

Description of samples:

One sample was taken for aDNA analysis.

UKR049. *Komariv, burial 2.* According to archaeological data, burial 2 exhibited traits typical for late Scytho-Sarmatian population (ledge-pit) and belonged to a woman under the age of 20. The grave depth was 2.67 m. Remains of wooden roofing were preserved. The head of the skeleton was oriented to the north. The skeleton was ritually destroyed in ancient times. In the burial, the following items were found: 15 glass beads of prismatic shape; a silver fibula clasp; two spinning wheels; six pottery vessels. In addition, the burial was accompanied by a sacrificial animal (a rooster). The artefacts and the position of the burial in the overall plan of the cemetery allow dating it to the second half of the 4th to the beginning of the 5th c. CE. Chronology according ^{14}C dating: 169–338 cal CE (1805±26 BP).

Lehedzyne *(Легедзине)*

(Archaeology, text - B. Magomedov, S. Didenko)

The cemetery of Chernyakhiv culture near the village of Lehedzyne was excavated by B. V. Magomedov and S. V. Didenko in 2008–2009. Approximately half of the necropolis (690 sq. m) was excavated. A total of 81 burials were studied, including 47 cremations and 34 inhumations. The cremations included both with urn and without urn type. Among the inhumations, 2 were conducted in pits with niches, and 3 in ledge pits. The findings were mostly characteristic of the Chernyakhiv culture archaeological artefacts. Wheel-made pottery predominated among ceramics. Moulded vessels had analogies in the Wielbark culture. Imported pottery included fragments of amphorae, red-lacquered items, and glass cups. The site yielded clothing and items of everyday use, such as fibulae and buckles, various beads, pendant amulets, horny and iron combs.

The Lehedzyne burial site dates to the late 3rd – the end of the 4th c. CE. The locals were primarily Gothic people, who constituted the majority of the Chernyakhiv culture population. Burials in pits with a niche and with a ledge may suggest the presence of people of Sarmatian origin within the community. (*28*, *222*, *223*)

Location: 48.806513 N, 30.508557 E. Lehedzyne village, Talniv district, Cherkasy region.

Excavations: 2008-2019.

Excavation authors: Borys Magomedov, Institute of Archaeology, National Academy of Sciences of Ukraine, Kyiv; Serghii Didenko, National Museum of History of Ukraine, Kyiv.

Storage of anthropological materials: Institute of Archaeology, National Academy of Sciences of Ukraine.

Description of samples:

Three samples were taken for aDNA analysis, two yielded a sufficient amount of DNA for further study.

UKR045. *Lehedzyne, burial 16.* Excavated in 2009. Inhumation of a woman aged 25–35 years. The burial pit had a niche, oriented to the north. The rectangular entrance measured 1.7 x 0.65 m. The burial chamber was irregularly oval-shaped, measuring 1.8 x 0.6–1.1 m. The depth of the entrance pit was 1.32–1.40 m, and the depth of the burial chamber was 1.36–1.55 m. The skeleton was lying on the back in an extended position. On the chest, there were 18 carnelian beads and two mother-of-pearl beads. Around the neck, there was a leather cord with six silver and three leather pendants. Fragments of an iron fibula were found above the left clavicle. Chronology according to archaeology: the first to third quarters of the 4th c. CE.

UKR047. *Lehedzyne, burial 24.* Excavated in 2009. Inhumation, oriented to the north, partially destroyed. The burial of a woman aged 20–25 years. The pit's depth was 1 m, and the pit's contours were not traceable. The skeleton was lying in an extended position, with legs shifted to the east and arms outstretched. Cervical vertebrae and part of the chest were missing, the pelvis was damaged. The ulnas were fractured, and the lower part of the arms was missing. The skull was in place, but the mandible was found in the fill, near the feet. To the west of the skull, there were vessels: a bowl, under which fragments of a one-handed vase, under which fragments of a pot. A fibula lay on the left scapula of the skeleton. Near the left foot, there were a red-clay bowl, a cup, an amphora, and a bead. There were traces of a bronze object (possibly a buckle) on one of the lower vertebrae. Chronology according to archaeology: the second half of the 4th c. CE.

Zolochiv *(Золочів)*

(Archaeology - I. Shramko, text – I. Shramko, S. Zadnikov)

The burial was located on the western outskirts of Zolochiv town, on the edge of the right bank of the Udy River (Siverskyi Donets basin). It was accidentally discovered by local residents near a quarry while extracting sand for household purposes in October 2008. The object has been completely destroyed, but it was possible to ascertain some details of the burial and collect most of the items left in the grave during the burial ceremony. Fragments of pottery vessels were collected, as well as artefacts made of bone, glass, and metal. Only traces of part of the burial chamber were visible. (*224*, *225*)

Location: 50.279939 N, 35.957792 E. Chalogo street, Zolochiv city, Bohodukhiv, Kharkiv region.

Excavations: 2008.

Excavation authors: Iryna Shramko, Museum of Archaeology of V.N. Karazin Kharkiv National University.

Storage of anthropological materials: Museum of Archaeology of V.N. Karazin Kharkiv National University.

Description of samples:

One sample was taken for aDNA analysis.

UKR102. *Zolochiv, ground burial 1.* The burial chamber was in the form of a rectangular pit with a niche. The burial chamber was excavated in sandy soil, measuring 2.10 m in length, and was oriented along an east-west axis, with its bottom situated at a depth of 2.70 m. There was a noticeable trace of a vertical shaft (dromos) filled with mixed soil. The human skeleton was oriented with the head to the west. The accompanying artefacts were located on the skeleton's right side. A significant portion of the pots and glassware were broken into fragments, but some items remained intact: a large bi-conical-shaped bowl, a small dish, an iron sword, a dart point, a fragment of an iron knife, a bone comb, a product made of tubular bird bone, and a bronze dart. Human bones had been hidden by modern-day grave robbers in a pit near the burial. Chronology according to archaeology: 4th c. CE.

EARLY MIDDLE AGE

Saltiv (Saltiv-Majaki) culture (Салтівська культура)

(Archaeology – V. Borodulin, V. Aksonov, text – V. Aksenov)

The Saltiv culture got its name from an archaeological site near the village of Verkhnii Saltiv, which is represented by a hillfort, a burial ground, and a series of settlements. The site was found in 1900 by the local educator V.O. Babenko. The excavated artefacts allowed to identify a distinct archaeological Saltiv culture associated with the Khazar Khaganate. The culture dates to the final quarter of the 1st millennium CE (the second half of the 8th to the first half of the 10th c. CE) (*226*).

The sites of Saltiv culture have been found across an extensive area, ranging from the Caucasus Mountains in the south to the Middle Volga region in the north, and from the Volga River in the east to the lowlands of the Dnieper River in the west, primarily encompassing steppe regions. Within this territory, researchers identify local types, including the forest-steppe, the mid-Don steppe, the lower Don steppe, the Azov Sea region, the area of the Caucasus foothills and plains, and the Crimean region (*227*, *228*).

Within this area, diverse types of archaeological sites have been found, including hillforts, settlements, temporary camps, catacombs, pit burials, and kurgans. Hillforts have been found with stone walls as well as wooden and ground fortifications (*74*, *229–232*). At settlements, excavations have revealed dugouts, semi-dugouts (*227*, *233–235*), yurt-like dwellings (*236*), household buildings and pits (*231*, *237–239*), as well as religious complexes (*240*).

Various burial practices have been identified, including burials in catacombs (*230*, *241–243*), inhumation pits of various types (*244–248*), cremation burials with subsequent placement of human remains and artefacts in pits or urns (*249*, *250*), and inhumation burials in kurgans (*228*). The diversity of burial customs within the Saltiv culture indicates heterogeneity of population. The following burial practices are associated with specific ethnic groups: inhumations in underground catacombs with Alans, inhumations in pits with Bulgars and mixed Turkic-Ugric population, inhumations in kurgans with Khazars, cremations with Turkic, Slavic, and Adyghe-Abkhaz individuals.

The spread of the Saltiv culture within the forest-steppe and steppe zones led to a diverse range of economic activities, including various forms of agriculture and cattle breeding (*227*, *228*, *233*, *251*).

The presence of weaponry items in the materials from burials, such as sabers, armor-piercing spearheads, battle-axes, bone and horn arrowheads, iron arrowheads, as well as equestrian gear items (bits, stirrups, horse harness decorations), suggests that the Saltiv military predominantly consisted of light cavalry units (*252–254*).

The broad cultural and trade exchanges between the Saltiv culture, Byzantium and the Arab Caliphate are evidenced by the elements of lotus-like belt ornaments (*255*), ornaments crafted in a Crimean-Byzantine style (*256*), the presence of early medieval amphorae and pottery produced in Crimea (*257*, *258*), Arab silver dirhams and Byzantine gold soliduses (*259*).

Verkhnii Saltiv *(Верхній Салтів)*

The Verkhnii Saltiv catacomb burial ground was found in 1900 by V.O. Babenko. It is located northward of the Verkhnii Saltiv Hillfort, on the slopes of numerous ravines crossing the high right bank of the Siversky Donez River. From 1900 to 1917, V.O. Babenko excavated three separate sections with catacombs, which he designated as Verkhnii Saltiv I (main), Verkhnii Saltiv II, and Verkhnii Saltiv III burial grounds. An additional burial ground – Verkhnii Saltiv IV – was found in 1989 by the expedition of the Kharkiv Historical Museum led by V.G. Borodulin.

The Verkhnii Saltiv catacomb burial ground I (main) occupies the western and northwestern slopes of the Kapinosova Ravine. Up to 1960, about 417 catacomb burials were excavated there (*250*). It is considered that this section of the burial ground was used throughout the whole culture period, spanning from the second half of the 8th to the middle of the 10th c. CE (*260*). In 1984, excavations at the burial ground were initiated by an expedition from the Kharkiv Historical Museum under the leadership of V.G. Borodulin. From 1984 to 1989, the expedition studied 75 catacombs, one burial in a dromos, 16 burials in pits of various types, and 4 burials of horses in separate pits. These researched burial complexes are dated to the middle of the 8th–9th c. CE (*261*, *262*).

The Verkhnii Saltiv catacomb burial ground III is located on the eastern slope of the right bank of the Siversky Donez River northward of the settlement, beyond the ravine. The cemetery was found in 1902 by V.O. Babenko, who excavated 4 catacomb burials in 1902–1903. In 1959–1961, excavation was carried out by the Institute of Archaeology (Kyiv) led by D.T. Berezovets. During this expedition, 17 catacomb burials and 3 separate burials with horse remains were studied. The Kharkiv Historical Museum expedition under the leadership of V.G. Borodulin, conducted research in 1988–1992. The work resulted in the excavation of 25 catacombs, 5 pit burials, 4 horse burials in separate pits, and 1 burial in a dromos (*250*). Based on coins and elements of a belt set, the burial ground is believed to have originated in the second half of the 8th c. CE and was in use throughout the 9th c. CE (*260*, *261*).

The Verkhnii Saltiv catacomb burial ground IV is located on the eastern slope of the Netechinsky ravine and forms a single area with the Verkhnii Saltiv II burial ground (*263*). In 1989–1990 17 catacombs were explored by an expedition of the Kharkiv Historical Museum led by V.G. Borodulin. An additional 10 catacombs were excavated in 1996 by an expedition of G.S. Skovoroda Kharkiv State Pedagogical University led by V.V. Koloda. From 1998 to 2021, the burial ground was explored by the expedition of the Kharkiv Historical Museum led by V.S.

Aksenov. To date, the total number of studied burial complexes is 164 plus 1 burial of a horse. Most of the studied complexes are dated to the 9th c. CE.

Location: 50.135123 N, 36.799162 E. Verkhnii Saltiv village, Chuhuev district, Kharkiv region, Ukraine.

Excavations: 1986, 1989, 2014.

Excavation authors: Vyacheslav Borodulin (1986, 1989), Viktor Aksonov (2014), M. F. Sumtsov Kharkiv Historical Museum, Kharkiv, Ukraine.

Storage of anthropological materials: M. F. Sumtsov Kharkiv Historical Museum, Kharkiv, Ukraine

Description of samples:

One sample was taken from burial ground I, one from burial ground III and four from burial ground IV. Individuals were referred to as Alans based on the catacomb type of burials.

UKR134. *Verkhnii Saltiv catacomb burial ground I, catacomb 42.* Excavated in 1986. T-shaped catacomb with a floor size of 2.25 x 1.45 m. On the floor, the remains of three individuals were found: two women and a child. The skeletal remains showed deliberate damage, which had occurred in ancient times during a ritual neutralization of the deceased people. One female burial (UKR134) was accompanied by a bronze trapezoid-shaped pendant, a bronze wire bracelet, and a bronze ring. The other female burial contained bronze wire decorations, bronze bells, plate-shaped trapezoid pendants, a glass necklace, and silver earrings. The child's remains were accompanied by glass beads. Chronology according to archaeology: second half of 8th–9th c. CE.

UKR135. *Verkhnii Saltiv catacomb burial ground III, catacomb 11.* Excavated in 1989. The chamber was oriented longitudinally in relation to the dromos, had a size of 2.4 x 1.6 m. The remains of two individuals, a man and a woman, were found on the floor. The skeletons exhibited deliberate damage, which had occurred in ancient times during a ritual neutralization of deceased people. The man (UKR135) was accompanied by the following artefacts: iron battle-hammer, an iron battle-axe, silver decorations from footwear straps, a bronze belt buckle, an iron knife, and a bronze pendant-seal. The female burial was accompanied by bronze earrings, a glass necklace, a bronze mirror, a bronze medallion, 3 bronze wire bracelets, 2 rings, a bronze toiletry case, a bronze belt buckle and a tip, silver decorations from shoe straps. Chronology according to archaeology: second half of 8th–9th c. CE.

UKR136. *Verkhnii Saltiv catacomb burial ground, catacomb 120.* Excavated in 2014. T-shaped catacomb with a floor size of 2.3 x 1.83 m. On the floor, the remains of three individuals (two men and one woman) were found. In the wall of the dromos, a niche had been made, where the remains of a child aged 1–2 years were found, along with a bronze wire bracelet, a bronze bell, and a bronze stamped button. The burial of one man (UKR136) was accompanied by a bronze ring and bronze elements of a belt. The artefacts associated with the other man consisted of bronze elements of a belt and metal elements from shoe straps. On the woman's remains, bronze earrings, a bronze ring, and a glass necklace were found. Chronology according to archaeology: second half of 8th–9th c. CE.

UKR137. *Verkhnii Saltiv catacomb burial ground, catacomb 122.* Excavated in 2014. T-shaped catacomb with a floor size of 1.8 x 0.9 m. On the floor, the remains of two individuals (a man and a child) were found. The man's (UKR137) burial was accompanied by three iron knives, three cast

bronze bells, an iron fibula, iron belt buckle, iron tweezers, and one glass bead. The child's burial was accompanied by a hand-formed clay pot. Chronology according to archaeology: second half of 8th–9th c. CE.

UKR138. *Verkhnii Saltiv catacomb burial ground, catacomb 124.* Excavated in 2014. The catacomb was incomplete and consisted only of a dromos, where the remains of an adult man were found, accompanied by artefacts: a bronze belt buckle, a bronze belt tip, an iron knife, an iron adze-hoe, and a reliquary made of animal bone. Chronology according to archaeology: second half of the 8th–9th c. CE.

UKR139. *Verkhnii Saltiv catacomb burial ground, catacomb 125.* Excavated in 2014. T-shaped catacomb with a floor size of 1.82 x 1.42 m. On the floor, the remains of an adult woman were found, accompanied by bronze butterfly-shaped decorations, two bronze chumbar blocks, two iron knives, a bronze duck-shaped pendant-amulet, a glass bead necklace, bronze wire decorations, and a bronze bell. Chronology according to archaeology: second half of 8th–9th c. CE. Chronology according to ^{14}C dating: 671–874 cal CE (1256±27BP).

Bochkove *(Бочкове)*

(Text, archaeology – Oleksiy Laptev)

The burial ground was found 1.3 km west of the modern western outskirts of the village of Bochkove. The burial ground occupies a kurgan-like elevation on the right bank of the Vovcha River, located 23 km from its confluence with the Siversky Donez River. (*264*, *265*)

Location: 50.314768 N, 37.11063 E. Bochkove village, Chuhuev district, Kharkiv region.

Excavations: 2014.

Excavation author: Oleksii Laptev, M. F. Sumtsov Kharkiv Historical Museum, Kharkiv, Ukraine.

Storage of anthropological materials: M. F. Sumtsov Kharkiv Historical Museum, Kharkiv, Ukraine.

Description of samples:

Eight samples were taken, DNA was successfully extracted from four and three of them yielded a sufficient amount of DNA for further study. Individuals were referred to as Bulgars based on the pit type of burials.

UKR143. *Bochkove, burial 3.* The rectangular-shaped burial pit was oriented along the east-west axis. The filling of the pit was stratified: in its upper part, there was marl, and in the lower part, humus. The burial pit had a circular widening: at a distance of 0.6–0.7 m from the surface, it expanded in all directions. The buried adult woman lay in a wooden coffin, curled up on the right side with her head to the west. The bone preservation was poor and the skull was heavily decomposed. The neck was unnaturally curved: the first cervical vertebrae were oriented along the 'west-east' axis and those adjacent to the thoracic region were oriented along the 'north-south' axis. It is possible to speculate that such a fracture occurred as a result of arranging the required ritual position after death. The burial contained modest but interesting items: two earrings under the skull, a quarter of a dirhem in front of the jaw, a second quarter of a dirhem (which could be combined with the first to form half a coin) in the abdominal area under the left forearm. In the feet area, in the northwest corner, there was a mug.

UKR144. *Bochkove, burial 4.* An inhumation in a ledge-pit with a niche. The filling of the burial pit consisted of marl in the upper part and humus in the lower part (excavated in compact marl). The grave-pit had dimensions of 2.21×0.84 m, the depth was 1.65–1.8 m. The remains of a woman (height 1.45 m) lay stretched out on the back with the head to the north. The right arm was extended along the body with the palm facing down. The left arm was bent at the elbow, with the hand resting on the chest. The bone preservation was poor. Behind the head, there was a bowl with the handle broken in ancient times, and a thin metal mirror. A quarter of an Arabic dirhem coin was found in the mouth, whereas a one-eighth part of the coin was found on the lumbar vertebra. Two cast buttons were found on the collarbones, and a ring with a glass insert was found on a right hand finger. Chronology according to archaeology: 9th c. CE. Chronology according ^{14}C dating: 671–874 cal CE (1256±27BP).

UKR147. *Bochkove, burial 6.* An inhumation in a ledge-pit with a niche. The bottom of the burial pit was found at a depth of 1.6–1.65 m. The burial contained the skeleton of an adult male (height 1.55 m), lying stretched out on his back with his head to the northwest. The bone preservation was good. Foot bones were absent in situ, but were found in the filling above the left elbow. The left leg was turned outward. The right arm lay along the body with the palm facing up. Beneath the right forearm, a long iron knife in a wooden sheath and an iron awl with remains of a wooden handle and sheath were found. There were no other burial goods. Chronology according to archaeology: 9th c. CE. Chronology according ^{14}C dating: 683–883 cal CE (1232±27BP).

MIDDLE AGES

<u>*Cumans, Cuman culture*</u> *(Половці)*

(Archaeology, text - S. Andrukh, G. Toshev)

Cumans (Polovtsi, Kipchaks) were a medieval nomadic Turkic-speaking people. During the 11th to 14th c. CE, Cumans inhabited the Eurasian steppes from the Irtysh River to the Danube River. As they spread, they displaced the Pechenegs and Khazars. Later, in the 13th c. CE, became a part of the Mongol Empire. We know about Cumans from written sources and archaeological materials. Judging by these, Cumans had close military, political, cultural, and trade contacts with their neighbours, including Byzantium, Kievan Rus, Volga Bulgaria, and others. Their main occupation was cattle breeding. Cumans became a component of the ethnogenesis of Kipchak-speaking peoples, such as the Tatars, Karaites, Nogais, etc.

In the mid-11th c. CE Cumans appeared in the Northern Black Sea region (*266*). In the southern steppes of the Northern Black Sea region, information about Cumans is primarily known from archaeological data. For burials they used both kurgans and ground graves. The most famous complex is a leader's burial in the Chingulsky Kurgan (Zaporizhzhia region). The ordinary people were buried in pits, placing bodies stretched out on their back accompanied by funerary goods, including weapons, everyday objects, jewellery, etc. To the north, in the basin of the Siversky Donets River, the Cuman cities of Balin, Suhrov, and Sharukan are known, which served as settlements for the Cuman khans and were inhabited by a settled agricultural population of Alans. The Alans in these towns engaged in craft production and agriculture for the needs of the Cuman nomads.

Written and archaeological sources indicate that Cuman horde confederations in the 12th c. CE were multi-ethnic. In addition to the Cumans, these unions brought together various steppe and

forest-steppe populations that lived in the region before Cuman arrival. These included Alans and Bulgars from the forest-steppe zone of the Siversky Donets basin, Pechenegs, and Torks from steppe regions who were completely subjugated by Cumans. In the 12th c. CE Cumans underwent a process of forming an ethnic community in which Cumans themselves were the political core around which all other ethnic groups united. The Cumans gave this community its ethnic name. The unity of this political community was primarily determined by a common language, and unified cultural traditions including material culture, funerary rites, religious beliefs, shared epic narratives, and songs (*267*).

According to anthropological data, Cumans, who initially had a distinct South Siberian anthropological type, quickly lost it, dissolving into the larger population of the Northern Black Sea steppes (*267*).

During their first 50 years in the Northern Pontic region, Cumans, like preceding Pechenegs, obtained significant profit from the local Slavic agricultural population through simple plunder, often accompanied by the capture of prisoners for their sale on the slave markets of the Crimea. In the early 13th c. CE, the situation changed, and relative peace and military balance were established between the Cumans and the Slavs. The prolonged coexistence led not only to mutual raids but also to joint allied campaigns and marriages. The nomads actively absorbed the culture of the settled agricultural population, including aspects of daily life, elements of clothing, and household items. The long-term coexistence of Slavs and Cumans resulted in bilingualism among the local population. This process was interrupted by the Tatar-Mongol invasion (Golden Horde). (*268*)

Mamay-Gora *(Мамай-Гора)*

(Archaeology, text – S. Andrukh, G. Toshev)

The archaeological site has been described under the Neolithic Azov-Dnieper culture section.

On the territory of Mamay-Gora, over 60 Cuman burial complexes have been identified. According to the artefacts and coin finds, the ground and kurgan complexes of Mamay-Gora fit into the framework of the 12th–first third of 15th c. CE (*112*, *269*).

Location: 47.432845 N, 34.27259 E. Mamay-Gora tract, Velyka Znamianka village, Vasylivsky District, Zaporizhzhia Region.

Excavations: 1997.

Excavation authors: Svitlana I. Andrukh, Gennadi M. Toshev, Zaporizhzhia National University, Zaporizhzhia, Ukraine.

Storage of anthropological materials: Zaporizhzhia National University, Zaporizhzhia, Ukraine.

Description of samples:

One sample was taken from a Cuman ground burial.

UKR012. *Mamay-Gora, kurgan 162, burial 13.* Excavated in 1997. Inlet burial without artefacts. The depth was 1.35 m. The burial was made in a pit with a niche. The entrance pit was covered with planks and had an elongated oval shape, measuring 2.4 m in length. It was oriented along a north-south line. Along the eastern sloping wall, at a depth of 0.3–0.4 m from the contour fixation level, a step with a width of 0.35 m was observed, sloping down towards the chamber. The

chamber, measuring 2.25x0.7 m, was located on the western side, elongated along the south-north line. The depth from the contour fixation level was 0.7 m. The vault was destroyed in ancient times. The skeleton lay stretched out on its back with the head oriented to the north. A ram bone was found behind the skull. Initially, the burial was dated to the Scythian period, but later the time was defined as medieval. (*110*)

Velyka Znamianka *(Велика Знам'янка)*

(Archaeology, text – S. Andrukh, G. Toshev)

Kurgan group II, consisting of four kurgans, is located on the western outskirts of the village of Velyka Znamenka, 0.2 km south of the Zaporizhia-Kherson highway. These kurgans were excavated in connection with the irrigation system reconstruction project in 1996 by an archaeological expedition from Zaporizhzhia State University. The excavated kurgans contained 30 burials from different time periods. In kurgan 18, measuring 0.45 m in height and 19 m in diameter, seven burials were identified (including two Scythian and five medieval burials). (*270*)

Location: 47.439358 N, 34.335757E. Velyka Znamianka village, Vasylivsky District, Zaporizhzhia Region.

Excavations: 1996.

Excavation authors: Svitlana I. Andrukh, Gennadi M. Toshev*,* Zaporizhzhia National University, Zaporizhzhia, Ukraine.

Storage of anthropological materials: Zaporizhzhia National University, Zaporizhzhia, Ukraine.

Description of samples:

One sample was taken from a Cuman period burial.

UKR027. *Velyka Znamianka, kurgan 18, burial 1.* Inlet burial. Excavated in 1996. The burial was robbed and completely destroyed during the robbery. It was located at a depth of 0.7 m. The pit had a rectangular shape, measuring 2.35x1.1 m, elongated in a west-east direction. In the southeast part of the pit, the remains of a wooden coffin were preserved. Above the burial, at a depth of 0.05–0.15 m, a horse's skull was found, at the base of which was a bone buckle and fragments of reins. At the same depth, small fragments of an iron sword were found. There were two bone clusters near the southern wall of the pit. The eastern cluster contained a skull, ulnas and radiuses, pelvis, femurs, and vertebrae. In the western cluster, located 0.4 m away, tibia bones were arranged. Many small fragments of a sword were found on the bones. To the north of the skull, a silver plaque was found. Under the western cluster, gold foil, two bone inlays, two bronze buttons, fragments of a sword, a fragment of an item with rivets, a fragment of a ring, three iron arrowheads in a decayed leather case, and a bone cheek-piece were found. Chronology according to archaeology: 12th c. CE.

Kumy *(Куми)*

(Archeology – I. Shramko; text – I. Shramko, S. Zadnikov)

The archaeological site has been described under the Early Iron Age Cimmerian culture section.

The kurgan was found in 2010 during a scientific archaeological expertise (*137*). The site where the kurgans were located is situated in the fields of the Krasnograd Research Station, near Kumy village in the Krasnograd District of the Kharkiv Region. The kurgan, located on the edge of a watershed plateau on the right bank of the Berestovaya River (right tributary of the Orel River), consists of nine kurgans of varying sizes, most of which were plowed and are scarcely visible in the field. (*139*)

Location: 49.3246500 N, 35.3687167 E. Kumy village, Krasnograd district, Kharkiv region, Ukraine.

Excavations: 2010.

Excavation authors: Iryna Shramko, Museum of Archaeology of V.N. Karazin Kharkiv National University, Kharkiv, Ukraine.

Storage of anthropological materials: Museum of Archaeology of V.N. Karazin Kharkiv National University, Kharkiv, Ukraine.

Description of samples:

One sample was taken from a Cuman ground burial.

UKR056. *Kumy, kurgan 1, burial 1b.* A damaged complex containing the remains of two destroyed burials dated to the Late Bronze Age (2nd period of the Berezhnevo-Mayivska Zrubna Culture) (1a) and the Middle Ages (1b) (the inlet burial). It is impossible to determine the sizes of the burial pits and the level of their bottom because these complexes have been disturbed by burrowing animals. The human bones and burial goods were displaced from their original places and were found in mixed soil layers. Fragments of Bronze Age ceramics were found among the artefacts from the late Middle Ages. It was difficult to establish the orientation of the burials and the skeletons. An iron knife, remains of heavily corroded iron stirrups, and a fragment of a bone plate date to the Middle Ages. In the southern sector of the pit, at a depth of 0.95 m, a human skull (without the lower jaw) and clavicle bones were found. Chronology according to archaeology: 9th–11th c. CE. Chronology according to ^{14}C dating: 991–1149 cal CE (1010±25 BP).

<u>*Kyivan Rus and Golden Horde: Slavs and eastern nomads*</u> *(Київська Русь і Золота Орда)*

(Text – V. S. Tersky, S. Zadnikov)

Starting from the 8th c. CE, Slavic tribes in Eastern Europe began to unite into political alliances, and in the 9th c. CE the state of Kyivan Rus was formed, with its center in the city of Kyiv, which emerged in the 6th c. CE. From the second half of the 11th c. CE, Kyivan Rus experienced a period of feudal fragmentation. The state disintegrated into separate principalities – independent state structures with their own elites, trade and political relations with neighbours, and other local features that determined the direction of their historical development.

From the mid-8th to the last third of the 10th c. CE, Slavic tribes in the southeast were economically dependent on the Khazars, carriers of the Saltiv culture. From the second half of the 13th to the last quarter/end of the 14th c. CE, the development of Slavic statehood was complicated by the presence of eastern nomads, Turks and Mongols (Golden Horde).

The main Slavic burial rite until the 9th c. CE was cremation. However, after the adoption of Christianity, cremation gradually gave way to the rite of inhumation. In the 9th–10th c. CE, a

kurgan culture known as the "warrior culture" emerged, belonging to representatives of the Slavic military-political elite. Anthropological material from this period is very poor.

Burials of Golden Horde representatives in the forest-steppe were usually found accidentally and typically were without artefacts, making their identification problematic. Large separate burial grounds in the forest-steppe have not been found yet. In the steppe, the most well-known landmark is Mamay-Surka. Many burials have been uncovered in Mamay-Gora, as well as in other steppe sites (*271*).

Zvenyhorod *(Звенигород)*

(Archaeology – H. Vlasova, V. Shelomentsev-Tersky, text – S. Tersky)

The landmark of Zvenyhorod is related to the Slavic culture of the Kingdom of Galicia-Volhynia (Kingdom of Ruthenia), which emerged as one of political centres after the collapse of Kyivan Rus and included western Ukraine with some areas in Belarus, Poland, Moldova, and Lithuania.

The Galician group of landmarks is represented by cities, fortified and unfortified settlements, and inhumation burials. From the 10th to the 12th c. CE, kurgans practically were not used in the burial rite of Eastern Carpathians. The main type of burial is ground pits, and occasionally stone sarcophagi (predominantly in princely churches). Among ground burials, there were ones under stone plates, which indicates more southern customs. The traditional skeletal position is supine, with the head oriented mainly westward.

Zvenyhorod as a city with a princely court arose at the end of the 11th c. CE from several local unfortified settlements of 10–11 c. CE. The city is located near the Main European Watershed, which is conventionally considered to be the border between Eastern (Carpathian) Croats and Volhynians (Buzhans). These Slavic tribes settled the basins of the upper Western Bug River and the upper Dniester River in early medieval times. Therefore, the local population in the 12th and 13th c. CE could have descendants of these two tribal groups.

The Zvenyhorod fortifications covered approximately 50 hectares. Within this area, the remains of three churches and nine cemeteries have been studied, and another seven cemeteries were found within a radius of up to 2 km. A significant agglomeration developed around the city, extending 15 km from west to east and at least 7 km from north to south. Until 1241, this area may have housed at least 12 churches and five monasteries, each with their mainly small cemeteries. The two large (city-wide) cemeteries of the 11th–12th c. CE, located at Mount Hoyeva Hora and Zahumenki, have been investigated most thoroughly. All the burials found in Zvenyhorod are inhumations in grave pits. (*272–274*)

Location: 49.73363 N, 24.247629 E. Zvenyhorod village, Lviv district, Lviv region.

Excavations: 1960, 1962.

Excavation authors: Halina Vlasova, Volodymyr S. Shelomentsev-Tersky, Lviv Historical Museum, Lviv, Ukraine.

Storage of anthropological materials: Lviv Historical Museum, Lviv, Ukraine.

Description of samples:

One sample was taken for aDNA analysis.

UKR166. *Squares 12 and 13 К (№ 23353), cultural layer I-II, Excavation No. 1.* The burial was found at the depth of 30 cm in the northwestern part of Okolny town. The burial was associated with the Slavic population on the border between Volynians and White Chroats. Chronology according to archaeology: the first half of the 13th c. CE.

Donets hillfort *(Донецьке городище)*

(Archaeology – B. Shramko, text – I. Shramko, S. Zadnikov)

The Donets hillfort is related to the Slavic culture of Kyivan Rus in the Siversky Donets basin in the Golden Horde period (13th–14th c. CE). The territory of the upper Siversky Donets River was on the border with nomadic tribes, which influenced the population's lifestyle and the development of settlement infrastructure. Archaeological excavations revealed dwellings, workshops, farm buildings and numerous artefacts from two periods of the settlement's existence. During the early Middle Ages (8th–10th c. CE), fortified settlements of early Slavs emerged here, later evolving into cities of the Kyivan Rus (10th–13th c. CE).

In the 11th c. CE, the territory belonged to the Principality of Pereyaslavl. From the 10th to the 13th c. CE, the city played an important military-defensive role on the southern border of the ancient Rus state and had significance for international trade along ancient overland and water routes along the Donets and Don rivers (*275*). In 1239, during the Tatar-Mongol invasion, the city, abandoned by residents, was burned (*275*).

During the Mongol invasion, the city was abandoned by its population. Some parts of the city were used as a burial ground, but no cultural artefacts have been documented from the second half of the 13th–14th c. CE. On the territory of the settlement, burials dated to this period were found (*276*, *277*). At that time the southeastern territories of the state fell under the rule of the Golden Horde.

Location: 49,921928N, 36,192856 E. Kharkiv city, Novobavarsky district.

Excavations: 1960.

Excavation authors: Borys A. Shramko, V. N. Karazin Kharkiv National University.

Storage of anthropological materials: Museum of Archaeology of V.N. Karazin Kharkiv National University.

Description of samples:

Five samples from excavation 4 of the Donets hillfort belonging to the Golden Horde period were taken. Three samples yielded a sufficient amount of DNA for analysis.

UKR068. *Donets hillfort. Burial 1, field number 1132/IV-1960.* The burial was found at a depth of 0.4–0.45 m. The pit had a rectangular shape, with a length of 1.7–2.0 m and a width of 0.55–0.6 m. Remains of wood were found along the wall. In the burial, there was a skeleton that was osteologically estimated to be of a middle-aged man (but genetically female). The skeleton laid out on the back with the head oriented to the west. The right arm was placed on the chest, the left on the abdomen. There were no artefacts. Chronology according to archaeology: the end of 13th–beginning of 14th c. CE.

UKR069. *Donets hillfort, Burial 4.* The burial was found at a depth of 0.4–0.45 m. The pit had a rectangular shape, with a length of 1.7–2.0 m and a width of 0.55–0.6 m. An elderly man was

buried, laid out in an extended position on the back, with the head oriented to the west. There were visible traces of two strong blows on the skull. The right arm was placed on the chest, the left on the abdomen. There were no artefacts. Chronology according to archaeology: the end of 13th–beginning of 14th c. CE.

UKR070. *Donets hillfort, Burial 2, field number 1150/IV-1960.* The burial was found at a depth of 0.4–0.45 m. The pit had a rectangular shape, with a length of 1.7–2.0 m and a width of 0.55–0.6 m. A very elderly individual with toothless jaws, where tooth sockets in the gums long healed, was buried. The skeleton lay on the back, with the head oriented to the west. The right hand was on the chest, the left on the abdomen. No artefacts were found. The burial pit was located above the household pit No. 81 with filling from the period of Kyivan Rus. Chronology according to archaeology: the end of 13th–beginning of 14th c. CE.

Kumy *(Куми)*

(Archaeology, text – I. Shramko, S. Zadnikov)

The archaeological site has been described under the Early Iron Age Cimmerian culture section.

Historians associate the decline of life in certain ancient Rus settlements to the time of the Mongol Golden Horde invasion (*275*, *277*). The buffer zone of the Golden Horde in the forest-steppe of the Dnieper left bank covered an area of about 200 km in the meridional direction, and in the Siversky Donets basin, its northern border ran somewhat south of Kharkiv (*278*). Numerous monuments from the post-Mongol period were found in this zone (*139*, *279–281*). Materials from the Kumy site significantly expand our understanding of the life of the population in the middle reaches of the Siversky Donets in the post-Golden Horde period, challenging the notion of a long existence of an uninhabited "Wild Field" in this territory. It is more likely that cohabitation occurred in the post-Golden Horde period within the contact territory of both sedentary Slavic farmers and steppe nomads.

Location: 49.3246500 N, 35.3687167 E. Kumy village, Krasnograd district, Kharkiv region, Ukraine.

Excavations: 2010.

Excavation authors: Iryna Shramko, Museum of Archaeology of V.N. Karazin Kharkiv National University.

Storage of anthropological materials: Museum of Archaeology of V.N. Karazin Kharkiv National University.

Description of the samples:

One sample was taken from Kumy ground burial of the Golden Horde period.

UKR063. *Kumy, kurgan 1, burial 3.* An inlet burial attributed to medieval Golden Horde nomads. The burial pit was found at a depth of 0.80 m, at the level of loam. The pit had a rectangular shape with rounded corners, and its longer axis was oriented in the west-east line. There was a small ledge in the southern part. The skeleton of a child was found, which lay on the back in an extended position with the head facing west. The right arm was extended along the body, the left arm was slightly bent at the elbow, likely the hand was resting on the pelvic bones. The legs were extended

and brought together at the feet. No artifacts were found in the burial. Chronology according to archaeology: 14th c. CE.

Ploske *(Плоске)*

(Archaeology - V. Mikheev, text – I. Shramko, S. Zadnikov)

The ground burial near Ploske village belonging to the settlement of the Saltiv culture is placed on the elevated right bank of the Siversky Donets River in the immediate vicinity of the Mayaki hillfort. The Mayaki hillfort was a fortified settlement of the early medieval period (9th–10th c. CE). On the edge of the settlement, burial ground No. 4 of the Golden Horde period was explored (*234*). The ground burial 9 was found in excavation № 9, in the eastern part of the settlement. According to archaeological data, it was preliminarily dated to the 13th–14th c. CE (*282*).

Excavation authors: Volodymir K. Mikheev, V. N. Karazin Kharkiv National University.

Excavations: 1965.

Storage of anthropological materials: Museum of Archaeology of V.N. Karazin Kharkiv National University, Kharkiv, Ukraine.

Location: 48.940698 N, 37.666706 E. Mayaki village, Kramatorsk district, Donetsk region.

Description of samples:

One sample was taken for aDNA analysis.

UKR074. *Ploske, Burial 9, excavation 9.* The pit was found at a depth of 0.85 m. The human skeleton was badly damaged. It lay on the back in an extended position, with the head oriented to the west. To the right of the skull, there was a fragment of a thick-walled clay vessel. To the left side of the skull, a conical bone amulet with two holes for hanging was found. (*282*)

Mamay-Surka *(Мамай-Сурка)*

(Archaeology – G. Toshev, S. Andrukh)

The burial site of Mamay-Surka is located on the high left bank of the Kakhovka Reservoir, west of the village of Velyka Znamianka in Vasylivka District, Zaporizhia Oblast, Ukraine. It has been under excavation since 1989 by an archaeological expedition from Zaporizhzhia National University. By 2006, 1,162 burials of the 14th c. CE had been found there, within a total area of 7,166 m^2. The individuals were buried in pits, stretched out on their backs with the heads oriented to the west. The burial artefacts consisted of jewellery, household items, and rare findings of coins. According to anthropology, the site represents a mixed settled population of post-Cuman time (*283–285*).

Location: 47.430345N, 34.280355E. Ground burial Mamay-Surka 14th c. near the Velyka Znamianka village, Vasylivsky District, Zaporizhzhia Region.

Excavations: 1997.

Excavation authors: Svitlana I. Andrukh, Gennadi M. Toshev*,* Zaporizhzhia National University, Zaporizhzhia, Ukraine.

Storage of anthropological materials: Zaporizhzhia National University, Zaporizhzhia, Ukraine.

Description of samples:

Two samples were taken for aDNA analysis, one of them yielded enough DNA to be included in subsequent analysis.

UKR028. *Mamay-Surka, burial 188*. The burial was found at a depth of 1.40 m. The contours of the pit were not discernible. Th filling material was black soil. The skeleton of a woman aged 30–35 years lay stretched out on the back witeh the head oriented to the southwest. The arms were bent at the elbows at a right angle, and the hands lay on the pelvis. Between the right elbow and the ribs, the bones of a child were found. On the left side near the temple bone, an earring was found. Among the bones, an iron needle was found. Chronology according to archaeology: 14th c. CE.

Nogai (Ногаї, ногайці)

(Archaeology, text – S. Andrukh, G. Toshev)

The Nogai Horde was the successor of the Mangit Yurt. It arose on the territory of modern northern Kazakhstan at the end of the 14th c. and the beginning of the 15th c. CE amid the disintegration of the Golden Horde. It was formed by late nomads from the Lower Volga and the Ural regions. The first waves of Mangit people in the Northern Black Sea region are documented at the beginning of the 15th c. CE. However, at that time, they did not have significant ethno-political power.

In the steppe regions of the Northern Black Sea, in the early stages, only Nogai kurgans are known, such as the Balkivsky Kurgan, Berdiansky Kurgan, Mamay-Gora burial ground. The Mamay-Gora burial ground had around 200 burials. The individuals were placed in pits or ledge-pits, stretched out on their backs with their heads oriented to the west. The findings of coins in these burials are a basis for dating this largest burial ground in the Northern Black Sea region to the second half of the 15th c. CE.

The situation changes from the mid-16th c. CE when a significant number of Nogais migrated to the Northern Black Sea region. They form autonomous hordes, including the Yedisanska, Yedichkulska, Jambuylutska, and Budzhakska (*32*, *286*, *287*).

Mamay-Gora (Mamai-Hora) *(Мамай-Гора)*

(Archaeology, text – G. Toshev, S. Andrukh)

The archaeological site has been described under the Neolithic Azov-Dnieper culture section.

Location: 47.432845 N, 34.27259 E. Mamay-Gora tract, Velyka Znamianka village, Vasylivsky District, Zaporizhzhia Region.

Excavations: 2006–2008, 2012–2013.

Excavation authors: Svitlana I. Andrukh, Gennadi M. Toshev, Zaporizhzhia National University, Zaporizhzhia, Ukraine.

Storage of anthropological materials: Zaporizhzhia National University, Zaporizhzhia, Ukraine.

Description of samples:

Nine samples affiliated to Nogais were taken from Mamay-Gora (*271*, *286*, *287*), DNA was extracted from eight and seven of them yielded enough DNA to be included in subsequent analysis.

UKR016. *Mamay Gora, object 242, burial 1.* Excavated in 2012. The burial pit was found at a depth of 0.65 m. It had an oval shape, measuring 2.5x0.6 m, with a depth of 0.45 m. The pit was filled with black soil. The skeleton was lying stretched out on the back with the head oriented to the west. The right arm was placed along the body, the left arm was bent at the elbow with the hand on the pelvis. (*288*). Chronology according to archaeology: 15th c. CE.

UKR017. *Mamay Gora, object 266, burial 1.* Excavated in 2013. Found at a depth of 0.9 m. The pit had an elongated oval shape (2.25x0.7x0.5 m) and a depth of 0.4 m. It was filled with black soil mixed with clay. The skeleton of a 50–55-year-old man was lying stretched out on his back with the head oriented to the west (slightly turned to the south). The arms were placed along the body. (*288*). Chronology according to archaeology: 15th c. CE.

UKR018. *Mamay Gora, object 206, burial 1.* Excavated in 2008. The rectangular shaped pit was found at a depth of 0.85 m and measured 2.3x0.85 m. The filling was mixed. In the northern part of the pit, remains of wood were found. Along the southern wall, at a depth of 0.2 m from the contour of the pit, there was a step about 0.25 m wide. The total depth of the pit was 0.4 m. The burial chamber had a size of 2.3x0.6 m. The skeleton of an adult individual lay stretched out on the back with the arms placed along the body. The knees were slightly bent to the right. (*289*). Chronology according to archaeology: 15th c. CE.

UKR020. *Mamay Gora, object 260, burial 1.* Excavated in 2013. Found at a depth of 0.9 m. The pit had an elongated oval shape, measuring 2.3x1x0.8 m, with a depth of 0.7 m. The pit filling was mixed. The skeleton of a 40–45-year-old man lay stretched out on the back with the head oriented to the west (slightly turned to the south). The right arm was bent at the elbow, with the hand on the pelvis, the left arm was placed along the body. (*288*). Chronology according to archaeology: 15th c. CE.

UKR021. *Mamay Gora, object 268, burial 1.* Excavated in 2013. The burial was found at a depth of 1.05 m and was made in a pit with a niche. The entrance pit measured 2.1x0.6 m and had a depth of 0.1 m from the contour fixation level. The filling of the pit was mixed. Below the level of the entrance pit, there was an oval-shaped burial chamber measuring 2.2x0.6 m. The skeleton of a 45–50-year-old woman was lying stretched out on the back with the head oriented to the east (slightly turned to the south). The left arm was bent at the elbow at a right angle, with the hand on the pelvis, while the right arm was placed along the body. (*288*). Chronology according to archaeology: 15th c. CE.

UKR022. *Mamay Gora, object 194, burial 1.* Excavated in 2007 The pit was found at a depth of 0.85 m. It had an almost rectangular shape, with a length of 2.15 m and a width of 0.75 m. At a depth of 0.2 m from the pit contour, along the northern wall, a step up to 0.18 m wide was found, and in the southern wall, there was a niche up to 0.1 m wide. The bottom of the burial chamber was 0.08 m below the step. The skeleton of a woman lay stretched out on the back with the head oriented to the west. The arms were placed along the body. The skull was slightly turned with the facial part to the left. There were wooden remains under the skeleton. (*288*). Chronology according to archaeology: 15th c. CE.

UKR024. *Mamay Gora. object 189, burial 1.* Excavated in 2006. The burial outline was found at a depth of 0.84 m. It was made in a ledge-pit. The upper pit had an oval shape, measuring 2.1x0.9 m, with lateral pits that were 0.2–0.3 m wide and 8 cm high. The lower pit was also oval and measured 2.14x0.43 m with a depth of 0.28 m. The skeleton was lying stretched out on its back

with the head oriented to the west. The arms were placed along the body. (*288*). Chronology according to archaeology: 15th c. CE.

EARLY MODERN PERIOD

Ukrainian Cossack Culture, lower Desna, left bank of Dnipro, Slavs (Українське козацтво, Лівобережжя, нижня Десна)

(Archaeology – V. Skorokhod, text – V. Skorokhod, Y. Sytyi, V. Zhyhola)

The 17th and 18th c. CE in Ukraine can be considered as the early modern period. Permanent settlements were represented by fortresses and open settlements. In the 18th c. CE, fortresses were built in the Dutch manner, with bastions located outside of the fortifications' line. The urban constructions on the Left Bank of Dnipro in the 17th c. CE were primarily wooden, but in the 18th c. CE brick buildings appeared next to wooden ones. Traditional dwellings in towns were represented by houses with non-residential basements and the above-ground living floor, which was heated with tiled stoves.

The future resident received a building site within the fortress under the condition of defending the fortifications and participating in military campaigns with own weapons and means of transportation. In addition, residents received land outside the city for cultivating to earn income for living. Taxes were paid from these profits. Both peasants and townspeople were engaged in agriculture, and various crafts were well developed.

As in any city, various ethnic groups lived together. The main population consisted of Ukrainians, but there were also Poles, Germans, people from the Turkish steppe, Russians, Belarusians, Lithuanians, and others. (*290*)

Vypovziv *(Виповзів)*

Vypovziv Hillfort (village of Vypovziv, Chernihiv district, Chernihiv region) is a multi-layered archaeological site. It contains artefacts from the late Neolithic-Chalcolithic period (Kyiv-Pechersk culture and sites of the Pustynka-V type, which were part of the Dnipro-Donetsk ethno-cultural community 7000–4000 BCE), the Bronze Age (Middle Dnipro and Sosnytska cultures, 3000–1500 BCE), and the early Slavic period in the early Iron Age (1st–3rd c. CE).

The most intensive activity at the site took place from the 10th to the 13th c. CE, when it was inhabited by the population of Kievan Rus. Later, in 17th–18th c. CE, there was a wooden Christian church and a cemetery around it. The cemetery was excavated in 1889 and then in 2013–2014 by V. L. Berenshtam and V. M. Skorokhod. In total, 16 burials have been investigated to date, all of them were carried out according to the Christian rite, in graves and in wooden coffins. Bodies were put in an extended position on backs with heads oriented to the west. Burials also could be oriented parallel to the long axis of the church. Some burials included accompanying items such as coins and rings. (*291–293*)

Location: 50.945951 N, 30.8039 E. Vypovziv village, Chernihiv district, Chernihiv region, Ukraine.

Excavations: 2013.

Excavation authors: Viacheslav M. Skorokhod, Institute of Archaeology of the National Academy of Sciences of Ukraine, Kyiv, Ukraine.

Storage of anthropological materials: Institute of Archaeology of the National Academy of Sciences of Ukraine, Kyiv, Ukraine.

Description of samples:

Two samples of the early modern period were taken for aDNA analyses.

UKR164. *Vypovziv, burial 12.* A woman aged 25–30 years old. The grave was approximately 0.5 m wide and studied to a length of 1.63 m. The grave was oriented along the east-west axis with a slight deviation to the south. Decayed wooden planks from a coffin were found within the grave. The skeletal remains of an adult individual were in extended position on the back, with the head facing the southwest. The hands were folded on the abdomen. The preservation of the bones was satisfactory. On the one hand, two rings made of coloured metal with square and round-shaped stone inserts were found. The bottom of the grave was at a depth of 0.2 m from the level of grave identification. The individual was identified as Ukrainian based on Orthodox Christian burial rite. Chronology according to archaeology: 18th c. CE.

UKR165. *Vypovziv, burial 13*. A woman aged 30–40 years old. The grave was approximately 0.4 m wide and studied to a length of 0.83 m. Decayed wooden planks from a coffin, about 0.35 m wide and up to 0.2 m in height, were identified. The upper part of skeletal remains of an adult individual were found, that lay in an extended position on the back, with the head facing the southwest. The preservation of bones was satisfactory. The bottom of the burial was at a depth of 0.25 m from the upper contour of the pit. The individual was identified as Ukrainian based on Orthodox Christian burial rite. Chronology according to archaeology: 18th c. CE.

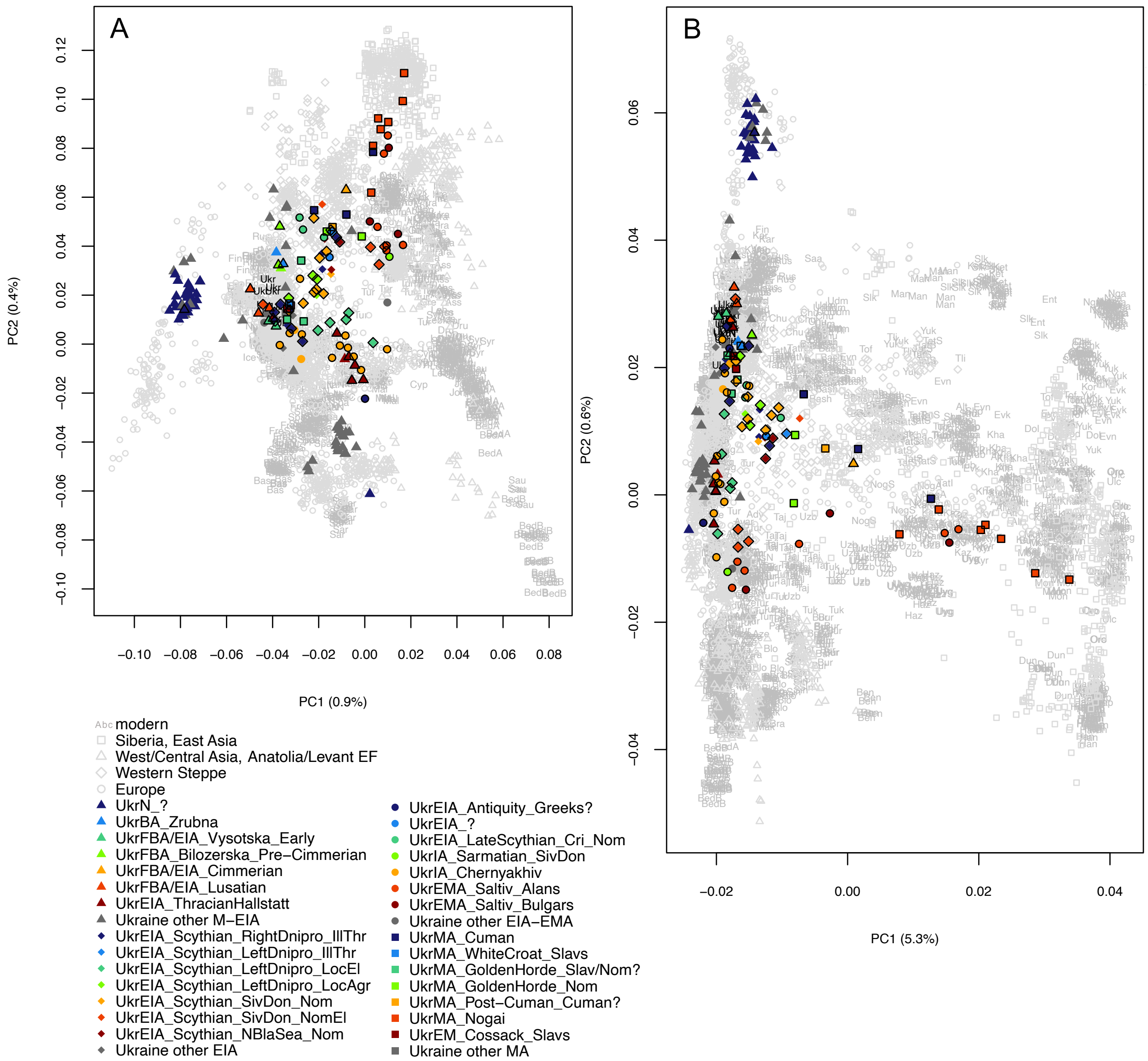

Fig. S1.

Full principal component analysis results. Principal component analysis results of modern (**A**) West Eurasians, (**B**) Eurasians with ancient individuals projected onto the first two components (PC1 and PC2). Newly reported individuals are indicated with a black outline. Modern Ukrainians are shown in black.

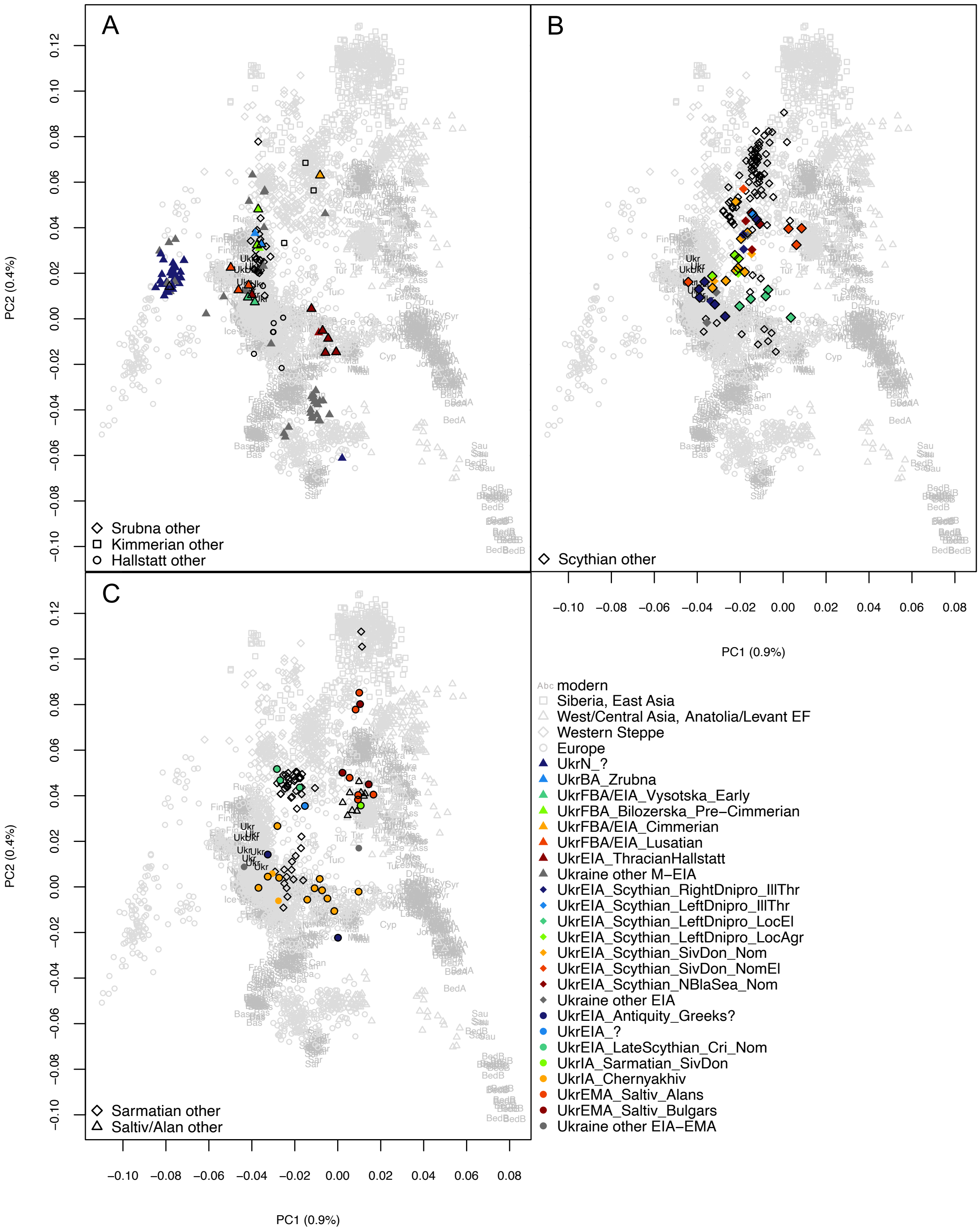

Fig. S2.

Principal component analysis results with relevant previously published individuals outlined. Principal component analysis results of modern West Eurasians with ancient individuals projected onto the first two components (PC1 and PC2). Ukrainian and previously published groups from (**A**) Late Bronze Age and pre-Scythian Iron Age (3,000–700 BCE), (**B**) the Scythian period of Early Iron Age (700–300 BCE), (**C**) post-Scythian Iron Age until Early Middle Ages (400 BCE–900 CE). Newly reported individuals are indicated with a black outline. Modern Ukrainians are shown in black.

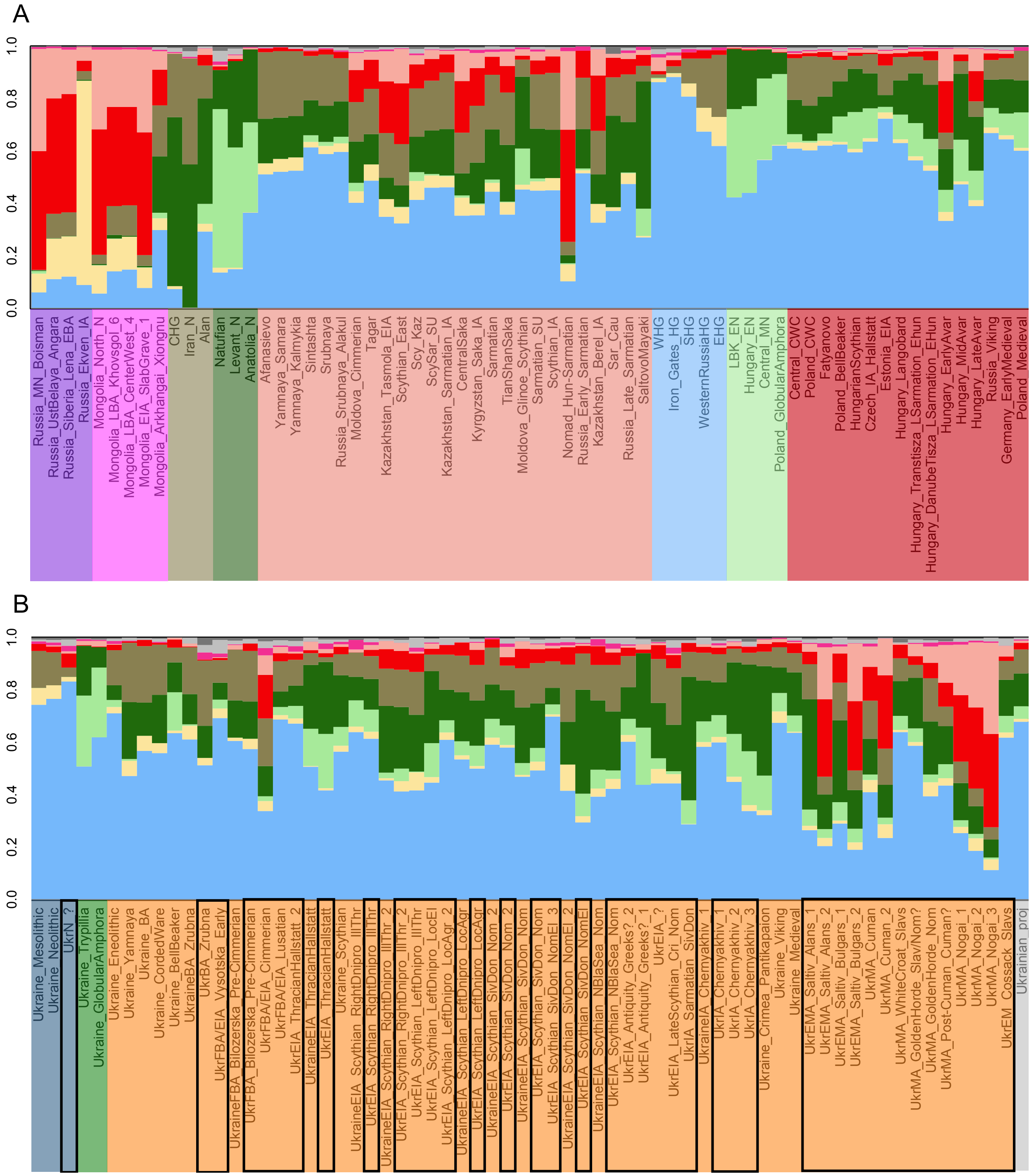

Fig. S3.

Subset of ADMIXTURE analysis results with ancient individuals projected onto modern structure. Population averages of (**A**) Eurasia-wide, (**B**) Ukrainian ancient groups at K10. Purple – Siberia, magenta – East Asia, olive – West/Central Asia, dark green – Anatolian/Levantine early farmers, salmon – Western Steppe, light blue – European hunter-gatherers, light green – European early farmers, dark red – European post-steppe migration groups, dark blue – Ukrainian hunter-gatherers, bright green – Ukrainian early farmers, orange – Ukrainian post-steppe migration groups.

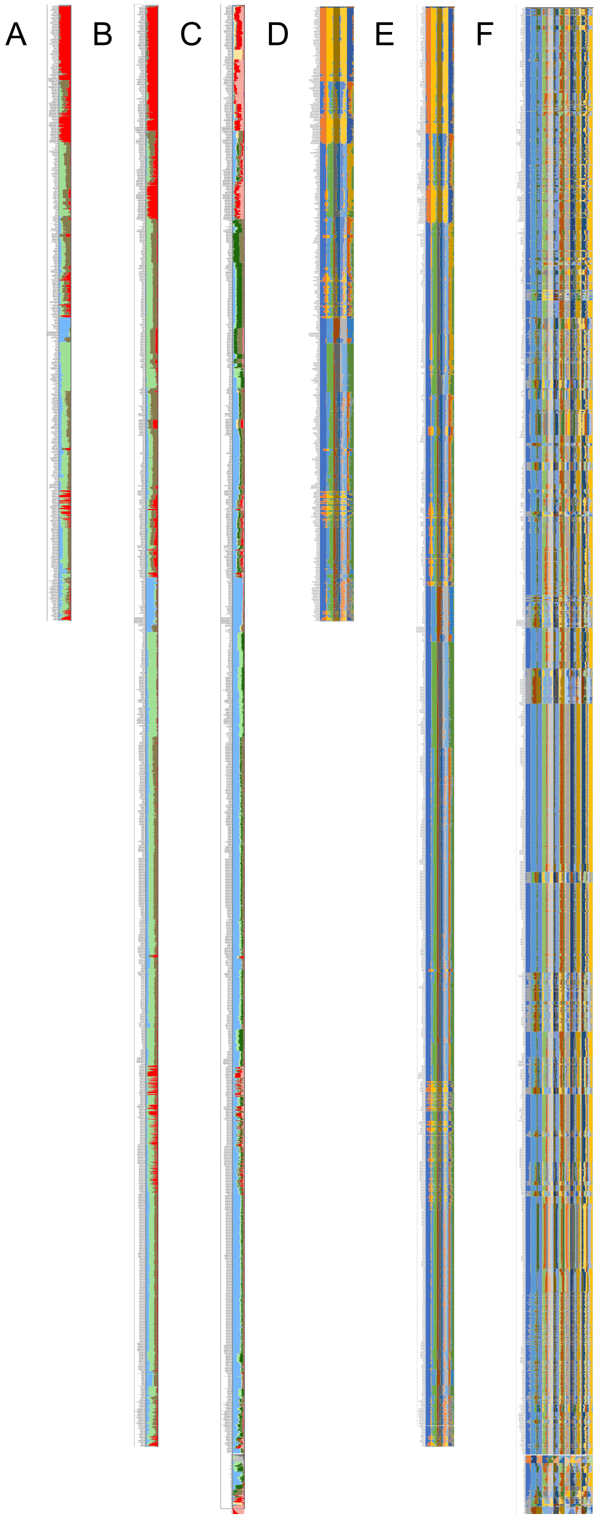

Fig. S4.
Full ADMIXTURE analysis results. (**A**) ancient individuals at K4, (**B**) ancient individuals projected onto ancient structure at K4, (**C**) projected ancient individuals and modern population averages at K10, (**D**) ancient individuals at K2 to K6, (**E**) ancient individuals projected onto ancient structure at K2 to K6, (**F**) projected ancient individuals and modern population averages at K3 to K14.

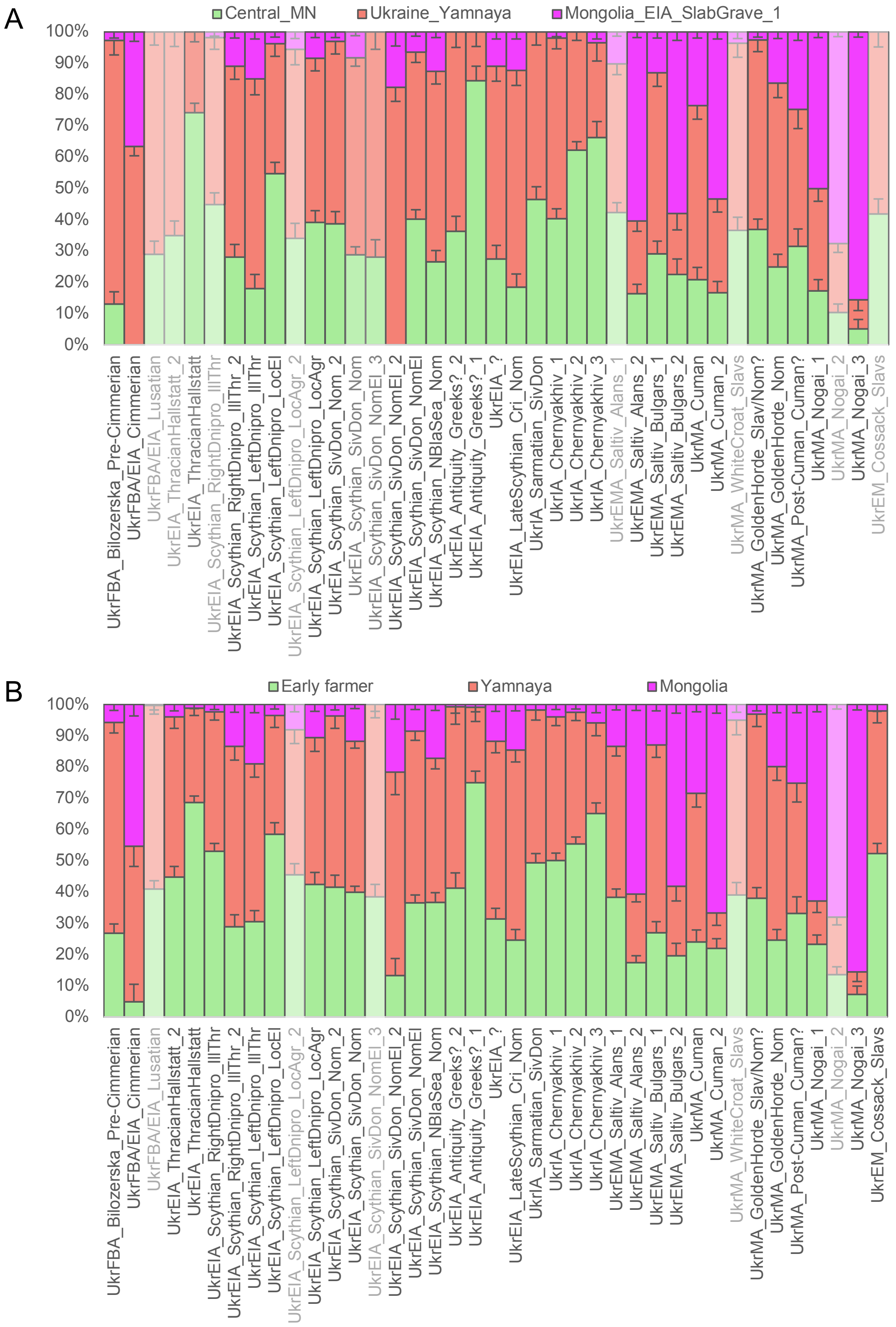

Fig. S5.

Additional qpAdm admixture modelling results. Distal qpAdm models of admixture between (**A**) Central_MN, Ukraine_Yamnaya and Mongolia_EIA_SlabGrave_1, (**B**) a European early farmer group, a Yamnaya group and a Mongolian group resulting in the highest p value for each target group, tested using the autosomal positions of the 1240K dataset. Models with non-significant p-values (p<0.05) are semi-transparent.

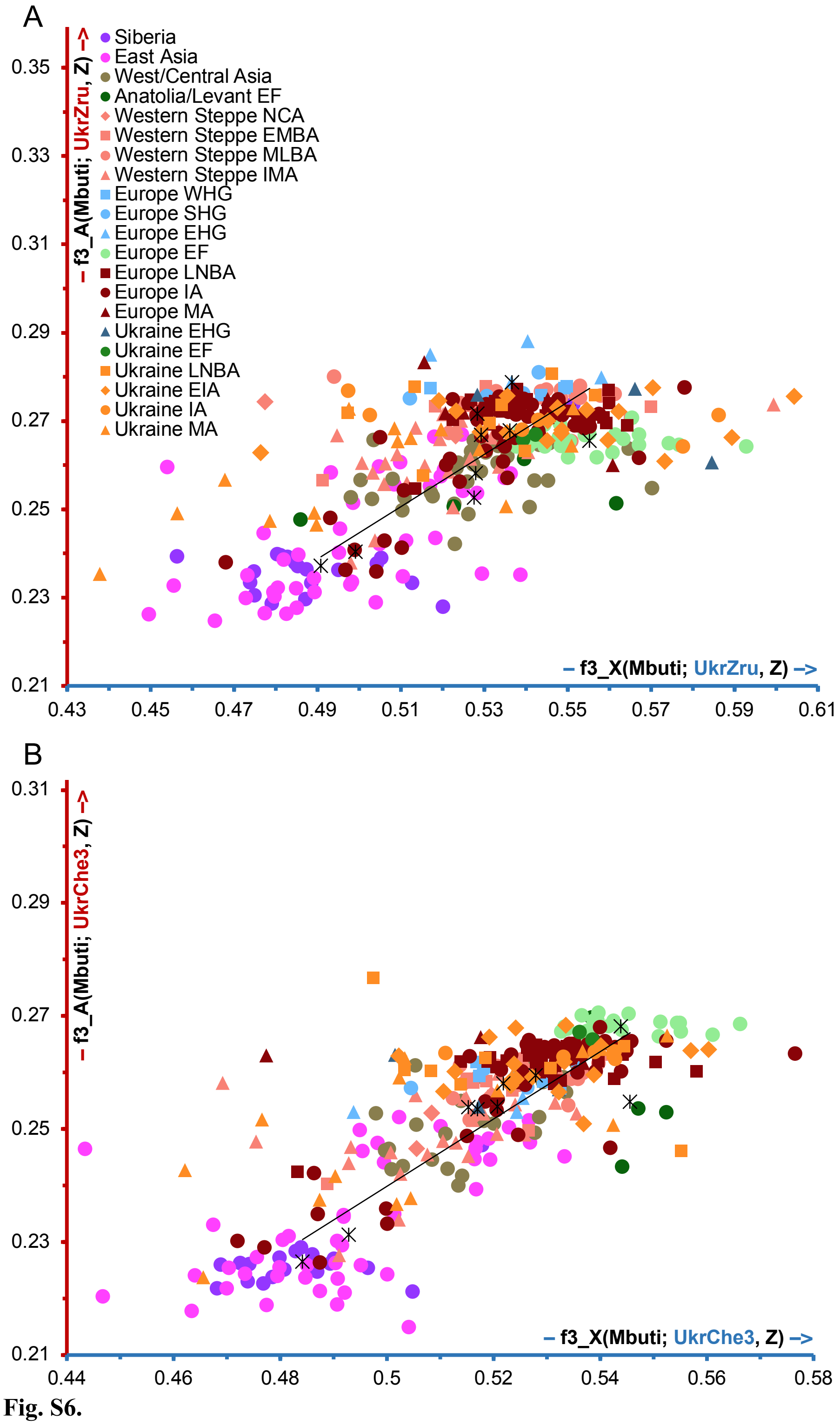

Fig. S6.

Outgroup f3 statistics' results of comparisons with ancient populations. Outgroup f3 statistics' values of form f3(Mbuti; Ukrainian population, ancient) using chr X/autosomal SNPs of the 1240K dataset. Ukrainian population: (**A**) Zrubna, (**B**) Chernyakhiv_3. EF – early farmers; NCA – Neolithic/Copper Age; EMBA – Early/Middle Bronze Age; MLBA – Middle/Late Bronze Age; IMA – Iron/Middle Ages; HG – hunter-gatherers, W – Western, S – Scandinavian, E – Eastern; LNBA – Late Neolithic/Bronze Age; IA – Iron Age; MA – Middle Ages. The average values for groups are shown with black asterisks. The trendline for the black asterisks is shown in black.

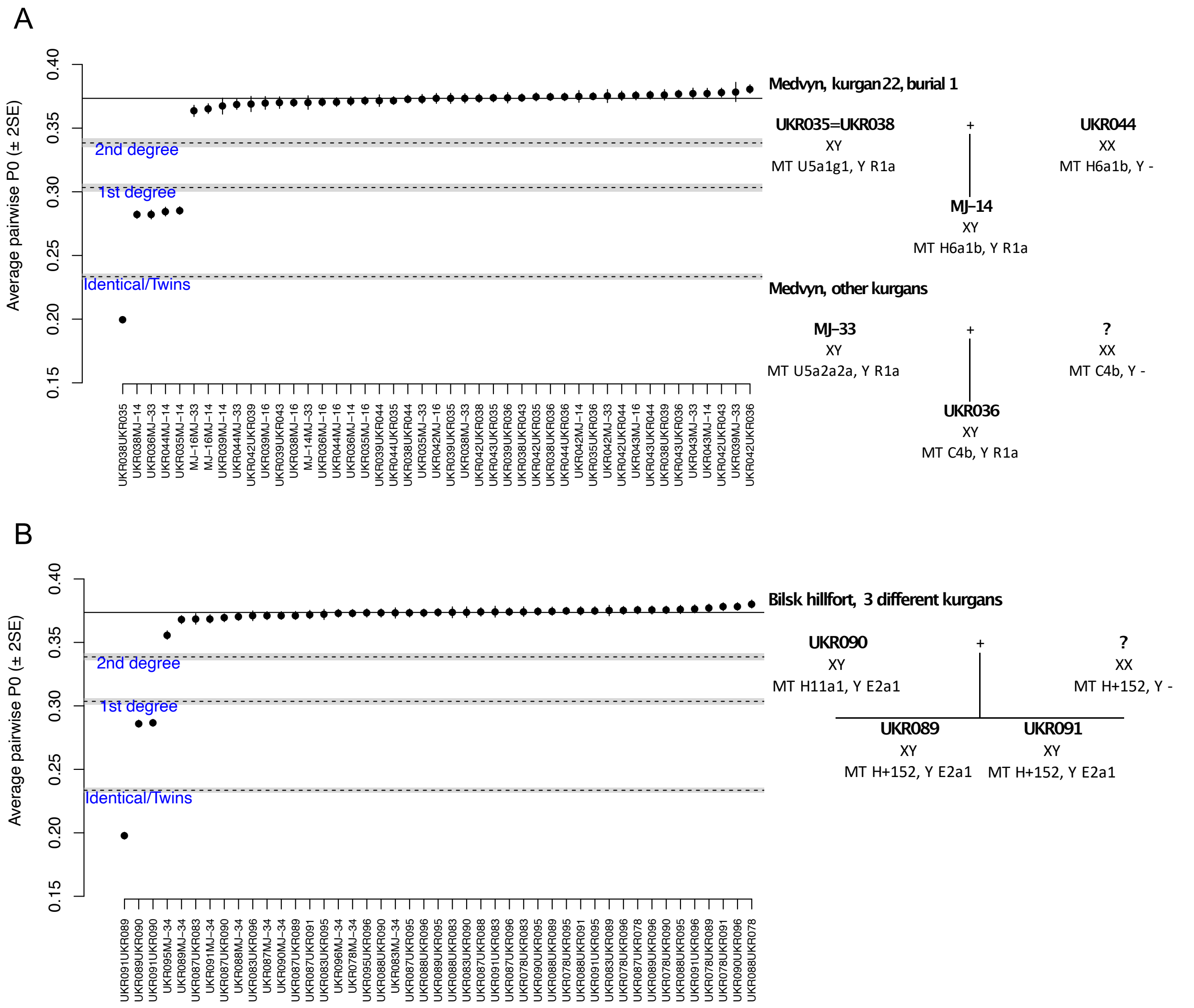

Fig. S7.
Kinship test results. (**A**) right bank of Dnipro Scythians' kinship test results, (**B**) left bank of Dnipro Scythians' kinship test results.

Data S1. (separate file)
Information about the individuals of this study.
Summary of the geographic and archaeological context and general genetic results.

Data S2. (separate file)
Y chromosome informative positions for haplogroup determination.
Haplogroup informative Y chromosome positions with the ancestral and derived alleles, associated haplogroup, marker ID and state (. – missing, 0 – ancestral, 1 – derived) for the male individuals of this study. The assigned haplogroup and average Y chromosome coverage of each individual are indicated above individual IDs.

Data S3-S4. (separate file)
Modern and ancient comparison populations used in different analyses.
Combined as tabs in Microsoft Excel spreadsheet:
Data S3. "ModPops"
Modern comparison populations used in West Eurasian and Eurasian principal component analysis (PCA WE, PCA Eurasia), modern ADMIXTURE (ADM HO), outgroup f3 and f4 statistics (f3, f4).
Data S4. "AncPops"
Ancient comparison populations used in West Eurasian and Eurasian principal component analysis (PCA WE, PCA Eurasia), modern and ancient ADMIXTURE (ADM HO, ADM anc), outgroup f3 and f4 statistics (f3, f4).

Data S5-S6. (separate file)
f4 statistics' results.
Combined as tabs in Microsoft Excel spreadsheet:
Data S5. "f4_summary"
Summary of f4 statistics' results of comparisons with ancient populations using the autosomal positions of the 1240K dataset.
Data S6. "f4_all"
All f4 statistics' results of comparisons with ancient populations using the autosomal positions of the 1240K dataset.

Data S7-S11. (separate file)
qpAdm modelling results.
Combined as tabs in Microsoft Excel spreadsheet:
Data S7. "qpAdm_distal_all"
All distal qpAdm models tested using the autosomal positions of the 1240K dataset.
Data S8. "qpAdm_distal_chosen1"
Distal qpAdm models of admixture between Ukraine_Trypillia, Ukraine_Yamnaya and Mongolia_EIA_SlabGrave_1, tested using the autosomal positions of the 1240K dataset.
Data S9. "qpAdm_distal_chosen2"
Distal qpAdm models of admixture between Central_MN, Ukraine_Yamnaya and Mongolia_EIA_SlabGrave_1, tested using the autosomal positions of the 1240K dataset.
Data S10. "qpAdm_distal_MAXp"
Distal qpAdm models of admixture between a European early farmer group, a Yamnaya group and a Mongolian group resulting in the highest p value for each target group, tested using the autosomal positions of the 1240K dataset.
Data S11. "qpAdm_proximal"
Proximal qpAdm models tested using the autosomal positions of the 1240K dataset.

Data S12-S13. (separate file)
f3 statistics' results.
Combined as tabs in Microsoft Excel spreadsheet:
Data S12. "f3_autosomal"
Outgroup f3 statistics' results of comparisons with ancient populations using the autosomal positions of the 1240K dataset.
Data S13. "f3_X"
Outgroup f3 statistics' results of comparisons with ancient populations using the X chromosome positions of the 1240K dataset.

Data S14. (separate file)
Kinship analysis results of Scythians.
Kinship analysis output of Scythians from KIN software.

REFERENCES AND NOTES

1. M. E. Allentoft, M. Sikora, K.-G. Sjögren, S. Rasmussen, M. Rasmussen, J. Stenderup, P. B. Damgaard, H. Schroeder, T. Ahlström, L. Vinner, A.-S. Malaspinas, A. Margaryan, T. Higham, D. Chivall, N. Lynnerup, L. Harvig, J. Baron, P. Della Casa, P. Dąbrowski, P. R. Duffy, A. V. Ebel, A. Epimakhov, K. Frei, M. Furmanek, T. Gralak, A. Gromov, S. Gronkiewicz, G. Grupe, T. Hajdu, R. Jarysz, V. Khartanovich, A. Khokhlov, V. Kiss, J. Kolář, A. Kriiska, I. Lasak, C. Longhi, G. McGlynn, A. Merkevicius, I. Merkyte, M. Metspalu, R. Mkrtchyan, V. Moiseyev, L. Paja, G. Pálfi, D. Pokutta, Ł. Pospieszny, T. D. Price, L. Saag, M. Sablin, N. Shishlina, V. Smrčka, V. I. Soenov, V. Szeverényi, G. Tóth, S. V. Trifanova, L. Varul, M. Vicze, L. Yepiskoposyan, V. Zhitenev, L. Orlando, T. Sicheritz-Pontén, S. Brunak, R. Nielsen, K. Kristiansen, E. Willerslev, Population genomics of Bronze Age Eurasia. *Nature* **522**, 167–172 (2015).

2. W. Haak, I. Lazaridis, N. Patterson, N. Rohland, S. Mallick, B. Llamas, G. Brandt, S. Nordenfelt, E. Harney, K. Stewardson, Q. Fu, A. Mittnik, E. Bánffy, C. Economou, M. Francken, S. Friederich, R. G. Pena, F. Hallgren, V. Khartanovich, A. Khokhlov, M. Kunst, P. Kuznetsov, H. Meller, O. Mochalov, V. Moiseyev, N. Nicklisch, S. L. Pichler, R. Risch, M. A. Rojo Guerra, C. Roth, A. Szécsényi-Nagy, J. Wahl, M. Meyer, J. Krause, D. Brown, D. Anthony, A. Cooper, K. W. Alt, D. Reich, Massive migration from the steppe was a source for Indo-European languages in Europe. *Nature* **522**, 207–211 (2015).

3. J. Fort, Demic and cultural diffusion propagated the Neolithic transition across different regions of Europe. *J. R. Soc. Interface* **12**, 20150166 (2015).

4. B. Preda-Bălănică, A. Frînculeasa, V. Heyd, The Yamnaya Impact North of the Lower Danube: A tale of newcomers and locals. *Bulletin de la Société préhistorique française* **117**, 85–101 (2020).

5. G. M. Matuzevičiūtė, S. Telizhenko, The first farmers of Ukraine: An archaeobotanical investigation and AMS dating of wheat grains from the Ratniv-2 site. *Archaeologia Lituana* **17**, 100–111 (2017).

6. I. Mathieson, S. Alpaslan-Roodenberg, C. Posth, A. Szécsényi-Nagy, N. Rohland, S. Mallick, I. Olalde, N. Broomandkhoshbacht, F. Candilio, O. Cheronet, D. Fernandes, M. Ferry, B. Gamarra, G. G. Fortes, W. Haak, E. Harney, E. Jones, D. Keating, B. Krause-Kyora, I. Kucukkalipci, M. Michel, A. Mittnik, K. Nägele, M. Novak, J. Oppenheimer, N. Patterson, S. Pfrengle, K. Sirak, K. Stewardson, S. Vai, S. Alexandrov, K. W. Alt, R. Andreescu, D. Antonović, A. Ash, N. Atanassova, K. Bacvarov, M. B. Gusztáv, H. Bocherens, M. Bolus, A. Boroneanţ, Y. Boyadzhiev, A. Budnik, J. Burmaz, S. Chohadzhiev, N. J. Conard, R. Cottiaux, M. Čuka, C. Cupillard, D. G. Drucker, N. Elenski, M. Francken, B. Galabova, G. Ganetsovski, B. Gély, T. Hajdu, V. Handzhyiska, K. Harvati, T. Higham, S. Iliev, I. Janković, I. Karavanić, D. J. Kennett, D. Komšo, A. Kozak, D. Labuda, M. Lari, C. Lazar, M. Leppek, K. Leshtakov, D. L. Vetro, D. Los, I. Lozanov, M. Malina, F. Martini, K. McSweeney, H. Meller, M. Menđušić, P. Mirea, V. Moiseyev, V. Petrova, T. D. Price, A. Simalcsik, L. Sineo, M. Šlaus, V. Slavchev, P. Stanev, A. Starović, T. Szeniczey, S. Talamo, M. Teschler-Nicola, C. Thevenet, I. Valchev, F. Valentin, S. Vasilyev, F. Veljanovska, S. Venelinova, E. Veselovskaya, B. Viola, C. Virag, J. Zaninović, S. Zäuner, P. W. Stockhammer, G. Catalano, R. Krauß, D. Caramelli, G. Zariņa, B. Gaydarska, M. Lillie, A. G. Nikitin, I. Potekhina, A. Papathanasiou, D. Borić, C. Bonsall, J. Krause, R. Pinhasi, D. Reich, The genomic history of southeastern Europe. *Nature* **555**, 197–203 (2018).

7. M. Järve, L. Saag, C. L. Scheib, A. K. Pathak, F. Montinaro, L. Pagani, R. Flores, M. Guellil, L. Saag, K. Tambets, A. Kushniarevich, A. Solnik, L. Varul, S. Zadnikov, O. Petrauskas, M. Avramenko, B. Magomedov, S. Didenko, G. Toshev, I. Bruyako, D. Grechko, V. Okatenko, K. Gorbenko, O. Smyrnov, A. Heiko, R. Reida, S. Sapiehin, S. Sirotin, A. Tairov, A. Beisenov, M. Starodubtsev, V. Vasilev, A. Nechvaloda, B. Atabiev, S. Litvinov, N. Ekomasova, M. Dzhaubermezov, S. Voroniatov, O. Utevska, I. Shramko, E. Khusnutdinova, M. Metspalu, N. Savelev, A. Kriiska, T. Kivisild, R. Villems, Shifts in the genetic landscape of the Western Eurasian steppe associated with the beginning and end of the Scythian dominance. *Curr. Biol.* **29**, 2430–2441.e10 (2019).

8. I. Mathieson, I. Lazaridis, N. Rohland, S. Mallick, N. Patterson, S. A. Roodenberg, E. Harney, K. Stewardson, D. Fernandes, M. Novak, K. Sirak, C. Gamba, E. R. Jones, B. Llamas, S. Dryomov, J. Pickrell, J. L. Arsuaga, J. M. B. de Castro, E. Carbonell, F. Gerritsen, A. Khokhlov, P. Kuznetsov, M. Lozano, H. Meller, O. Mochalov, V. Moiseyev, M. A. R. Guerra,

J. Roodenberg, J. M. Vergès, J. Krause, A. Cooper, K. W. Alt, D. Brown, D. Anthony, C. Lalueza-Fox, W. Haak, R. Pinhasi, D. Reich, Genome-wide patterns of selection in 230 ancient Eurasians. *Nature* **528**, 499–503 (2015).

9. O. Subtelny, *Ukraine: A History, Fourth Edition* (University of Toronto Press, 2009).

10. P. P. Tolochko, Ed., *Давня історія України. Том 1* [Ancient History of Ukraine. Volume 1] (Naukova Dumka, Kyiv, 1997).

11. P. P. Tolochko, Ed., *Давня історія України. Том 2* [Ancient History of Ukraine. Volume 2] (Naukova Dumka, Kyiv, 1998).

12. P. P. Tolochko, D. N. Kozak, O. P. Motsya, V. Yu. Murzin, V. V. Otroshchenko, S. P. Segeda, *Етнічна історія давньої України* [Ethnic History of Ancient Ukraine] (Institute of Archeology of the National Academy of Sciences of Ukraine, Kyiv, 2000).

13. S. V. Makhortyh, *Киммерийцы Северного Причерноморья* [Cimmerians of the Northern Black Sea] (The Way, Kyiv, 2005).

14. S. A. Skory, *Скифы в днепровской правобережной лесостепи (проблема выделения иранского этнокультурного элемента)* [Scythians in the Dnieper Right-bank Forest-steppe (The Problem of Identifying the Iranian Ethnocultural Element] (Institute of Archaeology of the National Academy of Sciences of Ukraine, Kyiv, 2003).

15. M. Unterländer, F. Palstra, I. Lazaridis, A. Pilipenko, Z. Hofmanová, M. Groß, C. Sell, J. Blöcher, K. Kirsanow, N. Rohland, B. Rieger, E. Kaiser, W. Schier, D. Pozdniakov, A. Khokhlov, M. Georges, S. Wilde, A. Powell, E. Heyer, M. Currat, D. Reich, Z. Samashev, H. Parzinger, V. I. Molodin, J. Burger, Ancestry and demography and descendants of Iron Age nomads of the Eurasian Steppe. *Nat. Commun.* **8**, 14615 (2017).

16. M. Krzewińska, G. M. Kılınç, A. Juras, D. Koptekin, M. Chyleński, A. G. Nikitin, N. Shcherbakov, I. Shuteleva, T. Leonova, L. Kraeva, F. A. Sungatov, A. N. Sultanova, I. Potekhina, S. Łukasik, M. Krenz-Niedbała, L. Dalén, V. Sinika, M. Jakobsson, J. Storå, A.

Götherström, Ancient genomes suggest the eastern Pontic-Caspian steppe as the source of western Iron Age nomads. *Sci. Adv.* **4**, eaat4457 (2018).

17. P. de Barros Damgaard, N. Marchi, S. Rasmussen, M. Peyrot, G. Renaud, T. Korneliussen, J. V. Moreno-Mayar, M. W. Pedersen, A. Goldberg, E. Usmanova, N. Baimukhanov, V. Loman, L. Hedeager, A. G. Pedersen, K. Nielsen, G. Afanasiev, K. Akmatov, A. Aldashev, A. Alpaslan, G. Baimbetov, V. I. Bazaliiskii, A. Beisenov, B. Boldbaatar, B. Boldgiv, C. Dorzhu, S. Ellingvag, D. Erdenebaatar, R. Dajani, E. Dmitriev, V. Evdokimov, K. M. Frei, A. Gromov, A. Goryachev, H. Hakonarson, T. Hegay, Z. Khachatryan, R. Khaskhanov, E. Kitov, A. Kolbina, T. Kubatbek, A. Kukushkin, I. Kukushkin, N. Lau, A. Margaryan, I. Merkyte, I. V. Mertz, V. K. Mertz, E. Mijiddorj, V. Moiyesev, G. Mukhtarova, B. Nurmukhanbetov, Z. Orozbekova, I. Panyushkina, K. Pieta, V. Smrčka, I. Shevnina, A. Logvin, K.-G. Sjögren, T. Štolcová, K. Tashbaeva, A. Tkachev, T. Tulegenov, D. Voyakin, L. Yepiskoposyan, S. Undrakhbold, V. Varfolomeev, A. Weber, N. Kradin, M. E. Allentoft, L. Orlando, R. Nielsen, M. Sikora, E. Heyer, K. Kristiansen, E. Willerslev, 137 ancient human genomes from across the Eurasian steppes. *Nature* **557**, 369–374 (2018).

18. G. A. Gnecchi-Ruscone, E. Khussainova, N. Kahbatkyzy, L. Musralina, M. A. Spyrou, R. A. Bianco, R. Radzeviciute, N. F. G. Martins, C. Freund, O. Iksan, A. Garshin, Z. Zhaniyazov, B. Bekmanov, E. Kitov, Z. Samashev, A. Beisenov, N. Berezina, Y. Berezin, A. Z. Bíró, S. Évinger, A. Bissembaev, G. Akhatov, A. Mamedov, A. Onggaruly, D. Voyakin, A. Chotbayev, Y. Kariyev, A. Buzhilova, L. Djansugurova, C. Jeong, J. Krause, Ancient genomic time transect from the Central Asian Steppe unravels the history of the Scythians. *Sci. Adv.* **7**, eabe4414 (2021).

19. G. R. Tsetskhladze, *The Greek Colonisation of the Black Sea Area: Historical Interpretation of Archaeology* (Franz Steiner Verlag, 1998).

20. V. V. Lapin, *Греческая колонизация Северного Причерноморья* [Greek Colonization of the Northern Black Sea Region] (Naukova Dumka, Kyiv, 1966).

21. S. D. Lysenko, Тшинецкий культурный круг—связующее звено между балтийским и черноморским регионами в эпоху поздней бронзы (2 тыс. до н. э.) [The Trzciniec cultural

circle—A link between the Baltic and Black Sea regions in the Late Bronze Age (2nd millennium BC)]. *History Archeology 1*, 23–66 (2017).

22. M. N. Daragan, *Начало раннего железного века в Днепровской правобережной лесостепи* [The Beginning of the Early Iron Age in the Dnipro Right-bank Forest-steppe] (KNT, Kyiv, 2011).

23. M. Bandrivskyi, *Культурно-історичні процеси на Прикарпатті і Західному Поділлі в пізній період епохи бронзи–на початку доби раннього заліза* [Cultural-historical Processes in the East Carpathians and Western Podillya in the Late Bronze Age–At the Beginning of the Early Iron Age] (I. Krypyakevich Institute of Ukrainian Studies, National Academy of Sciences of Ukraine, Lviv, 2014).

24. G. Kazakevich, The La Tène culture of the Trans-Carpathian area: Is the migration model still relevant? *Bulletin of Taras Shevchenko Kyiv National University* (2015).

25. H. Kazakevich, *Кельти на землях України: археологічна, мовна та культурна спадщина* [Celts on the Lands of Ukraine: Archaeological, Linguistic and Cultural Heritage] (Publisher Serhii Nalivayko, Kyiv, 2010).

26. S. P. Pachkova, *Зарубинецкая культура и латенизированные культуры Европы* [Zarubinetskaya Culture and Latenized Cultures of Europe] (Institute of Archeology of the National Academy of Sciences of Ukraine, Kyiv, 2007).

27. V. D. Baran, *Черняхівська культура: за матеріалами Верхнього Дністра і Західного Бугу* [Chernyakhiv Culture: Based on the Materials of Upper Dniester and Western Bug] (Naukova Dumka, Kyiv, 1981).

28. B. V. Magomedov, Черняховская культура. Проблема этноса [Chernyakhiv Culture. The Problem of Ethnos] (Monumenta Studia Gothica, UMCS, Lublin, 2001).

29. L. L. Zaliznyak, O. P. Motsya, V. M. Zubar, K. P. Bunyatyan, V. V. Otroshchenko, R. V. Terpylovskyi, *Археологія України* [Archeology of Ukraine] (Lybid, Kyiv, 2005).

30. N. S. Abashina, D. N. Kozak, E. V. Sinytsia, R. V. Terpylovskyi, *Давні слов'яни. Археологія та історія* [Ancient Slavs. Archeology and History] (Ancient World, Kyiv, 2012).

31. P. P. Tolochko, Ed., *Давня історія України. Том 3* [Ancient History of Ukraine] (Naukova Dumka, Kyiv, 2000), vol. 3.

32. A. G. Toshchev, "Новые археологические сведения по истории степных кочевников на юге Украины" ["New archaeological data on the history of steppe nomads in the south of Ukraine"] in *Древнее Причерноморье 10* [Ancient Black Sea Region 10] (A. S. Fridman, Odesa, 2013), pp. 583–586.

33. E. R. Jones, G. Zarina, V. Moiseyev, E. Lightfoot, P. R. Nigst, A. Manica, R. Pinhasi, D. G. Bradley, The neolithic transition in the Baltic was not driven by admixture with early european farmers. *Curr. Biol.* **27**, 576–582 (2017).

34. A. Margaryan, D. J. Lawson, M. Sikora, F. Racimo, S. Rasmussen, I. Moltke, L. M. Cassidy, E. Jørsboe, A. Ingason, M. W. Pedersen, T. Korneliussen, H. Wilhelmson, M. M. Buś, P. de Barros Damgaard, R. Martiniano, G. Renaud, C. Bhérer, J. V. Moreno-Mayar, A. K. Fotakis, M. Allen, R. Allmäe, M. Molak, E. Cappellini, G. Scorrano, H. McColl, A. Buzhilova, A. Fox, A. Albrechtsen, B. Schütz, B. Skar, C. Arcini, C. Falys, C. H. Jonson, D. Błaszczyk, D. Pezhemsky, G. Turner-Walker, H. Gestsdóttir, I. Lundstrøm, I. Gustin, I. Mainland, I. Potekhina, I. M. Muntoni, J. Cheng, J. Stenderup, J. Ma, J. Gibson, J. Peets, J. Gustafsson, K. H. Iversen, L. Simpson, L. Strand, L. Loe, M. Sikora, M. Florek, M. Vretemark, M. Redknap, M. Bajka, T. Pushkina, M. Søvsø, N. Grigoreva, T. Christensen, O. Kastholm, O. Uldum, P. Favia, P. Holck, S. Sten, S. V. Arge, S. Ellingvåg, V. Moiseyev, W. Bogdanowicz, Y. Magnusson, L. Orlando, P. Pentz, M. D. Jessen, A. Pedersen, M. Collard, D. G. Bradley, M. L. Jørkov, J. Arneborg, N. Lynnerup, N. Price, M. T. P. Gilbert, M. E. Allentoft, J. Bill, S. M. Sindbæk, L. Hedeager, K. Kristiansen, R. Nielsen, T. Werge, E. Willerslev, Population genomics of the Viking world. *Nature* **585**, 390–396 (2020).

35. P. Gelabert, R. W. Schmidt, D. M. Fernandes, J. K. Karsten, T. K. Harper, G. D. Madden, S. H. Ledogar, M. Sokhatsky, H. Oota, D. J. Kennett, R. Pinhasi, Genomes from Verteba cave suggest diversity within the Trypillians in Ukraine. *Sci. Rep.* **12**, 7242 (2022).

36. T. M. Mattila, E. M. Svensson, A. Juras, T. Günther, N. Kashuba, T. Ala-Hulkko, M. Chyleński, J. McKenna, Ł. Pospieszny, M. Constantinescu, M. Rotea, N. Palincaş, S. Wilk, L. Czerniak, J. Kruk, J. Łapo, P. Makarowicz, I. Potekhina, A. Soficaru, M. Szmyt, K. Szostek, A. Götherström, J. Storå, M. G. Netea, A. G. Nikitin, P. Persson, H. Malmström, M. Jakobsson, Genetic continuity, isolation, and gene flow in Stone Age Central and Eastern Europe. *Commun Biol* **6**, 793 (2023).

37. A. G. Nikitin, M. Videiko, N. Patterson, V. Renson, D. Reich, Interactions between Trypillian farmers and North Pontic forager-pastoralists in Eneolithic central Ukraine. *PLOS ONE* **18**, e0285449 (2023).

38. S. Penske, A. B. Rohrlach, A. Childebayeva, G. Gnecchi-Ruscone, C. Schmid, M. A. Spyrou, G. U. Neumann, N. Atanassova, K. Beutler, K. Boyadzhiev, Y. Boyadzhiev, I. Bruyako, A. Chohadzhiev, B. Govedarica, M. Karaucak, R. Krauss, M. Leppek, I. Manzura, K. Privat, S. Ross, V. Slavchev, A. Sobotkova, M. Toderaş, T. Valchev, H. Ringbauer, P. W. Stockhammer, S. Hansen, J. Krause, W. Haak, Early contact between late farming and pastoralist societies in southeastern Europe. *Nature* **620**, 358–365 (2023).

39. M. Chyleński, P. Makarowicz, A. Juras, M. Krzewińska, Ł. Pospieszny, E. Ehler, A. Breszka, J. Górski, H. Taras, A. Szczepanek, M. Polańska, P. Włodarczak, A. Lasota-Kuś, I. Wójcik, J. Romaniszyn, M. Szmyt, A. Kośko, M. Ignaczak, S. Sadowski, A. Matoga, A. Grossman, V. Ilchyshyn, M. O. Yahodinska, A. Romańska, K. Tunia, M. Przybyła, R. Grygiel, K. Szostek, M. Dabert, A. Götherström, M. Jakobsson, H. Malmström, Patrilocality and hunter-gatherer-related ancestry of populations in East-Central Europe during the middle bronze age. *Nat. Commun.* **14**, 4395 (2023).

40. M. E. Allentoft, M. Sikora, A. Refoyo-Martínez, E. K. Irving-Pease, A. Fischer, W. Barrie, A. Ingason, J. Stenderup, K.-G. Sjögren, A. Pearson, B. Sousa da Mota, B. Schulz Paulsson, A. Halgren, R. Macleod, M. L. S. Jørkov, F. Demeter, L. Sørensen, P. O. Nielsen, R. A. Henriksen, T. Vimala, H. McColl, A. Margaryan, M. Ilardo, A. Vaughn, M. Fischer Mortensen, A. B. Nielsen, M. Ulfeldt Hede, N. N. Johannsen, P. Rasmussen, L. Vinner, G. Renaud, A. Stern, T. Z. T. Jensen, G. Scorrano, H. Schroeder, P. Lysdahl, A. D. Ramsøe, A. Skorobogatov, A. J. Schork, A. Rosengren, A. Ruter, A. Outram, A. A. Timoshenko, A.

Buzhilova, A. Coppa, A. Zubova, A. M. Silva, A. J. Hansen, A. Gromov, A. Logvin, A. B. Gotfredsen, B. Henning Nielsen, B. González-Rabanal, C. Lalueza-Fox, C. J. McKenzie, C. Gaunitz, C. Blasco, C. Liesau, C. Martinez-Labarga, D. V. Pozdnyakov, D. Cuenca-Solana, D. O. Lordkipanidze, D. En'shin, D. C. Salazar-García, T. D. Price, D. Borić, E. Kostyleva, E. V. Veselovskaya, E. R. Usmanova, E. Cappellini, E. Brinch Petersen, E. Kannegaard, F. Radina, F. Eylem Yediay, H. Duday, I. Gutiérrez-Zugasti, I. Merts, I. Potekhina, I. Shevnina, I. Altinkaya, J. Guilaine, J. Hansen, J. E. Aura Tortosa, J. Zilhão, J. Vega, K. Buck Pedersen, K. Tunia, L. Zhao, L. N. Mylnikova, L. Larsson, L. Metz, L. Yepiskoposyan, L. Pedersen, L. Sarti, L. Orlando, L. Slimak, L. Klassen, M. Blank, M. González-Morales, M. Silvestrini, M. Vretemark, M. S. Nesterova, M. Rykun, M. F. Rolfo, M. Szmyt, M. Przybyła, M. Calattini, M. Sablin, M. Dobisíková, M. Meldgaard, M. Johansen, N. Berezina, N. Card, N. A. Saveliev, O. Poshekhonova, O. Rickards, O. V. Lozovskaya, O. Gábor, O. C. Uldum, P. Aurino, P. Kosintsev, P. Courtaud, P. Ríos, P. Mortensen, P. Lotz, P. Persson, P. Bangsgaard, P. de Barros Damgaard, P. Vang Petersen, P. P. Martinez, P. Włodarczak, R. V. Smolyaninov, R. Maring, R. Menduiña, R. Badalyan, R. Iversen, R. Turin, S. Vasilyev, S. Wåhlin, S. Borutskaya, S. Skochina, S. A. Sørensen, S. H. Andersen, T. Jørgensen, Y. B. Serikov, V. I. Molodin, V. Smrcka, V. Merts, V. Appadurai, V. Moiseyev, Y. Magnusson, K. H. Kjær, N. Lynnerup, D. J. Lawson, P. H. Sudmant, S. Rasmussen, T. S. Korneliussen, R. Durbin, R. Nielsen, O. Delaneau, T. Werge, F. Racimo, K. Kristiansen, E. Willerslev, Population genomics of post-glacial western Eurasia. *Nature* **625**, 301–311 (2024).

41. M. Furholt, De-contaminating the aDNA – archaeology dialogue on mobility and migration: Discussing the culture-historical legacy. *Curr. Swed. Archaeol.* **27**, 53–68 (2019).

42. S. Mallick, A. Micco, M. Mah, H. Ringbauer, I. Lazaridis, I. Olalde, N. Patterson, D. Reich, The Allen Ancient DNA Resource (AADR) a curated compendium of ancient human genomes. *Sci. Data* **11**, 182 (2024).

43. V. Grugni, A. Raveane, L. Ongaro, V. Battaglia, B. Trombetta, G. Colombo, M. R. Capodiferro, A. Olivieri, A. Achilli, U. A. Perego, J. Motta, M. Tribaldos, S. R. Woodward, L. Ferretti, F. Cruciani, A. Torroni, O. Semino, Analysis of the human Y-chromosome haplogroup Q characterizes ancient population movements in Eurasia and the Americas. *BMC Biol.* **17**, 3 (2019).

44. A.-M. Ilumäe, H. Post, R. Flores, M. Karmin, H. Sahakyan, M. Mondal, F. Montinaro, L. Saag, C. Bormans, L. F. Sanchez, A. Ameur, U. Gyllensten, M. Kals, R. Mägi, L. Pagani, D. M. Behar, S. Rootsi, R. Villems, Phylogenetic history of patrilineages rare in northern and eastern Europe from large-scale re-sequencing of human Y-chromosomes. *Eur. J. Hum. Genet.* **29**, 1510–1519 (2021).

45. Q. Fu, C. Posth, M. Hajdinjak, M. Petr, S. Mallick, D. Fernandes, A. Furtwängler, W. Haak, M. Meyer, A. Mittnik, B. Nickel, A. Peltzer, N. Rohland, V. Slon, S. Talamo, I. Lazaridis, M. Lipson, I. Mathieson, S. Schiffels, P. Skoglund, A. P. Derevianko, N. Drozdov, V. Slavinsky, A. Tsybankov, R. G. Cremonesi, F. Mallegni, B. Gély, E. Vacca, M. R. G. Morales, L. G. Straus, C. Neugebauer-Maresch, M. Teschler-Nicola, S. Constantin, O. T. Moldovan, S. Benazzi, M. Peresani, D. Coppola, M. Lari, S. Ricci, A. Ronchitelli, F. Valentin, C. Thevenet, K. Wehrberger, D. Grigorescu, H. Rougier, I. Crevecoeur, D. Flas, P. Semal, M. A. Mannino, C. Cupillard, H. Bocherens, N. J. Conard, K. Harvati, V. Moiseyev, D. G. Drucker, J. Svoboda, M. P. Richards, D. Caramelli, R. Pinhasi, J. Kelso, N. Patterson, J. Krause, S. Pääbo, D. Reich, The genetic history of Ice Age Europe. *Nature* **534**, 200–205 (2016).

46. C. Gamba, E. R. Jones, M. D. Teasdale, R. L. McLaughlin, G. Gonzalez-Fortes, V. Mattiangeli, L. Domboróczki, I. Kővári, I. Pap, A. Anders, A. Whittle, J. Dani, P. Raczky, T. F. G. Higham, M. Hofreiter, D. G. Bradley, R. Pinhasi, Genome flux and stasis in a five millennium transect of European prehistory. *Nat. Commun.* **5**, 5257 (2014).

47. M. Rivollat, C. Jeong, S. Schiffels, İ. Küçükkalıpçı, M.-H. Pemonge, A. B. Rohrlach, K. W. Alt, D. Binder, S. Friederich, E. Ghesquière, D. Gronenborn, L. Laporte, P. Lefranc, H. Meller, H. Réveillas, E. Rosenstock, S. Rottier, C. Scarre, L. Soler, J. Wahl, J. Krause, M.-F. Deguilloux, W. Haak, Ancient genome-wide DNA from France highlights the complexity of interactions between Mesolithic hunter-gatherers and Neolithic farmers. *Sci. Adv.* **6**, eaaz5344 (2020).

48. B. Navarro-López, E. Granizo-Rodríguez, L. Palencia-Madrid, C. Raffone, M. Baeta, M. M. de Pancorbo, Phylogeographic review of Y chromosome haplogroups in Europe. *Int. J. Legal Med.* **135**, 1675–1684 (2021).

49. N. Patterson, M. Isakov, T. Booth, L. Büster, C.-E. Fischer, I. Olalde, H. Ringbauer, A. Akbari, O. Cheronet, M. Bleasdale, N. Adamski, E. Altena, R. Bernardos, S. Brace, N. Broomandkhoshbacht, K. Callan, F. Candilio, B. Culleton, E. Curtis, L. Demetz, K. S. D. Carlson, C. J. Edwards, D. M. Fernandes, M. G. B. Foody, S. Freilich, H. Goodchild, A. Kearns, A. M. Lawson, I. Lazaridis, M. Mah, S. Mallick, K. Mandl, A. Micco, M. Michel, G. B. Morante, J. Oppenheimer, K. T. Özdoğan, L. Qiu, C. Schattke, K. Stewardson, J. N. Workman, F. Zalzala, Z. Zhang, B. Agustí, T. Allen, K. Almássy, L. Amkreutz, A. Ash, C. Baillif-Ducros, A. Barclay, L. Bartosiewicz, K. Baxter, Z. Bernert, J. Blažek, M. Bodružić, P. Boissinot, C. Bonsall, P. Bradley, M. Brittain, A. Brookes, F. Brown, L. Brown, R. Brunning, C. Budd, J. Burmaz, S. Canet, S. Carnicero-Cáceres, M. Čaušević-Bully, A. Chamberlain, S. Chauvin, S. Clough, N. Čondić, A. Coppa, O. Craig, M. Črešnar, V. Cummings, S. Czifra, A. Danielisová, R. Daniels, A. Davies, P. de Jersey, J. Deacon, C. Deminger, P. W. Ditchfield, M. Dizdar, M. Dobeš, M. Dobisíková, L. Domboróczki, G. Drinkall, A. Đukić, M. Ernée, C. Evans, J. Evans, M. Fernández-Götz, S. Filipović, A. Fitzpatrick, H. Fokkens, C. Fowler, A. Fox, Z. Gallina, M. Gamble, M. R. González Morales, B. González-Rabanal, A. Green, K. Gyenesei, D. Habermehl, T. Hajdu, D. Hamilton, J. Harris, C. Hayden, J. Hendriks, B. Hernu, G. Hey, M. Horňák, G. Ilon, E. Istvánovits, A. M. Jones, M. B. Kavur, K. Kazek, R. A. Kenyon, A. Khreisheh, V. Kiss, J. Kleijne, M. Knight, L. M. Kootker, P. F. Kovács, A. Kozubová, G. Kulcsár, V. Kulcsár, C. Le Pennec, M. Legge, M. Leivers, L. Loe, O. López-Costas, T. Lord, D. Los, J. Lyall, A. B. Marín-Arroyo, P. Mason, D. Matošević, A. Maxted, L. McIntyre, J. McKinley, K. McSweeney, B. Meijlink, B. G. Mende, M. Menđušić, M. Metlička, S. Meyer, K. Mihovilić, L. Milasinovic, S. Minnitt, J. Moore, G. Morley, G. Mullan, M. Musilová, B. Neil, R. Nicholls, M. Novak, M. Pala, M. Papworth, C. Paresys, R. Patten, D. Perkić, K. Pesti, A. Petit, K. Petriščáková, C. Pichon, C. Pickard, Z. Pilling, T. D. Price, S. Radović, R. Redfern, B. Resutík, D. T. Rhodes, M. B. Richards, A. Roberts, J. Roefstra, P. Sankot, A. Šefčáková, A. Sheridan, S. Skae, M. Šmolíková, K. Somogyi, Á. Somogyvári, M. Stephens, G. Szabó, A. Szécsényi-Nagy, T. Szeniczey, J. Tabor, K. Tankó, C. T. Maria, R. Terry, B. Teržan, M. Teschler-Nicola, J. F. Torres-Martínez, J. Trapp, R. Turle, F. Ujvári, M. van der Heiden, P. Veleminsky, B. Veselka, Z. Vytlačil, C. Waddington, P. Ware, P. Wilkinson, L. Wilson, R. Wiseman, E. Young, J. Zaninović, A. Žitňan, C. Lalueza-Fox, P. de Knijff, I. Barnes, P. Halkon, M. G. Thomas, D. J. Kennett, B. Cunliffe, M. Lillie, N. Rohland,

R. Pinhasi, I. Armit, D. Reich, Large-scale migration into Britain during the Middle to Late Bronze Age. *Nature* **601**, 588–594 (2022).

50. C.-C. Wang, H.-Y. Yeh, A. N. Popov, H.-Q. Zhang, H. Matsumura, K. Sirak, O. Cheronet, A. Kovalev, N. Rohland, A. M. Kim, S. Mallick, R. Bernardos, D. Tumen, J. Zhao, Y.-C. Liu, J.-Y. Liu, M. Mah, K. Wang, Z. Zhang, N. Adamski, N. Broomandkhoshbacht, K. Callan, F. Candilio, K. S. D. Carlson, B. J. Culleton, L. Eccles, S. Freilich, D. Keating, A. M. Lawson, K. Mandl, M. Michel, J. Oppenheimer, K. T. Özdoğan, K. Stewardson, S. Wen, S. Yan, F. Zalzala, R. Chuang, C.-J. Huang, H. Looh, C.-C. Shiung, Y. G. Nikitin, A. V. Tabarev, A. A. Tishkin, S. Lin, Z.-Y. Sun, X.-M. Wu, T.-L. Yang, X. Hu, L. Chen, H. Du, J. Bayarsaikhan, E. Mijiddorj, D. Erdenebaatar, T.-O. Iderkhangai, E. Myagmar, H. Kanzawa-Kiriyama, M. Nishino, K.-I. Shinoda, O. A. Shubina, J. Guo, W. Cai, Q. Deng, L. Kang, D. Li, D. Li, R. Lin, R. Shrestha Nini, L.-X. Wang, L. Wei, G. Xie, H. Yao, M. Zhang, G. He, X. Yang, R. Hu, M. Robbeets, S. Schiffels, D. J. Kennett, L. Jin, H. Li, J. Krause, R. Pinhasi, D. Reich, Genomic insights into the formation of human populations in East Asia. *Nature* **591**, 413–419 (2021).

51. H. Schroeder, A. Margaryan, M. Szmyt, B. Theulot, P. Włodarczak, S. Rasmussen, S. Gopalakrishnan, A. Szczepanek, T. Konopka, T. Z. T. Jensen, B. Witkowska, S. Wilk, M. M. Przybyła, Ł. Pospieszny, K.-G. Sjögren, Z. Belka, J. Olsen, K. Kristiansen, E. Willerslev, K. M. Frei, M. Sikora, N. N. Johannsen, M. E. Allentoft, Unraveling ancestry, kinship, and violence in a Late Neolithic mass grave. *Proc. Natl. Acad. Sci. U.S.A* **116**, 10705–10710 (2019).

52. L. Saag, L. Varul, C. L. Scheib, J. Stenderup, M. E. Allentoft, L. Saag, L. Pagani, M. Reidla, K. Tambets, E. Metspalu, A. Kriiska, E. Willerslev, T. Kivisild, M. Metspalu, Extensive farming in Estonia started through a sex-biased migration from the Steppe. *Curr. Biol.* **27**, 2185–2193.e6 (2017).

53. A. Mittnik, K. Massy, C. Knipper, F. Wittenborn, R. Friedrich, S. Pfrengle, M. Burri, N. Carlichi-Witjes, H. Deeg, A. Furtwängler, M. Harbeck, K. von Heyking, C. Kociumaka, I. Kucukkalipci, S. Lindauer, S. Metz, A. Staskiewicz, A. Thiel, J. Wahl, W. Haak, E. Pernicka,

S. Schiffels, P. W. Stockhammer, J. Krause, Kinship-based social inequality in Bronze Age Europe. *Science* **366**, 731–734 (2019).

54. A. Linderholm, G. M. Kılınç, A. Szczepanek, P. Włodarczak, P. Jarosz, Z. Belka, J. Dopieralska, K. Werens, J. Górski, M. Mazurek, M. Hozer, M. Rybicka, M. Ostrowski, J. Bagińska, W. Koman, R. Rodríguez-Varela, J. Storå, A. Götherström, M. Krzewińska, Corded Ware cultural complexity uncovered using genomic and isotopic analysis from south-eastern Poland. *Sci. Rep.* **10**, 6885 (2020).

55. L. Saag, S. V. Vasilyev, L. Varul, N. V. Kosorukova, D. V. Gerasimov, S. V. Oshibkina, S. J. Griffith, A. Solnik, L. Saag, E. D'Atanasio, E. Metspalu, M. Reidla, S. Rootsi, T. Kivisild, C. L. Scheib, K. Tambets, A. Kriiska, M. Metspalu, Genetic ancestry changes in Stone to Bronze Age transition in the East European plain. *Sci Adv* **7**, eabd6535 (2021).

56. M. Derenko, B. Malyarchuk, T. Grzybowski, G. Denisova, U. Rogalla, M. Perkova, I. Dambueva, I. Zakharov, Origin and post-glacial dispersal of mitochondrial DNA haplogroups C and D in northern Asia. *PLOS ONE* **5**, e15214 (2010).

57. J. M. Monroy Kuhn, M. Jakobsson, T. Günther, Estimating genetic kin relationships in prehistoric populations. *PLOS ONE* **13**, e0195491 (2018).

58. D. Popli, S. Peyrégne, B. M. Peter, KIN: A method to infer relatedness from low-coverage ancient DNA. *Genome Biol.* **24**, 10 (2023).

59. M. A. Dzhaubermezov, N. V. Ekomasova, M. Reidla, S. S. Litvinov, L. R. Gabidullina, R. Villems, E. K. Khusnutdinova, Genetic characterization of balkars and karachays Using mtDNA Data. *Russ. J. Genet.* **55**, 114–123 (2019).

60. B. Malyarchuk, T. Grzybowski, M. Derenko, M. Perkova, T. Vanecek, J. Lazur, P. Gomolcak, I. Tsybovsky, Mitochondrial DNA phylogeny in Eastern and Western Slavs. *Mol. Biol. Evol.* **25**, 1651–1658 (2008).

61. T. Kivisild, H.-V. Tolk, J. Parik, Y. Wang, S. S. Papiha, H.-J. Bandelt, R. Villems, The emerging limbs and twigs of the East Asian mtDNA tree. *Mol. Biol. Evol.* **19**, 1737–1751 (2002).

62. M. Richards, V. Macaulay, E. Hickey, E. Vega, B. Sykes, V. Guida, C. Rengo, D. Sellitto, F. Cruciani, T. Kivisild, R. Villems, M. Thomas, S. Rychkov, O. Rychkov, Y. Rychkov, M. Gölge, D. Dimitrov, E. Hill, D. Bradley, V. Romano, F. Calì, G. Vona, A. Demaine, S. Papiha, C. Triantaphyllidis, G. Stefanescu, J. Hatina, M. Belledi, A. Di Rienzo, A. Novelletto, A. Oppenheim, S. Nørby, N. Al-Zaheri, S. Santachiara-Benerecetti, R. Scozari, A. Torroni, H. J. Bandelt, Tracing European founder lineages in the Near Eastern mtDNA pool. *Am. J. Hum. Genet.* **67**, 1251–1276 (2000).

63. C. Herrnstadt, J. L. Elson, E. Fahy, G. Preston, D. M. Turnbull, C. Anderson, S. S. Ghosh, J. M. Olefsky, M. F. Beal, R. E. Davis, N. Howell, Reduced-median-network analysis of complete mitochondrial DNA coding-region sequences for the major African, Asian, and European haplogroups. *Am. J. Hum. Genet.* **70**, 1152–1171 (2002).

64. M. Ingman, H. Kaessmann, S. Pääbo, U. Gyllensten, Mitochondrial genome variation and the origin of modern humans. *Nature* **408**, 708–713 (2000).

65. Z. Wang, M. Wang, L. Hu, G. He, S. Nie, Evolutionary profiles and complex admixture landscape in East Asia: New insights from modern and ancient Y chromosome variation perspectives. *Heliyon* **10**, e30067 (2024).

66. H. Sahakyan, A. Margaryan, L. Saag, M. Karmin, R. Flores, M. Haber, A. Kushniarevich, Z. Khachatryan, A. Bahmanimehr, J. Parik, T. Karafet, B. Yunusbayev, T. Reisberg, A. Solnik, E. Metspalu, A. Hovhannisyan, E. K. Khusnutdinova, D. M. Behar, M. Metspalu, L. Yepiskoposyan, S. Rootsi, R. Villems, Origin and diffusion of human Y chromosome haplogroup J1-M267. *Sci. Rep.* **11**, 6659 (2021).

67. N. Patterson, A. L. Price, D. Reich, Population structure and eigenanalysis. *PLoS Genet.* **2**, e190 (2006).

68. A. Goldberg, T. Günther, N. A. Rosenberg, M. Jakobsson, Ancient X chromosomes reveal contrasting sex bias in Neolithic and Bronze Age Eurasian migrations. *Proc. Natl. Acad. Sci. U.S.A.* **114**, 2657–2662 (2017).

69. A. R. Ventresca Miller, J. A. Johnson, S. Makhortykh, L. Litvinova, T. Taylor, R. Rolle, C. A. Makarewicz, Mobility and diet in the Iron Age Pontic forest-steppe: A multi-isotopic study of urban populations at Bel'sk. *Archaeometry* **61**, 1399–1416 (2019).

70. O. V. Petrauskas, Komariv — ein Werkstattzentrum barbarischen Europas aus spätrömischer Zeit (Forschungsgeschichte, einige Ergebnisse und mögliche Perspektiven). Ephemeris Napocensis XXIV, 87–116 (2014).

71. O. V. Petrauskas, M. Avramenko, The settlement Komariv – glass-production centre in the European Barbaricum: A cultural and natural environment. *Plural* **7**, 68–91 (2019).

72. E. M. Stern, Roman glassblowing in a cultural context. *Am. J. Archaeol.* **103**, 441–484 (1999).

73. K. I. Krasilnikov, "Население степного Подонцовья в хазарское время" ["The population of the steppe Podontsov region in the Khazar time"] in *Дивногорский сборник 1* [Divnogorsk Collection 1] (VSU Publishing House, Voronezh, 2009), pp. 52–82.

74. G. E. Afanasyev, *Население лесостепной зоны бассейна Среднего Дона в VIII-X вв.: аланский вариант салтово-маяцкой культуры* [Population of the Forest-steppe Zone of the Middle Don Basin in the 8th-10th Centuries: Alanian Version of the Saltovo-Mayaki Culture] (Archaeological discoveries at new construction sites vol 2, Nauka, Moscow, 1987).

75. P. B. Damgaard, A. Margaryan, H. Schroeder, L. Orlando, E. Willerslev, M. E. Allentoft, Improving access to endogenous DNA in ancient bones and teeth. *Sci. Rep.* **5**, 11184 (2015).

76. N. Rohland, I. Glocke, A. Aximu-Petri, M. Meyer, Extraction of highly degraded DNA from ancient bones, teeth and sediments for high-throughput sequencing. *Nat. Protoc.* **13**, 2447–2461 (2018).

77. M.-T. Gansauge, A. Aximu-Petri, S. Nagel, M. Meyer, Manual and automated preparation of single-stranded DNA libraries for the sequencing of DNA from ancient biological remains and other sources of highly degraded DNA. *Nat. Protoc.* **15**, 2279–2300 (2020).

78. M. Kircher, S. Sawyer, M. Meyer, Double indexing overcomes inaccuracies in multiplex sequencing on the Illumina platform. *Nucleic Acids Res.* **40**, e3 (2012).

79. J. A. Fellows Yates, T. C. Lamnidis, M. Borry, A. Andrades Valtueña, Z. Fagernäs, S. Clayton, M. U. Garcia, J. Neukamm, A. Peltzer, Reproducible, portable, and efficient ancient genome reconstruction with nf-core/eager. *PeerJ* **9**, e10947 (2021).

80. M. Schubert, S. Lindgreen, L. Orlando, AdapterRemoval v2: Rapid adapter trimming, identification, and read merging. *BMC Res. Notes* **9**, 88 (2016).

81. H. Li, R. Durbin, Fast and accurate short read alignment with Burrows-Wheeler transform. *Bioinformatics* **25**, 1754–1760 (2009).

82. M. Poullet, L. Orlando, Assessing DNA sequence alignment methods for characterizing ancient genomes and methylomes. *Front. Ecol. Evol.* **8**, 10.3389/fevo.2020.00105 (2020).

83. A. Peltzer, G. Jäger, A. Herbig, A. Seitz, C. Kniep, J. Krause, K. Nieselt, EAGER: Efficient ancient genome reconstruction. *Genome Biol.* **17**, 60 (2016).

84. H. Li, B. Handsaker, A. Wysoker, T. Fennell, J. Ruan, N. Homer, G. Marth, G. Abecasis, R. Durbin, 1000 Genome Project Data Processing Subgroup, The Sequence Alignment/Map format and SAMtools. *Bioinformatics* **25**, 2078–2079 (2009).

85. J. Neukamm, A. Peltzer, K. Nieselt, DamageProfiler: Fast damage pattern calculation for ancient DNA. *Bioinformatics* **37**, 3652–3653 (2021).

86. G. Renaud, V. Slon, A. T. Duggan, J. Kelso, Schmutzi: Estimation of contamination and endogenous mitochondrial consensus calling for ancient DNA. *Genome Biol.* **16**, 224 (2015).

87. M. Rasmussen, X. Guo, Y. Wang, K. E. Lohmueller, S. Rasmussen, A. Albrechtsen, L. Skotte, S. Lindgreen, M. Metspalu, T. Jombart, T. Kivisild, W. Zhai, A. Eriksson, A. Manica,

L. Orlando, F. M. De La Vega, S. Tridico, E. Metspalu, K. Nielsen, M. C. Ávila-Arcos, J. V. Moreno-Mayar, C. Muller, J. Dortch, M. T. P. Gilbert, O. Lund, A. Wesolowska, M. Karmin, L. A. Weinert, B. Wang, J. Li, S. Tai, F. Xiao, T. Hanihara, G. van Driem, A. R. Jha, F.-X. Ricaut, P. de Knijff, A. B. Migliano, I. Gallego Romero, K. Kristiansen, D. M. Lambert, S. Brunak, P. Forster, B. Brinkmann, O. Nehlich, M. Bunce, M. Richards, R. Gupta, C. D. Bustamante, A. Krogh, R. A. Foley, M. M. Lahr, F. Balloux, T. Sicheritz-Pontén, R. Villems, R. Nielsen, J. Wang, E. Willerslev, An aboriginal Australian genome reveals separate human dispersals into Asia. *Science* **334**, 94–98 (2011).

88. T. S. Korneliussen, A. Albrechtsen, R. Nielsen, ANGSD: Analysis of next generation sequencing data. *BMC Bioinformatics* **15**, 356 (2014).

89. V. Pankratov, F. Montinaro, A. Kushniarevich, G. Hudjashov, F. Jay, L. Saag, R. Flores, D. Marnetto, M. Seppel, M. Kals, U. Võsa, C. Taccioli, M. Möls, L. Milani, A. Aasa, D. J. Lawson, T. Esko, R. Mägi, L. Pagani, A. Metspalu, M. Metspalu, Differences in local population history at the finest level: The case of the Estonian population. *Eur. J. Hum. Genet.* **28**, 1580–1591 (2020).

90. P. Skoglund, J. Storå, A. Götherström, M. Jakobsson, Accurate sex identification of ancient human remains using DNA shotgun sequencing. *J. Archaeol. Sci.* **40**, 4477–4482 (2013).

91. H. Li, A statistical framework for SNP calling, mutation discovery, association mapping and population genetical parameter estimation from sequencing data. *Bioinformatics* **27**, 2987–2993 (2011).

92. H. Weissensteiner, D. Pacher, A. Kloss-Brandstätter, L. Forer, G. Specht, H.-J. Bandelt, F. Kronenberg, A. Salas, S. Schönherr, HaploGrep 2: Mitochondrial haplogroup classification in the era of high-throughput sequencing. *Nucleic Acids Res.* **44**, W58–W63 (2016).

93. M. van Oven, M. Kayser, Updated comprehensive phylogenetic tree of global human mitochondrial DNA variation. *Hum. Mutat.* **30**, E386–E394 (2009).

94. M. Karmin, L. Saag, M. Vicente, M. A. Wilson Sayres, M. Järve, U. G. Talas, S. Rootsi, A.-M. Ilumäe, R. Mägi, M. Mitt, L. Pagani, T. Puurand, Z. Faltyskova, F. Clemente, A.

Cardona, E. Metspalu, H. Sahakyan, B. Yunusbayev, G. Hudjashov, M. DeGiorgio, E.-L. Loogväli, C. Eichstaedt, M. Eelmets, G. Chaubey, K. Tambets, S. Litvinov, M. Mormina, Y. Xue, Q. Ayub, G. Zoraqi, T. S. Korneliussen, F. Akhatova, J. Lachance, S. Tishkoff, K. Momynaliev, F.-X. Ricaut, P. Kusuma, H. Razafindrazaka, D. Pierron, M. P. Cox, G. N. N. Sultana, R. Willerslev, C. Muller, M. Westaway, D. Lambert, V. Skaro, L. Kovačevic, S. Turdikulova, D. Dalimova, R. Khusainova, N. Trofimova, V. Akhmetova, I. Khidiyatova, D. V. Lichman, J. Isakova, E. Pocheshkhova, Z. Sabitov, N. A. Barashkov, P. Nymadawa, E. Mihailov, J. W. T. Seng, I. Evseeva, A. B. Migliano, S. Abdullah, G. Andriadze, D. Primorac, L. Atramentova, O. Utevska, L. Yepiskoposyan, D. Marjanovic, A. Kushniarevich, D. M. Behar, C. Gilissen, L. Vissers, J. A. Veltman, E. Balanovska, M. Derenko, B. Malyarchuk, A. Metspalu, S. Fedorova, A. Eriksson, A. Manica, F. L. Mendez, T. M. Karafet, K. R. Veeramah, N. Bradman, M. F. Hammer, L. P. Osipova, O. Balanovsky, E. K. Khusnutdinova, K. Johnsen, M. Remm, M. G. Thomas, C. Tyler-Smith, P. A. Underhill, E. Willerslev, R. Nielsen, M. Metspalu, R. Villems, T. Kivisild, A recent bottleneck of Y chromosome diversity coincides with a global change in culture. *Genome Res.* **25**, 459–466 (2015).

95. G. D. Poznik, Y. Xue, F. L. Mendez, T. F. Willems, A. Massaia, M. A. W. Sayres, Q. Ayub, S. A. McCarthy, A. Narechania, S. Kashin, Y. Chen, R. Banerjee, J. L. Rodriguez-Flores, M. Cerezo, H. Shao, M. Gymrek, A. Malhotra, S. Louzada, R. Desalle, G. R. S. Ritchie, E. Cerveira, T. W. Fitzgerald, E. Garrison, A. Marcketta, D. Mittelman, M. Romanovitch, C. Zhang, X. Zheng-Bradley, G. R. Abecasis, S. A. McCarroll, P. Flicek, P. A. Underhill, L. Coin, D. R. Zerbino, F. Yang, C. Lee, L. Clarke, A. Auton, Y. Erlich, R. E. Handsaker, 1000 Genomes Project Consortium, C. D. Bustamante, C. Tyler-Smith, Punctuated bursts in human male demography inferred from 1,244 worldwide Y-chromosome sequences. *Nat. Genet.*, *48* , 593–599 (2016).

96. ISOGG, Y-DNA Haplogroup Tree 2019-2020; https://isogg.org/tree/.

97. YFull | Analysis and comparing your NextGen Y-Chr sequencing data. https://yfull.com/.

98. M. Mitt, M. Kals, K. Pärn, S. B. Gabriel, E. S. Lander, A. Palotie, S. Ripatti, A. P. Morris, A. Metspalu, T. Esko, R. Mägi, P. Palta, Improved imputation accuracy of rare and low-

frequency variants using population-specific high-coverage WGS-based imputation reference panel. *Eur. J. Hum. Genet.* **25**, 869–876 (2017).

99. A. R. Quinlan, BEDTools: The Swiss-army tool for genome feature analysis. *Curr. Protoc. Bioinformatics* **47**, 11.12.1-34 (2014).

100. S. Purcell, B. Neale, K. Todd-Brown, L. Thomas, M. A. R. Ferreira, D. Bender, J. Maller, P. Sklar, P. I. W. de Bakker, M. J. Daly, P. C. Sham, PLINK: A tool set for whole-genome association and population-based linkage analyses. *Am. J. Hum. Genet.* **81**, 559–575 (2007).

101. D. H. Alexander, J. Novembre, K. Lange, Fast model-based estimation of ancestry in unrelated individuals. *Genome Res.* **19**, 1655–1664 (2009).

102. R. Maier, P. Flegontov, O. Flegontova, U. Işıldak, P. Changmai, D. Reich, On the limits of fitting complex models of population history to f-statistics. *eLife* **12**, e85492 (2023).

103. N. Patterson, P. Moorjani, Y. Luo, S. Mallick, N. Rohland, Y. Zhan, T. Genschoreck, T. Webster, D. Reich, Ancient admixture in human history. *Genetics* **192**, 1065–1093 (2012).

104. C. Bronk Ramsey, Bayesian analysis of radiocarbon dates. *Radiocarbon* **51**, 337–360 (2009).

105. P. J. Reimer, W. E. N. Austin, E. Bard, A. Bayliss, P. G. Blackwell, C. B. Ramsey, M. Butzin, H. Cheng, R. Lawrence Edwards, M. Friedrich, P. M. Grootes, T. P. Guilderson, I. Hajdas, T. J. Heaton, A. G. Hogg, K. A. Hughen, B. Kromer, S. W. Manning, R. Muscheler, J. G. Palmer, C. Pearson, J. van der Plicht, R. W. Reimer, D. A. Richards, E. Marian Scott, J. R. Southon, C. S. M. Turney, L. Wacker, F. Adolphi, U. Büntgen, M. Capano, S. M. Fahrni, A. Fogtmann-Schulz, R. Friedrich, P. Köhler, S. Kudsk, F. Miyake, J. Olsen, F. Reinig, M. Sakamoto, A. Sookdeo, S. Talamo, The IntCal20 northern hemisphere radiocarbon age calibration curve (0–55 cal kBP). *Radiocarbon* **62**, 725–757 (2020).

106. N. Kotova, *Neolithization in Ukraine* (BAR Publishing, 2003).

107. P. P. Tolochko, Ed., *Україна: хронологія розвитку* [Ukraine: Chronology of Development] (KVIS, Kyiv, 2008).

108. S. I. Andrukh, G. N. Toshchev, *Могильник Мамай-Гора. Книга I* [Mamai-Hora Cemetery. Book I] (Zaporizhzhia National Univ., Zaporozhzhia, 1999).

109. S. I. Andrukh, *Могильник Мамай-Гора. Книга II* [Mamai-Hora Cemetery. Book II] (Zaporizhzhia National Univ., Zaporozhzhia, 2001).

110. S. I. Andrukh, G. N. Toshchev, *Могильник Мамай-Гора. Книга III* [Mamai-Gora Burial Site. Book III] (Zaporizhzhia National Univ., Zaporozhzhia, 2004).

111. S. I. Andrukh, G. N. Toshchev, *Могильник Мамай-Гора. Книга IV* [Mamai-Gora Burial Site. Book IV] (Zaporizhzhia National Univ., Zaporozhzhia, 2009).

112. S. I. Andrukh, G. N. Toshchev, A. G. Toshchev, *Могильник Мамай-Гора. Книга V* [Mamai-Gora Burial Site. Book V] (Zaporizhzhia National Univ., Zaporozhzhia, 2023).

113. G. N. Toščev, *Die Neolithische Nekropole Mamaj-Gora Im Unteren Dneprgebiet* (Godišnjak vol. XXXIV, Centar za balkanološka ispitivanja, Sarajevo, 2005).

114. V. K. Mikheev, *Отчет о работе Средневековой археологической экспедиции Харьковского государственного университета им. А.М.Горького в 1982 году* [Report on the work of the Medieval Archaeological Expedition of the Kharkiv State University named after A. M. Gorky in 1982] (Institute of Archeology of the National Academy of Sciences of Ukraine, Kyiv, 1983).

115. Y. V. Buinov, V. K. Mikheev, Курган срубной культуры у с. Сухая Гомольша на Харьковщине [Kurgan of the Timber-Frame Culture near the Village of Sukhaya Gomolsha in the Kharkiv Region]. *Bull. Kharkiv Univ. Hist. Ser.* **343**, 87–95 (1989).

116. V. P. Vanchugov, “Памятники тудоровского типа в Северо-Западном Причерноморье (К вопросу о белозерской культуре)” [“Landmarks of the Tudor Type in the Northwestern Black Sea Region (On the Issue of the Bilozerska Culture)”] in *Межплеменные связи эпохи бронзы на территории Украины* [Intertribal Relations of the Bronze Age on the Territory of Ukraine] (Naukova Dumka, Kyiv, 1987), pp. 114–131.

117. V. P. Vanchugov, *Белозерские памятники в Северо-Западном Причерноморье: проблема формирования белозерской культуры* [Bilozerska Landmarks in the North-Western Black Sea Region: The Problem of the Formation of the Bilozerska culture] (Naukova Dumka, Kyiv, 1990).

118. V. V. Otroshchenko, "Белозерская культура" ["Bilozerska culture"] in *Культуры эпохи бронзы на территории Украины* [Cultures of the Bronze Age on the territory of Ukraine], S. S. Berezanskaya, V. V. Otroshchenko, N. N. Cherednichenko, I. N. Sharafutdinova, Eds. (Naukova Dumka, Kyiv, 1986).

119. S. M. Agulnikov, Хронология и периодизация белозерских памятников Пруто-Днестровского междуречья [Chronology and periodization of Bilozerska culture landmarks of the Prut-Dniester interfluve]. *Rev. Arheol.* **1**, 77–91 (2005).

120. K. Gorbenko, Building constructions of southern part of "Citadel" of dykyi sad ancient settlement. *Eminak* **1**, 36–66 (2021).

121. K. Gorbenko, O. Trygub, History of Exploration of Final Bronze Age Fortified Settlement (Hillfort) "Dykyi Sad". *Rev. Arheol.* **18**, 17–34 (2022).

122. S. I. Kruts, "Антропологические особенности населения срубной культуры территории Украины" ["Anthropological features of the Srubna culture population on the territory of Ukraine"] in *Энеолит и бронзовый век Украины* [The Eneolithic and Bronze Age of Ukraine], D. Ya. Telegin, Ed. (Naukova Dumka, Kyiv, 1976), pp. 222–232.

123. S. I. Kruts, *Скифы степей Украины по антропологическим данным* [Scythians of the Ukrainian Steppes According to Anthropological Data] (Kurgans of Ukraine, Publisher Oleh Filyuk, Kyiv-Berlin, 2017), vol. 25.

124. K. V. Gorbenko, Y. S. Grebennikov, "The 'Dykyi Sad' fortified settlement as a uniting link in the context of economic, political, and cultural relation of 1200 – 1000 BC (the Baltic shore, the Pontic region and the Mediterranean)" in *Routes between the Seas: Baltik-Boh-*

Bug-Pont from the 3rd to the Middle of the 1st Millennium BC (Baltic-Pontic Studies vol. 14, Uniwersytet im. Adama Mickiewicza, 2009).

125. K. Gorbenko, I. Pistruil, Structure no 25 at "Dykyi Sad" settlement. *Eminak* **1**, 324–341 (2020).

126. M. Bandrivskyi, L. Krushelnytska, "Основні періоди розвитку висоцької культури (За матеріалами поховальних пам'яток)" ["The main periods of the development of Vysotska culture (Based on the materials of burial landmarks)"] in *Записки наукового товариства Шевченка SSXXV* [Notes of the Shevchenko Scientific Society SSXXV] (Shevchenko Scientific Society, Lviv, 1998).

127. M. Bandrivskyi, *Могильник в Петрикові біля Тернополя в контексті поховального обряду висоцької культури* [Graveyard in Petrykiv Near Ternopil in the Context of the Funeral Rite of Vysotska Culture] (National Academy of Sciences of Ukraine, Lviv, 2012).

128. D. Pavliv, "Нові пам'ятки «лужицької» культури на заході України" ["New landmarks of 'Lusatian' culture in the western part of Ukraine" in *Пам'ятки гальштатського періоду межиріччя Вісли, Дністра і Прип`яті* [Landmarks of the Hallstatt Period in the Vistula, Dniester and Pripyat Rivers Interfluve], L. Krushelnytska, Ed. (Naukova Dumka, Kyiv, 1993), pp. 11–56.

129. D. Pavliv, Пам'ятки кінця доби бронзи – початку ранньозалізної доби із с. Ульвівок на Сокальщині [Landmarks of the end of the Bronze Age—The beginning of the Early Iron Age from the village of Ulvivok in Sokal region]. *Mat. Res. Archeol. Prykarpattia Volhynia* **10**, 154–165 (2006).

130. D. Pavliv, Ульвівецькі келихи [Ulvivetski goblets]. *Mat. Res. Archeol. Prykarpattia Volhynia* **12**, 68–102 (2008).

131. D. Pavliv, Особливі форми кераміки з могильників ульвівецько-рованцівського типу [Special forms of ceramics from grave sites of Ulvivetsk-Rovantsi type]. *Mat. Res. Archeol. Prykarpattia Volhynia* **22**, 111–122 (2018).

132. D. Pavliv, Пам’ятки археології села Ульвівок та околиць у дослідженнях львівських вчених [Monuments of archeology of the village of Ulvivok and its surroundings in the research of Lviv scientists]. *Mat. Res. Archeol. Prykarpattia Volhynia* **23**, 337–361 (2019).

133. D. Pavliv, «Жертовні» чаші в похованнях доби бронзи та раннього заліза [‘Sacrificial’ bowls in Bronze Age and Early Iron Age burials]. *Mat. Res. Archeol. Prykarpattia Volhynia* **26**, 57–83 (2022).

134. D. N. Kozak, D. Yu, Pavliv, Могильник ульвівецького типу поблизу с. Городок на Волині [Burial ground of Ulvivetskogo type near the village. A small town in Volyn]. *Archaeology* **3**, 71–81 (1999).

135. D. Pavliv, Семантика зображень на фунеральному посуді з Рованцівського могильника кінця доби бронзи [Semantics of images on funerary vessels from the Rovantsiv cemetery of the end of the Bronze Age]. *Mat. Res. Archeol. Prykarpattia Volhynia* **24**, 88–116 (2020).

136. S. O. Horbenko, “Матеріали до реконструкції за черепом зовнішнього вигляду людини I тис. до н. е. ульвівецької культури з могильника поблизу с. Рованці” [“Materials for reconstruction of the human appearance from the skull of the 1st millennium BC. e. of the Ulvivec culture from the burial ground near the village of Rovantsi”] in *Current Issues of Orthopedic Dentistry* (UMDA, Poltava, 1996), pp. 125–126.

137. I. B. Shramko, I. V. Golubeva, S. A. Zadnikov, K. Yu. Pelyashenko, Звіт про наукові археологічні експертизи в Харківській області та в м. Харкові в 2010 [Report on scientific archaeological expertise in the Kharkiv region and the city of Kharkiv in 2010] (Museum of Archeology of V.N. Karazin Kharkiv National Univ., Kharkiv, 2011).

138. I. B. Shramko, S. A. Zadnikov, K. Yu. Pelyashenko, Yu. V. Buynov, Отчет об охранных раскопках курганного могильника «Кумы» на территории Красногорадского района Харьковской области [Report on the protective excavations of the kurgan burials “Kumy” in the Krasnohrad district of the Kharkiv region] (Museum of Archeology of V.N. Karazin Kharkiv National University, Kharkiv, 2011).

139. I. B. Shramko, "Рятівні дослідження курганного могильника Куми на території Красноградського району Харківської області" ["Rescue studies of the Kumy kurgan burial in the Krasnograd district of the Kharkiv region"] in *Археологічні дослідження в Україні – 2010* [Archaeological research in Ukraine – 2010], D. N. Kozak, Ed. (Kyiv, Poltava, 2011), p. 378.

140. I. V. Bruyako, О пользе археологии в поисках сущего (заметки о фактическом содержании понятия «фрако-киммерийский» горизонт) [Archeology in search of the real (notes on the concept actuality of the 'Thracian-Cimmerian' horizon)]. *Stratum Plus. Archeol. Cult. Anthropol.* **3**, 155–190 (2013).

141. I. V. Bruyako, "Могильник эпохи среднего гальштата «Картал-III» на Нижнем Дунае" ["Kartal-III burial ground of the Middle Hallstatt period on the Lower Danube"] in *Ранній залізний вік Євразії. До 100-річчя О. І.Тереножкіна. М-ли Міжнародної наукової конф. (16-19 травня 2007 р.)* [Early Iron Age of Eurasia. To the 100th anniversary of O. I. Terenozhkin. Issues of the International Scientific Conf. (May 16-19, 2007)] (VPD Format, Kyiv-Chigyrin, 2007), pp. 40–43.

142. I. Shramko, M. Tarasenko, Egyptian imports of 6th century BC in the materials of Forest-Steppe Scythia. *Skhodoznavstvo* **89**, 139–180 (2022).

143. G. T. Kovpanenko, S. S. Bessonova, S. A. Skory, *Памятники скифской эпохи днепровского лесостепного правобережья* [Landmarks of the Scythian Era of the Dnipro Right Bank Forest-steppe] (Naukova Dumka, Kyiv, 1989).

144. S. A. Skory, *Киммерийцы в украинской лесостепи* [Cimmerians in the Ukrainian Forest-steppe] (Archaeology, Kyiv-Poltava, 1999).

145. E. V. Maksimov, E. A. Petrovskaya, *Древности скифского времени Киевского Поднепровья* [Antiquities of the Scythian Period of the Kyiv Dnipro Region] (Institute of Archaeology of the National Academy of Sciences of Ukraine, Poltava, 2008).

146. G. T. Kovpanenko, *Курганы раннескифского времени в бассейне р. Рось* [Kurgans of the Early Scythian Period in the Ros River Basin] (Naukova Dumka, Kyiv, 1981).

147. B. M. Levchenko, N. B. Levchenko, D. S. Grechko, Курганы раннескифского времени у с. Медвин в Поросье (по материалам раскопок 1984—1985 гг.) [Kurgans of the Early Scythian period near the Medvin village in Porossia (based on materials from excavations in 1984-1985)]. *Archeol. Ancient Hist. Ukraine* **2**, 202–218 (2015).

148. D. S. Grechko, Об особых видах погребений у населения восточноевропейской Лесостепи VII—IV вв. до н. э. [On special burial types of the East European Forest-Steppe population in the 7th-4th centuries BC]. *Antiquities* **13**, 181–200 (2015).

149. I. I. Zayets, Курганный комплекс раннескифского времени у с. Тютьки Винницкой области [Kurgan complex of the early Scythian period near the Tyutki village in Vinnytsia region]. *Soviet Archaeol.* **1**, 256–260 (1979).

150. M. T. Kashuba, N. V. Goltseva, Сахарнянский могильник I (Цыглэу) [Sakharnyansky burial ground I (Tsygleu)]. *Soviet Archaeol.* **1**, 197–209 (1991).

151. V. V. Romanyuk, D. S. Grechko, O. D. Mohylov, Дослідження курганів раннього залізного віку в Пороссі [Research of Early Iron Age kurgans in Porossia]. *Archeol. Ancient Hist. Ukraine* **2**, 251–263 (2018).

152. G. T. Kovpanenko, "Курганы скифского времени у с. Медвин в Поросье" ["Mounds of the Scythian period in the village of Medvyn in Porossia"] in *Скифы и сарматы* [Scythians and Sarmatians], A. I. Terenozhkin, Ed. (Naukova Dumka, Kyiv, 1977), pp. 40–72.

153. G. T. Kovpanenko, *Племена скіфського часу на Ворсклі* [Tribes of Scythian Times on Vorskla River] (Naukova Dumka, Kyiv, 1967).

154. B. A. Shramko, *Бельское городище скифской эпохи: город Гелон* [Bilsk Settlement of the Scythian Era: The City of Gelon] (Naukova Dumka, Kyiv, 1987).

155. S. V. Makhortyh, Погребальные сооружения и обряд курганных некрополей Бельского городища [Burial structures and rites of kurgan necropolises of the Bilsk settlement]. *Stratum Plus* **3**, 223–256 (2013).

156. Yu. M. Boyko, *Соціальний склад населення басейну р. Ворскли за скіфської доби* [Social Structure of the Population of the Vorskla River Basin in the Scythian Period] (CSP of the National Academy of Sciences of Ukraine and UTOPiK, Kotelva-Kyiv, 2017).

157. Yu. N. Boyko, S. I. Berestnev, *Погребения VII-IV вв. до н. э. курганного могильника у с. Купьеваха (Ворсклинский регион скифского времени)* [Burials of the 7th-4th centuries BC of the kurgan burial near the Kupyevakha village (Vorskla river region of the Scythian period)] (RA-Karavella, Kharkiv, 2001).

158. B. A. Shramko, Новые раскопки курганов в могильнике Скоробор [New excavations of kurgans in the Skorobor burial ground]. *Antiquities* **1**, 102–126 (1994).

159. B. A. Shramko, Розкопки курганів VII—IV ст. до н. е. поблизу Більська [Excavation of burial mounds VII-IV centuries. BC e. near Bilsk]. *Archaeology* **4**, 117–133 (1994).

160. S. A. Zadnikov, I. B. Shramko, *Більське городище в наукових працях Б. А. Шрамка: збірник наукових праць, присвячений 95-річчю від дня народження вченого* [Bilsk Settlement in the Scientific Works of B. A. Shramko: A Collection of Scientific Works Dedicated to the 95th Anniversary of the Scientist's Birth] (V.N. Karazin Kharkiv National University-Bilsk ICZ, Maidan-Kotelva-Kharkiv, 2016).

161. I. B. Shramko, S. A. Zadnikov, “Работы на Западном укреплении Бельского городища” [“Works on the Western Fortification of the Bilsk Settlement”] in *Археологічні дослідження в Україні 2011 р.* [Archaeological Research in Ukraine 2011], D. N. Kozak, Ed. (Institute of Archaeology of the National Academy of Sciences of Ukraine, Kyiv, 2012), p. 391.

162. I. B. Shramko, S. A. Zadnikov, “Дослідження Більського городища” [“Investigation of the Bilsk settlement” in *Археологічні дослідження в Україні 2013 р.* [Archaeological research in Ukraine 2013], D. N. Kozak, Ed. (Institute of Archaeology of the National Academy of Sciences of Ukraine, Kyiv, 2014), pp. 225–226.

163. V. V. Pryimak, I. B. Shramko, S. A. Zadnikov, "Дослідження курганів у могильнику Скоробір" ["Investigation of barrows in the Skorobir burial ground"] in *Археологічні дослідження в Україні 2013 р.* [Archaeological Research in Ukraine 2013], D. N. Kozak, Ed. (Institute of Archaeology of the National Academy of Sciences of Ukraine, Kyiv, 2013), pp. 212–213.

164. I. B. Shramko, Колчанный набор из погребения конца VI в. до н. э. могильника Скоробор [Quiver set from burial of the end of the 6th century in Skorobir cemetery]. *Archeol. Ancient Hist. Ukraine* **2**, 270–279 (2016).

165. I. Shramko, Bilsk (Belsk) city-site: The largest fortified settlement of Scythia. *Ancient West East* **20**, 171–218 (2021).

166. I. Shramko, S. Zadnikov, The Bilsk fortified settlement and the Hallstatt world. *Silesian Archaeol. Rep.* **63**, 123–148 (2021).

167. V. E. Radzievskaya, "Некоторые итоги раскопок Коломакского городища" ["Some Results of the Excavations of the Kolomak Settlement"] in *Археологические исследования на Украине в 1978–1979 гг. Тезисы докладов XVIII конференции Института археологии АН УССР, Днепропетровск, апрель 1980 г* [Archaeological Research in Ukraine in 1978–1979. Abstracts of the Reports of the XVIII Conference of the Institute of Archaeology of the Academy of Sciences of the Ukrainian SSR, Dnepropetrovsk, April 1980], V. F. Gening, Ed. (Institute of Archaeology of the Academy of Sciences of Ukrainian SSR, Dnepropetrovsk, 1980), p. 82.

168. V. E. Radzievskaya, "Раскопки Коломакского городища" ["Excavations of the Kolomak settlement"] in *Археологические открытия 1980 г. Москва* [Archaeological discoveries of 1980. Moscow] (Institute of Archaeology of the RAS, Moscow, 1981), pp. 305–306.

169. V. E. Radzievskaya, "Основные итоги раскопок Коломакского городища" ["The Main Results of the Excavations of the Kolomak Settlement"] in *История и археология Слободской Украины. Тезисы докладов и сообщений Всеукраинской конференции, посвященной 90-летию XII Археологического съезда* [History and Archaeology of Sloboda Ukraine. Abstracts of Reports and Communications of the All-Ukrainian

Conference Dedicated to the 90th Anniversary of the XII Archaeological Congress], V. K. Mikheev, Ed. (Kharkiv State University, Kharkiv, 1992), pp. 177–179.

170. S. A. Zadnikov, V. E. Radzievskaya, "Античний керамічний імпорт Коломацького городища" ["Ancient ceramic imports of the Kolomatsky settlement"] in *Проблеми історії та археології України: Матеріали XI Всеукраїнської наукової конференції, до 60-річчя від початку дослідження Більського городища Харківським університетом (Харків, 14– 15 грудня 2018 р.)* [Problems of the History and Archeology of Ukraine: Materials of the XI All-Ukrainian Scientific Conference, to the 60th Anniversary of the Beginning of Research on the Bilsky Settlement by Kharkiv University (Kharkiv, 14–December 15, 2018)] (Kharkiv National University, Kharkiv, 2018), p. 16.

171. Y. N. Boyko, "Новые исследования курганного могильника у с. Купьеваха" ["New studies of the kurgan burial in the village of Kupyevakha" in *Древности 2006–2008* [Antiquities 2006–2008] (NTMT, Kharkiv, 2008), pp. 204–225.

172. A. V. Bandurovsky, Yu. V. Buinov, *Курганы скифского времени Харьковской области (северскодонецкий вариант)* [Scythian Kurgan Burials of the Kharkiv Region (Northern Donets type)] (Institute of Archaeology of the National Academy of Sciences of Ukraine, Kyiv, 2000).

173. A. P. Medvedev, "Новый аспект этногеографии Древней Скифии (меланхлены и будины)" ["A new aspect of the ethnogeography of ancient Scythia (Melanchlaenians and Budins)"] in *Исторические Записки: Научные Труды Исторического Факультета Воронежского Государственного Университета* [Historical Notes: Scientific Works of the Faculty of History, Voronezh State University], (Voronezh State University, Voronezh, 2005), pp. 92–103.

174. B. A. Shramko, "Люботинское городище" ["Lyubotinsky fortified settlement"] in *Люботинское городище* [Lyubotinsky Fortified Settlement], Yu. V. Buinov, Ed. (Region-inform, Kharkiv, 1998), pp. 9–131.

175. I. B. Shramko, Исследование округи Люботинского городища [Research of the Lyubotinsky settlement]. *Archaeol. Chro. Left Bank Ukraine* **1**, 102–108 (2003).

176. I. B. Shramko, S. A. Zadnikov, A. O. Zorya, Селище скифского времени у с. Червоносово [Village of the Scythian era in the village of Chervonosovo]. *Antiquities* **5**, 27–32 (2004).

177. D. S. Grechko, *Населення скіфського часу на Сіверському Дінці* [Population of the Scythian Period in Siversky Donets Basin] (Institute of Archeology of the National Academy of Sciences of Ukraine, Kyiv, 2010).

178. Yu. V. Buinov, A. V. Bandurovsky, V. M. Okatenko, "Исследование курганов скифской эпохи в Харькьковской области" ["Kurgans of the Scythian period in the Kharkiv region"] in *Археологічні дослідження в Україні 2003–2004 рр.* [Archaeological Research in Ukraine 2003–2004], N. O. Gavrilyuk, Ed. (Wild Field, Zaporizhzhia, 2005), pp. 9–12.

179. A. V. Bandurovsky, Yu. V. Buinov, A. K. Degtyar, "Новые исследования курганов скифского времени в окрестностях г. Люботина" ["New studies of the kurgans of the Scythian period in the vicinity of the town of Lyubotin"] in *Люботинское городище* [Lyubotinsky Settlement], Yu. V. Buinov, Ed. (Region-inform, Kharkiv, 1998), pp. 143–182.

180. A. P. Medvedev, О новом аспекте проблемы будинов и меланхленов (в свете последних публикаций могильников скифского времени на Северском Донце) [On a new aspect of the problem of Budins and Melankhlens (in the light of the latest publications of burial sites of the Scythian period on the Siversky Donets)]. *Archaeol. Chro. Left Bank Ukraine* **3**, 21–34 (2006).

181. V. M. Okatenko, "Додаткові дослідження курганів Караванської групи Люботинського могильника" ["Additional studies of the kurgans of the Caravan group of the Lyubotyn burial"] in *Археологічні дослідження в Україні 2013* [Archaeological Research in Ukraine 2013], D. N. Kozak, Ed. (Institute of Archaeology of the National Academy of Sciences of Ukraine, Kyiv, 2014), p. 257.

182. D. S. Grechko, A. V. Shelehan, *Гришковский могильник скифов на Харьковщине* [Grishkovsky Burial Ground of the Scythians in the Kharkiv Region] (Institute of Archaeology of the National Academy of Sciences of Ukraine, Kyiv, 2012).

183. B. M. Levchenko, “Исследование первой курганной группы у с. Медвин на Богуславщине (раскопки. 1982 г., 1984—85 гг.)” [“Study of the first kurgan group near the village of Medvin in Boguslav region (excavations. 1982, 1984-85)”] in *Гришковский могильник скифов на Харьковщине* [Grishkovsky Burial Ground of the Scythians in Kharkiv Region], D. S. Grechko, A. V. Shelehan, Eds. (Institute of Archaeology of the National Academy of Sciences of Ukraine, Kyiv, 2012).

184. L. I. Babenko, *Песочинский курганный могильник скифского времени* [Pesochinsky Kurgan Burial of the Scythian Period] (Raider Publishing House, Kharkiv, 2005).

185. B. A. Shramko, Курганы у с. Веселое в Харьковской области [Kurgans near the village of Veseloe in the Kharkiv region]. *Bull. Kharkiv Univ. Hist.* **362**, 110–119 (1992).

186. S. I. Andrukh, G. N. Toshchev, “Могильник скифского времени Мамай-Гора” [“The burial ground of the Scythian time Mamai-Gora”] in *Peregrinationes Archaeologicae in Asia et Europa Joanni Chochorowski Dedicatae*, W. Blajer, Ed. (Wydawnictwo Profil-Archeo/Uniwersytet Jagielloski Instytut Archeologii, Kraków, 2012), pp. 485–490.

187. S. I. Andrukh, G. N. Toshchev, Раннескифский комплекс могильника Мамай-Гора в Нижнем Поднепровье [Early Scythian complex of the Mamai-Gora cemetery in Lower Dnieper]. *Stratum Plus* **3**, 405–421 (2022).

188. A. E. Puzdrovsky, *Крымская Скифия II в. до н. э. III в. н. э: погребальные памятники* [Crimean Scythia II c. BCE to III c. CE: Funerary Monuments] (Business-Inform, Simferopol, 2007).

189. V. I. Mordvintseva, «Пізньоскіфська культура Криму»: виникнення і еволюція концепту [“Late Scythian culture of the Crimea”: Emergence and evolution of the concept]. *Archaeology* **2**, 113–126 (2018).

190. V. A. Latysheva, *Отчет о раскопках экспедиции Харьковского госуниверситета в Северо-Западном Крыму в 1976 г* [Report on the excavations of the Kharkiv State University expedition in North-Western Crimea in 1976] (Institute of Archeology of the National Academy of Sciences of Ukraine, Kyiv, 1976).

191. V. V. Kotenko, *Поселення херсонеської хори Маслини у Північно-Західній Тавриці* [Settlement of the Chersonese Choir Maslyna in North-Western Taurica] (V.N. Karazin National University, Kharkiv, 2017).

192. D. S. Grechko, S. B. Lantsov, "Курганный могильник у пгт. Новоозерное в Северо-Западном Крыму" ["Kurgan burial near the town of Novoozernoe in North-West Crimea"] in *Наукові записки* [Scientific Notes. Historical Sciences. Volume 21] (Kyrovohrad State Pedagogical University named after Volodymyr Vinnichenko, Kyrovohrad, 2014), pp. 182–195.

193. O. I. Smirnov, "Александровский – новый могильник хоры Ольвии в г. Николаев" ["Aleksandrovsky – a new burial ground of the Olbia choir in the city of Nikolaev"] in *Древности Северного Причерноморья III-II вв. до н.э./ Междунар. науч. конф., Тирасполь, 16-19 окт. 2012 г.* [Antiquities of the Northern Black Sea Region of the 3rd-2nd Centuries. BC/Int. Scientific Conf., Tiraspol, 16-19 Oct. 2012], N. P. Telnov, Ed. (Transnistrian State University named after T.G. Shevchenko, Tiraspol, 2012), pp. 149–165.

194. O. I. Smirnov, A. V. Ivchenko, K. V. Gorbenko, "Исследование на могильнике позднеклассического времени в г. Николаеве" ["Research at the burial ground of the late classical period in Nikolaev"] in *Археологічні дослідження в Україні 2011 р.* [Archaeological Research in Ukraine 2011] (Volyn antiquities, Kyiv-Lutsk, 2012), pp. 343–346.

195. O. I. Smirnov, "Античний некрополь у місті Миколаєві" ["Ancient necropolis in the city of Mykolaiv"] in *Краєзнавчий альманах. Історія. Археологія. Наука. Культура. Освіта.* [Regional History Almanac. History. Archeology. Science. Culture. Education] (OIPPO, Mykolaiv, 2013), pp. 56–61.

196. O. I. Smirnov, "«Александровский» - античный некрополь в городе Николаеве" ["'Aleksandrovsky' - an ancient necropolis in the city of Nikolaev"] in *Науковий вісник Миколаївського національного університету імені В.О.Сухомлинського: Збірник наукових праць* [Scientific Bulletin of the Mykolaiv National University Named after V. O. Sukhomlynskyi: Collection of Scientific Works] (MNU, Mykolaiv, 2013), pp. 22–31.

197. O. I. Smirnov, A. V. Ivchenko, “Античный могильник IV в. до н.э. в г. Николаеве” [“Ancient cemetery of the 4th c. BC in the city of Mykolaiv”] in *IX Миколаївська обласна краєзнавча конференція. «Історія. Етнографія. Культура. Нові дослідження»* [The IX Mykolaiv Regional Regional Conference. 'History. Ethnography. Culture. New research.'] (Iryna Gudym Publishing House, Mykolaiv, 2015), pp. 43–44.

198. O. I. Smirnov, L. I. Smirnov, “Нова антична пам’ятка археології на території м. Миколаєва” [“A new ancient monument of archeology on the territory of Mykolaiv”] in *Стародавнє Причорномор’є. Випуск XI* [Ancient Black Sea Region. Issue XI], I. V. Nemchenko, Ed. (Odessa National University named after I. I. Mechnikov, Odesa, 2016), pp. 499–505.

199. O. Smirnov, До дискусії про ідентифікацію античних могильників у місті Миколаєві (на прикладі могильника «Олександрівський» і «Старофлотські казарми») [To the discussion on the identification of ancient burial grounds in the city of Mykolaiv (on the example of the 'Olexandrivskyi' burial ground and 'Staroflot barracks')]. *Eminak* **4**, 210–218 (2016).

200. V. K. Myheev, A. A. Moruzhenko, B. A. Shramko, “Исследования в бассейне р. Мерлы” [“Research in the basin of the Merly River”] in *Археологические открытия 1974 года* [Archeological Discoveries of 1974], B. A. Rybakov, Ed. (Nauka, Moscow, 1975), pp. 324–325.

201. V. E. Radzievskaya, B. A. Shramko, Нові археологічні пам’ятки на Харківщині [New archaeological monuments in the Kharkiv region]. *Archaeology* **33**, 100–108 (1980).

202. R. M. Reida, A. V. Geiko, S. V. Sapyegin, Поховання з «платою Харону» з Шишацького могильника черняхівської культури [Burials with 'Payment to Charon' from the Shishatsky burial ground of the Chernyakhov culture]. *Archaeology* **1**, 97–103 (2014).

203. V. D. Baran, Ed., *Славяне Юго-Восточной Европы в предгосударственный период* [Slavs of South-Eastern Europe in the Pre-state Period] (Naukova Dumka, Kyiv, 1990).

204. O. V. Petrauskas, Die Gräberfelder der Černjachov—Kultur von Kosanovo und Gavrilovka — eine vergleichende Studie zu Chronologie, Bestattungssitten und ethnokulturellen Besonderheiten. *Bericht der Römisch—Germanischen Kommission* **83**, 224–351 (2003).

205. M. V. Lyubichev, Погребения с позднескифскими/сарматскими признаками на могильниках черняховской культуры Днепро-Донецкой лесостепи [Burials with Late Scythian/Sarmatian Features at the Chernyakhov Culture Cemeteries of the Dnieper-Donets Forest-Steppe]. *OIUM* **1**, 33–52 (2011).

206. T. S. Konduktorova, *Антропология древнего населения Украины (I тыс. до н.э. — середина I тыс. н.э.)* [Anthropology of the Ancient Population of Ukraine (1st Millennium BC — Mid-1st Millennium AD)] (Nauka, Moscow, 1972).

207. T. O. Rudych, *Населення Середнього Подніпров'я I—II тисячоліття за матеріалами антропології* [Population of the Middle Dnipro Region of the 1st-2nd Millennium According to Anthropological Materials] (Institute of Archeology of the National Academy of Sciences of Ukraine, Kyiv, 2014).

208. V. Bierbrauer, Archäologie Und Geschichte der Goten vom 1.–7. Jahrhundert. *Fruehmittelalterliche Studien* **28**, 51–171 (1994).

209. M. B. Shchukin, *Готский путь* [The Gothic Way] (St. Petersburg State Univ., St. Petersburg, 2005).

210. M. V. Lyubichev, *Черняховская культура Днепро-Донецкой лесостепи: История исследования и основные проблемы изучения* [Chernyakhiv Culture of the Dnipro-Donetsk Forest-steppe: History of Studies and Main Problems of Study] (KhGADTU, Kharkiv, 2000).

211. N. S. Abashina, B. V. Magomedov, R. M. Reida, *Пам'ятки черняхівської культури Полтавської області* [Landmarks of the Chernyakhov Culture in the Poltava Region] (Institute of Archeology of the National Academy of Sciences of Ukraine, Kyiv, 2019).

212. E. V. Makhno, “Типи поховань та планування Компаніївського могильника” [“Types of burials and layout of the Companijiv burial ground”] in *Середні віки на Україні* [Middle Ages in Ukraine], F. P. Shevchenko, Ed. (Naukova Dumka, Kyiv, 1971), pp. 87–95.

213. R. Reida, A. Geiko, S. Sapyegin, Конструктивні характеристики інгумаційних поховань черняхівської культури Шишацького могильника [Structural characteristics of inhumation burials of the Chernyakhiv culture of the Shishatsky cemetery]. *Archeol. Ancient Hist. Ukraine* **1**, 23–29 (2019).

214. O. V. Kovalenko, R. S. Lugovii, Пізньосарматські поховання Сторожівського курганного могильника [Late Sarmatian burials of the Storozhiv kurgan burial]. *Archaeol. Annals Left Bank Ukraine* **1**, 34–44 (2008).

215. M. Rudynskyi, Кантемирівські могили римської доби [Kantemyriv graves of the Roman period]. *Notes All-Ukrainian Archaeol. Commit.* **1**, 29–62 (1930).

216. A. Geiko, S. Korinnyi, R. Bulavenko, R. Reida, S. Sapyegin, Перший досвід дослідження глиняного посуду черняхівської культури за допомогою методів ДНК-технології [The first experience of researching earthenware of the Chernyakhiv culture using DNA technology]. *Archaeolo. Ceramol.* **1**, 65–74 (2020).

217. R. M. Reida, A. V. Geiko, S. V. Sapyegin, Деякі хроноіндикатори із Шишацького могильника черняхівської культури [Some chronoindicators from the Shishatsky cemetery of the Chernyakhov culture]. *Ostrogothica* **3**, 111–120 (2018).

218. R. M. Reida, A. V. Heiko, S. V. Sapiehin, Скляний кубок з еклектичними ознаками з поховання 112 Шишацького могильника [A glass cup with eclectic features from burial 112 of the Shishatsky cemetery]. *Archaeology* **4**, 95–109 (2021).

219. R. M. Reida, A. V. Geiko, S. V. Sapyegin, Поховання 115 Шишацького могильника зі скляним орнаментованим кубком [Burial 115 of Shishatsky cemetery with a glass ornamented cup]. *OIUM* **5**, 20–28 (2016).

220. O. V. Petrauskas, Деякі підсумки досліджень комплексу пам'яток пізньоримського часу біля с. Комарів [Some results of research on the complex of monuments of the late Roman period near the village of Komariv]. *OIUM* **4**, 165–184 (2014).

221. M. Y. Smishko, Поселення III—IV ст. н. е., із слідами скляного виробництва біля с. Комарів Чернівецької області [Settlement of the III-IV centuries CE with traces of glass production near the Komariv village of Chernivtsi region]. *Mat. Res. Archeol Prykarpattia Volyn* **5**, 67–80 (1964).

222. B. V. Magomedov, S. V. Didenko, "Могильник черняховской культуры у с. Легедзино. Работы 2008—2009 гг" ["The Chernyakhov culture burial ground near the village of Legedzino. Works of 2008–2009"] in *Трипольское поселение-гигант Тальянки. Исследования 2009 г* [The Trypillian Giant Settlement of Talyanki. Research of 2009], V. A. Kruts, A. G. Korvin-Piotrovsky, F. Menotti, S. N. Ryzhov, D. V. Tolochko, V. V. Chabanyuk, Eds. (Institute of Archeology of the National Academy of Sciences of Ukraine, Kyiv, 2009), pp. 56–92.

223. B. V. Magomedov, S. V. Dydenko, "Могильник черняховской культуры у с. Легедзино. Работы 2011 года" ["Cemetery of Chernyakhov culture in the village of Legedzino. Works of 2011"] in *Трипольское поселение-гигант Тальянки. Исследования 2011 г* [Trypillian Giant Settlement Talyanki. Research 2011], V. A. Kruts, A. G. Korvyn-Piotrovsky, S. N. Ryzhov, D. K. Chernovol, V. V. Chabanyuk, Eds. (Institute of Archeology of the National Academy of Sciences of Ukraine, Kyiv, 2011), pp. 86–105.

224. I. B. Shramko, I. V. Golubeva, S. A. Zadnikov, K. Yu. Pelyashenko, *Звіт про археологічні розвідки в Харківській області та в м. Харкові в 2008 р* [Report on archaeological explorations in the Kharkiv region and the city of Kharkiv in 2008] (Museum of Archeology of V.N. Karazin Kharkiv National, Kharkiv, 2009).

225. I. B. Shramko, I. V. Golubeva, S. A. Zadnikov, V. M. Okatenko, K. Yu. Pelyashenko, "Археологічні розвідки в м. Харків і Харківській обл. у 2008 р" ["Archaeological explorations in the city of Kharkiv and the Kharkiv region in 2008"] in *Археологічні дослідження в Україні 2008* [Archaeological Research in Ukraine 2008], D. N. Kozak, Ed.

(Institute of Archeology of the National Academy of Sciences of Ukraine, Kyiv, 2009), pp. 325–328.

226. M. I. Artamonov, *История хазар* [History of the Khazars] (State Hermitage Museum Publishing House, Leningrad, 1962).

227. S. A. Pletneva, *От кочевий к городам. Салтово-маяцкая культура* [From Nomads to Cities. Saltovo-Mayaki Culture] (Nauka, Moscow, 1967).

228. S. A. Pletneva, *Очерки хазарской археологии* [Essays on Khazar Archaeology] (Mosty Kultury, Moscow-Jerusalem, 1999).

229. M. I. Artamonov, Салкел и некоторые другие укрепления в северо-западной Хазарии [Salkel and some other fortifications in Northwestern Khazaria]. *Sov. Archaeol. 6*, 130–167 (1940).

230. G. E. Afanasyev, *Донские аланы. Социальные структуры алано-ассо-буртасского населения бассейна Среднего Дона* [Don Alans. Social Structures of the Alan-Asso-Burtas Population of the Middle Don Basin] (Nauka, Moscow, 1993).

231. A. Z. Vinnikov, "Жилые и хозяйственные постройки Маяцкого селища" ["Residential and outbuildings of the Mayatskoye settlement"] in *Маяцкое городище* [Mayatskoye Settlement] (Nauka, Moscow, 1984), pp. 95–135.

232. V. S. Flerov, *«Города» и «замки» Хазарского каганата. Археологическая реальность* ['Cities' and 'Castles' of the Khazar Khaganate. Archaeological Reality] (Mosty Kultury, Moscow-Jerusalem, 2010).

233. I. A. Baranov, *Таврика в эпоху раннего средневековья* [Taurica in the Early Middle Ages] (Naukova Dumka, Kyiv, 1990).

234. V. K. Mikheev, *Подонье в составе Хазарского каганата* [Don Region as Part of the Khazar Khaganate] (Higher School Publishing House at Kharkiv Univ., Kharkiv, 1985).

235. V. V. Koloda, Житла раннього середньовіччя у Верхньому Салтові [Houses of the early Middle Ages near Upper Saltiv]. *Archeology* **4**, 40–54 (2000).

236. V. S. Flerov, *Раннесредневековые юртообразные жилища Восточной Европы* [Early Medieval Yurt-Like Dwellings of Eastern Europe] (Institute of Archaeology of RAS, Moscow, 1996).

237. K. I. Krasilnikov, Гончарная мастерская салтово-маяцкой культуры [Pottery workshop of the Saltovo-Mayak culture]. *Sov. Archeol.* **3**, 267–278 (1976).

238. K. I. Krasilnikov, Тандыры в салтовских жилищах Подонья [Tandyrs in Saltov dwellings of the Don region]. *Sov. Archeol.* **3**, 48 (1986).

239. S. A. Pletneva, K. I. Krasilnikov, "Гончарные мастерские Маяцкого комплекса" ["Pottery workshops of the Mayatsky complex"] in *Маяцкий археологический комплекс* [Mayatsky Archaeological Complex] (Nauka, Moscow, 1990), pp. 92–139.

240. A. Z. Vinnikov, G. E. Afanasyev, *Культовые комплексы Маяцкого селища* [Cult Complexes of Mayatsky Settlement] (Voronezh State Univ., Voronezh, 1991).

241. V. S. Aksyonov, Поховальний обряд ранньосередньовічного Верхньо-Салтівського могильника: досвід статистичного аналізу [Burial rite of the early medieval Verkhno-Saltiv burial site: experience of statistical analysis]. *Archeology* **2**, 42–56 (2018).

242. S. A. Pletneva, *На славяно-хазарском пограничье. Дмитриевский археологический комплекс* [On the Slavic-Khazar Border. Dmitrievsky Archaeological Complex] (Nauka, Moscow, 1989).

243. V. S. Flerov, *Погребальные обряды на севере Хазарского каганата* [Funeral Rites in the North of the Khazar Khaganate] (Peremena, Volgograd, 1993).

244. V. S. Aksyonov, *Могильник салтово-маяцкой культуры у с. Червоная Гусаровка на Северском Донце* [Burial Ground of the Saltovo-Mayatskaya Culture Near the Village of Chervona Gusarovka on the Seversky Donets] (Kharkiv National Univ., Kharkiv, 2017).

245. O. V. Ichenskaya, “Об одном из вариантов погребального обряда салтовцев по материалам Нетайловского могильника” [“On one of the variants of the burial rite of the Saltov people based on the materials of the Netaylovskiy burial ground”] in *Древности Среднего Поднепровья* [Antiquities of the Middle Dnieper Region] (Naukova Dumka, Kyiv, 1981).

246. K. I. Krasilnikov, “О некоторых вопросах погребального обряда праболгар Среднедонечья” [“On some issues of the burial rites of the Proto-Bulgars of the Middle Donets region”] in *Ранние болгары и финно-угры в Восточной Европе* [Early Bulgars and Finno-Ugrians in Eastern Europe] (KIYALI, Kazan, 1990), pp. 28–44.

247. V. K. Mikheev, Северо-западная окраина Хазарии в свете новых археологических открытий [The Northwestern outskirts of Khazaria in light of new archaeological discoveries]. *Хазарский Альманах 3*, 74–93 (2004).

248. E. I. Savchenko, *Крымский могильник* [Crimean burial ground] (Archaeological Discoveries, Institute of Archaeology of RAS, Moscow, 1986).

249. V. S. Aksyonov, V. K. Mikheev, “Исследования Нетайловского могильника в 2002–2004 гг. экспедицией Международного Соломонова университета” [“Research of the Netaylovsky burial ground in 2002–2004 expedition of the International Solomon University” in *Культурна спадщина Слобожанщини. Історія, археологія, краєзнавство* [Cultural Heritage of Slobozhanshchina. History, Archeology, Local History] (Kharkiv National Univ., Kharkiv, 2005), pp. 134–156.

250. V. S. Aksyonov, A. A. Laptev, “Освоение населением Хазарского каганата лесостепного Подонцовья: взгляд на проблему” [“The development of the forest-steppe Podontsovye by the population of the Khazar Khaganate: A look at the problem”] in *Степи Европы в эпоху средневековья, 12* [The Steppes of Europe in the Middle Ages, 12] (Donetsk National Univ., Donetsk, 2014), pp. 25–50.

251. V. V. Koloda, S. A. Gorbanenko, *Сельское хозяйство носителей салтовской культуры в лесостепной зоне* [Agriculture of the Saltovskaya Culture Carriers in the Forest-steppe Zone] (Institute of Archeology of the National Academy of Sciences of Ukraine, Kyiv, 2010).

252. V. S. Aksyonov, “Комплексы конского снаряжения салтовского времени с начельниками (по материалам Верхнесалтовского катакомбного могильника)” [“Horse equipment complexes of the Saltiv period with headbands (based on materials from the Verkhni Saltiv catacomb burial ground)”] in *Степи Европы в эпоху средневековья 4* [The Steppes of Europe in the Middle Ages 4] (Donetsk National Univ., Donetsk, 2005), pp. 245–260.

253. A. V. Kryganov, “Вооружение и войско населения салтово-маяцкой культуры (по материалам могильников с обрядом трупосожжения)” [“Weapons and troops of the population of the Saltiv-Mayaki culture (based on materials from burial grounds with the rite of cremation)”] in *Проблемы археологии Поднепровья* [Problems of Archeology of the Dnieper Region] (Dnipropetrovsk State Univ., Dnepropetrovsk, 1989), pp. 98–111.

254. A. V. Kryganov, Військова справа ранньосередньовічних аланів Подоння [Military affairs of the early medieval Alans of Podonia]. *Archeology* **2**, 52–62 (1993).

255. N. A. Fonyakova, Лотос в растительном орнаменте металлических изделий салтово-маяцкой культуры VIII-IX вв [Lotus in the plant ornament of metal products of Saltiv-Mayaky culture VIII-IX centuries]. *Sov. Archaeol* **3**, 36–47 (1986).

256. A. V. Komar, “Происхождение поясных наборов раннесалтовского типа” [“The Origin of Belt Sets of the Early Saltiv Type”] in *Культуры степей Евразии второй половины I тысячелетия н.э. (из истории костюма). Том 2* [Cultures of the Eurasian Steppes in the Second Half of the First Millennium AD (from the History of Costume). Vol. 2], D. A. Stashenkov, Ed. (Samara Regional Museum of History and Local Lore named after P. V. Alabin, Samara, 2001), pp. 103–117.

257. A. L. Jacobson, *Керамика и керамическое производство средневековой Таврики* [Ceramics and Ceramic Production of Medieval Taurica] (Nauka, Leningrad, 1979).

258. V. S. Aksyonov, V. K. Mikheev, “Крымский импорт и хронология некоторых салтовских памятников верховий Северского Донца” [“Crimean Import and Chronology of Some Saltov Monuments of the Upper Seversky Donets”] in *Культуры степей Евразии второй половины I тысячелетия н.э. (вопросы хронологии)* [Cultures of the Eurasian Steppes of

the Second Half of the First Millennium AD (Questions of Chronology)] (Samara Regional Museum of History and Local Lore, Samara, 1998), pp. 344–357.

259. E. V. Kruglov, “Некоторые проблемы анализа особенностей обращения византийских монет VI–VIII вв. в восточноевропейских степях” [“Some problems of analysis of the peculiarities of the circulation of byzantine coins of the 6th–8th centuries in the Eastern European steppes”] in *Хазарский альманах. Том 1* [The Khazar Almanac. Volume 1], V. I. Mikheev, Ed. (Karavella, Kharkiv, 2002), pp. 79–93.

260. O. V. Ichenskaya, “Особенности погребального обряда и датировка некоторых участков Салтовского могильника” [“Features of the burial rite and dating of some sections of the Saltovsky burial ground”] in *Материалы по хронологии археологических памятников Украины* [Materials on the Chronology of Archaeological Monuments of Ukraine] (Naukova Dumka, Kyiv, 1982), pp. 140–148.

261. V. S. Aksyonov, “Погребения всадников Нетайловского могильника салтовской культуры: типология и хронология (по материалам 2003 –2010 годов)” [“Burials of horsemen of the Netaylivka burial ground of the Saltov culture: typology and chronology (based on materials from 2003–2010)”] in Степи Европы в эпоху средневековья 9 [The Steppes of Europe in the Middle Ages 9] (Donetsk National Univ., Donetsk, 2012), pp. 207–242.

262. M. V. Khoruzhaya, “Катакомбные захоронения главного Верхнее-Салтовского могильника (раскопки 2004 года)” [“Catacomb burials of the main Verkhne-Saltovsky burial ground (excavations of 2004)”] in *Степи Европы в эпоху средневековья 7* [The Steppes of Europe in the Middle Ages 7] (Donetsk National Univ., Donetsk, 2009), pp. 259–294.

263. V. S. Aksyonov, M. V. Khoruzhaya, “К вопросу о количестве катакомбных могильников у с. Верхний Салтов” [“On the Number of Catacomb Burial Grounds near the Village of Verkhniy Saltov”] in *Проблемы археологии Восточной Европы. К 85-летию Бориса Андреевича Шрамко* [Problems of Archaeology of Eastern Europe. On the 85th Anniversary of Boris Andreevich Shramko], S. I. Posokhov, Ed. (Cursor, Kharkiv, 2008), pp. 19–27.

264. A. A. Laptev, *Отчет об археологических исследованиях Бочковского могильника салтовской культуры (Волчанский район Харьковской области) в 2014 году* [Report on archaeological research of the Bochkovsky burial ground of the Saltov culture (Volchansky district of Kharkiv region) in 2014] (Institute of Archeology of the National Academy of Sciences of Ukraine, Kyiv, 2015).

265. A. A. Laptev, V. S. Aksyonov, "Погребения с «оболом мертвых» на Бочковском могильнике салтово-маяцкой культуры (по материалам раскопок 2014 г.)" ["Burials with an 'obol of the dead person' at the Bochkovo burial ground of the Saltiv-Mayaki culture (based on materials from excavations in 2014)"] in *Учні та послідовники до 80-річчя з дня народження* [Scholars and Followers Until the 80th Anniversary of the People's Day], V. S. Aksionov, V. K. Mikheev, Eds. (RA "IPIS," Kharkiv, 2017), pp. 80–97.

266. S. A. Pletneva, "Печенеги, торки, половцы" ["Pechenegs, Torks, Cumans"] in *Степи Евразии в эпоху средневековья* [The Steppes of Eurasia in the Middle Ages] (Nauka, Moscow, 1981), pp. 213–222.

267. S. A. Pletneva, *Кочевники средневековья: поиски исторических закономерностей* [Nomads of the Middle Ages: The Search for Historical Patterns] (Nauka, Moscow, 1982).

268. S. A. Pletneva, *Половцы* [Cumans] (Nauka, Moscow, 1990).

269. I. I. Kapritsyn, "Средневековые погребения из кургана № 43 могильника Мамай-Гора" ["Medieval burials from kurgan no. 43 of the Mamai-Gora burial ground"] in *Древности степного Причерноморья и Крыма* [Antiquities of the steppe Black Sea region and Crimea] (Zaporizhzhia State Univ., Zaporizhzhia, 1993), pp. 264–267.

270. S. I. Andrukh, G. N. Toshchev, "Курганы 16-19 у с. Великая Знаменка" ["Kurgans 16-19 near the Velikaya Znamenka village"] in *Старожитності степового Причорномор'я і Криму XII* [Antiquities of the Black Sea Steppe and Crimea XII], P. P. Tolochko, Ed. (Zaporizhzhia National Univ., Zaporizhzhia, 2005), pp. 106–140.

271. M. V. Elnikov, Грунтовый кочевнический могильник Мамай-Гора XIV в. из Нижнего Поднепровья [The Mamai-Gora nomadic burial ground of the 14th century from the Lower Dnipro region]. *Tatar Archeol.* **1-2**, 86–111 (2004).

272. V. S. Shelomentsev-Tersky, *Летописный Звенигород Галицкий X—XIII ст* [Chronicle of Zvenigorod Galitsky 10th-13th Centuries] (Institute of Archaeology of the Academy of Sciences of the Ukrainian SSR, Kyiv, 1981).

273. V. D. Gupalo, Ранньосередньовічні могильники Звенигорода: проблема періодизації і хронології [Early medieval burial grounds of Zvenigorod: The problem of periodization and chronology]. *Археологічні дослідження Львівського університету* **13**, 205–218 (2010).

274. S. V. Terskyi, *Археологія доби Галицько-Волинської держави* [Archeology of the Period of the Galicia-Volyn State] (Ancient World, Kyiv, 2014).

275. B. A. Shramko, V. V. Skirda, *Рождение Харькова* [Birth of Kharkiv] (Eastern Regional Center for Humanitarian and Educational Initiatives, Kharkiv, 2004).

276. B. A. Shramko, *Отчет о работе Скифо-славянской экспедиции Харьковского госуниверситета в 1960 г* [Report on the work of the Scythian-Slavic expedition of the Kharkiv State University in 1960] (Institute of Archeology of the National Academy of Sciences of, Kyiv, 1961).

277. B. A. Shramko, *Древности Северского Донца* [Antiquities of the Siversky Donets] (Kharkiv Univ. Publishing House, Kharkiv, 1962).

278. V. L. Egorov, *Историческая география Золотой Орды в XIII–XIV вв* [Historical Geography of the Golden Horde in the XIII–XIV Centuries] (Nauka, Moscow, 1985).

279. V. V. Priymak, *Северная граница буферной зоны золотоордынского времени на территории Днепровского лесостепного Левобережья* [Northern Border of the Buffer Zone of the Golden Horde Period on the Dnipro Left Bank Forest-steppe] (Antiquities of the Middle Ages of the Eurasian Forest-steppe, Voronezh State Univ., Voronezh, 2008).

280. O. B. Suprunenko, V. V. Priymak, K. M. Mironenko, *Старожитності золотоординського часу Дніпровського лісостепового Лівобережжя* [Antiquities of the Golden Horde Period of the Dnipro Left Bank Forest-steppe] (Institute of Archeology of the National Academy of Sciences of Ukraine, Kyiv-Poltava, 2004).

281. I. B. Shramko, S. A. Zadnikov, "Поселение XIV века «Олешки» на Северском Донце" ["The 14th-century settlement of 'Oleshki' on Siversky Donets bank"] in *Степи Европы в эпоху Средневековья 8, Золотоордынское время* [The Steppes of Europe in the Middle Ages 8, Golden Horde Time] (Donetsk National University, Donetsk, 2010), pp. 163–230.

282. V. K. Mikheev, *Отчет о раскопках поселения и могильника салтовской культуры у с. Маяки летом 1965 г* [Report on excavations of a settlement and burial ground of the Saltiv culture near the village of Mayaki in the summer of 1965] (Institute of Archeology of the National Academy of Sciences of Ukraine, Kyiv, 1966).

283. M. V. Elnikov, *Средневековый могильник Мамай-Сурка: по материалам исследований 1989–1992 гг* [Medieval Burial Ground of Mamai-Surka: Based on Research Materials from 1989–1992] (Zaporizhzhia State Univ., Zaporizhzhia, 2001).

284. M. V. Elnikov, *Средневековый могильник Мамай-Сурка. По материалам исследований 1993–1994 гг* [Medieval Burial Ground of Mamai-Surka. Based on Research Materials From 1993–1994] (Zaporizhzhia State Univ., Zaporizhzhia, 2006).

285. L. V. Litvinova, *Населення Нижнього Подніпров'я 12 - початку 15 ст* [Population of the Lower Dnipro Region From 12th up to 15th Centuries] (Institute of Archeology of the National Academy of Sciences of Ukraine, Kyiv, 2012).

286. A. G. Toshchev, Типология погребальных конструкций ногайского могильника Мамай-гора в Нижнем Поднепровье [Typology of funerary structures of the Nogai burial ground Mamai-Gora in Lower Dnipro]. *Scientific works of the Faculty of History of Zaporizhzhia National University* **41**, 357–362 (2014).

287. A. G. Toshchev, “Погребальный обряд поздних кочевников Нижнего Поднепровья XV–XVI ст” [“Burial rite of the late nomads of the Lower Dnipro region of the XV–XVI centuries”] in *Старожитності Степового Причорномор’я і Криму XVIII* [Antiquities of the Steppe Black Sea Region and Crimea XVIII] (Zhaporizhzhia National Univ., Zaporizhzhia, 2015), pp. 187–197.

288. S. I. Andrukh, G. N. Toshchev, *Отчет об охранных раскопках на могильнике Мамай Гора у с. В. Знаменка Каменско-Днепровского района Запорожской области в 2013 г* [Report on the security excavations at the Mamai Gora burial ground near the village of V. Znamenka, Kamensko-Dniprovsky district, Zaporizhzhia region in 2013] (Institute of Archeology of the National Academy of Sciences of Ukraine, Kyiv, 2013).

289. S. I. Andrukh, G. N. Toshchev, *Отчет об охранных раскопках на могильнике Мамай-Гора у с. В. Знаменка Каменско-Днепровского района Запорожской области в 2008 г* [Report on the security excavations at the Mamai-Gora burial ground near the village of V. Znamenka, Kamensko-Dneprovsky district, Zaporizhzhia region in 2008] (Institute of Archeology of the National Academy of Sciences of Ukraine, Kyiv, 2008).

290. V. L. Berenstam, *Отчет о раскопках в Остерском уезде Черниговской губ* [Report on excavations in the Oster district of Chernihiv province] (Institute of the History of Material Culture of the Russian Academy of Sciences, St. Petersburg, 1889).

291. V. M. Skorokhod, O. S. Heyda, O. V. Tereshchenko, “Християнські поховання на городищі Виповзівського археологічного комплексу (до питання про локалізацію церкви Воздвиження Хреста Господня)” [“Christian burials at the burial ground of the Vypovziv archaeological complex (to the question of the location of the Church of the Exaltation of the Cross of the Lord)”] in *Археологические исследования в Еврорегионе “Днепр” в 2013 г* [Archeological Research in the Dnipro Euroregion in 2013] (Bryansk State University, Bryansk, 2014), pp. 205–206.

292. V. M. Skorokhod, O. P. Motsia, Yu. M. Sitii, V. S. Zhigola, O. M. Shumei, *Науковий звіт про археологічні дослідження Виповзівського археологічного комплексу біля с. Виповзів Козелецького району Чернігівської області. Т. 1. Дослідження на городищі* [Scientific report on archaeological research of the Vypovziv archaeological complex near

the village of Vypovziv in Kozeletsky district of Chernihiv region. Vol. 1. Research at the settlement] (Institute of Archeology of the National Academy of Sciences of Ukraine, Kyiv, 2013).

293. V. M. Skorokhod, O. P. Motsya, Yu. M. Sitii, V. S. Zhigola, O. M. Shumey, *Науковий звіт про археологічні дослідження 2014 р. Виповзівського археологічного комплексу біля с. Виповзів Козелецького району Чернігівської області. Т. 1. Дослідження городища* [Scientific report on archaeological research in 2014 of the Vypovziv archaeological complex near the village of Vypovziv in Kozeletsky district of Chernihiv region. Vol. 1. Investigation of the settlement] (Institute of Archeology of the National Academy of Sciences of Ukraine, Kyiv, 2014).
